# Identification of Rapeseed (*Brassica napus*) Cultivars With a High Tolerance to Boron-Deficient Conditions

**DOI:** 10.3389/fpls.2018.01142

**Published:** 2018-08-07

**Authors:** Benjamin Pommerrenig, Astrid Junker, Isidro Abreu, Annett Bieber, Jacqueline Fuge, Evelin Willner, Manuela D. Bienert, Thomas Altmann, Gerd P. Bienert

**Affiliations:** ^1^Department of Physiology and Cell Biology, Leibniz Institute of Plant Genetics and Crop Plant Research, Gatersleben, Germany; ^2^Department of Molecular Genetics, Leibniz Institute of Plant Genetics and Crop Plant Research, Gatersleben, Germany; ^3^Department of Biology, Facultad de Ciencias, Universidad Autónoma de Madrid, Madrid, Spain; ^4^Genebank Department, Leibniz Institute of Plant Genetics and Crop Plant Research, Gatersleben, Germany

**Keywords:** boron, rapeseed, *Brassica napus*, phenotyping, boron deficiency tolerance, boron efficiency

## Abstract

Boron (B) is an essential micronutrient for seed plants. Information on B-efficiency mechanisms and B-efficient crop and model plant genotypes is very scarce. Studies evaluating the basis and consequences of B-deficiency and B-efficiency are limited by the facts that B occurs as a trace contaminant essentially everywhere, its bioavailability is difficult to control and soil-based B-deficiency growth systems allowing a high-throughput screening of plant populations have hitherto been lacking. The crop plant *Brassica napus* shows a very high sensitivity toward B-deficient conditions. To reduce B-deficiency-caused yield losses in a sustainable manner, the identification of B-efficient *B. napus* genotypes is indispensable. We developed a soil substrate-based cultivation system which is suitable to study plant growth in automated high-throughput phenotyping facilities under defined and repeatable soil B conditions. In a comprehensive screening, using this system with soil B concentrations below 0.1 mg B (kg soil)^-1^, we identified three highly B-deficiency tolerant *B. napus* cultivars (*CR2267*, *CR2280*, and *CR2285*) among a genetically diverse collection comprising 590 accessions from all over the world. The B-efficiency classification of cultivars was based on a detailed assessment of various physical and high-throughput imaging-based shoot and root growth parameters in soil substrate or in *in vitro* conditions, respectively. We identified cultivar-specific patterns of B-deficiency-responsive growth dynamics. Elemental analysis revealed striking differences only in B contents between contrasting genotypes when grown under B-deficient but not under standard conditions. Results indicate that B-deficiency tolerant cultivars can grow with a very limited amount of B which is clearly below previously described critical B-tissue concentration values. These results suggest a higher B utilization efficiency of *CR2267*, *CR2280*, and *CR2285* which would represent a unique trait among so far identified B-efficient *B. napus* cultivars which are characterized by a higher B-uptake capacity. Testing various other nutrient deficiency treatments, we demonstrated that the tolerance is specific for B-deficient conditions and is not conferred by a general growth vigor at the seedling stage. The identified B-deficiency tolerant cultivars will serve as genetic and physiological “tools” to further understand the mechanisms regulating the B nutritional status in rapeseed and to develop B-efficient elite genotypes.

## Introduction

The metalloid boron (B) is a microelement essential for growth and development of vascular plants (Blevins and Lukaszewski, 1998; Marschner, 2012). This essentiality is based on the di-ester bond formation of B with apiose residues of the polysaccharide rhamnogalacturonan II (RG-II) thereby establishing crosslinks in the cell wall’s pectin layer determining integrity and stability of cell walls (O’Neill et al., 1996, 2001). B deficiency inhibits root- and shoot meristem activity, cell elongation, and flower development, leading to reproductive failure and ultimately reduced yield (Shorrocks, 1997; Eggert and von Wirén, 2015). At the macroscopic level, these histological B deficiency-mediated disorganizations result in disease-like symptoms such as heart rot ( = dry rot or crown rot) in roots and shoots of, e.g., rapeseed or sugar beet (Bergmann, 1992; Marschner, 2012). B deficiency-derived crop failures are widespread around the globe and soils low in B [<0.25 mg B (kg soil)^-1^] are found in more than 80 countries (Shorrocks, 1997). Under physiological and most environmental soil conditions relevant for crop plant cultivation, plant available B is present as highly water soluble boric acid (H_3_BO_4_, p*K*a_1_ = 9.25). Soil pH, pore water, and humidity are major factors affecting B availability and solubility. Strategies to avoid or ameliorate effects of B deficiency usually rely on the application of B fertilizers (Goldbach et al., 2001; Marschner, 2012). The timing of fertilization is however difficult to set as B deficiency with detrimental consequences on plant growth or yield occurs often before visible symptoms appear. The reliable prediction of B deficiency in B-demanding crops is therefore difficult because B deficiency can onset either phenotypically latent and/or only locally linked to certain rapidly occurring weather conditions, such as extensive rainfall (leaching of B) or drought (Pommerrenig et al., 2015). An immediate B supply following the detection of B deficiency symptoms does not completely reverse detrimental effects. Preventative measures such as high B fertilizer dosage applications can also be problematic as the range between B deficiency and toxicity is quite narrow within one species and can be misaligned between different crop species. Slightly excessive B supply for a crop with high B demand can cause toxicity to the crop that is following in the crop rotation cycle (Gupta, 1983; Gupta et al., 1985).

*Brassica napus* is allopolyploid and originated, probably several times independently, from hybridization between the diploid *B. rapa* and *B. oleracea* genome donors about 7500 years ago (Chalhoub et al., 2014). *B. napus* was cultivated and bred in several regions of the world, and developed globally to one of the major oil crops (oilseed rape) used for animal and human consumption, industrial products, and as a biofuel source (Basunanda et al., 2010). Together with sugar beet, oilseed rape and other *Brassica* crops have the highest demand for B among crop species (Gupta, 1980). *B. napus* is extremely sensitive to B deficiency and above-described deficiency symptoms frequently occur under B limiting conditions. Most detrimental for yield, *B. napus* exhibits a “flowering without seed setting phenotype” when B deficiency occurs during reproductive development. Oilseed rape yield- and quality losses, caused by B deficiency, mainly occur in countries of Northern Europe, Canada, and China (Food and Agriculture Organization of the United Nations, Faostat^1^). Agriculturally used *B. napus* cultivars have B concentration requirements higher than 0.5 mg B (kg soil)^-1^, which exceed the concentrations in many agricultural soils (Shorrocks, 1997), mostly in China, where more than 33.3 million hectares of soils of agricultural land have lower B concentrations (Xu et al., 2001).

Studies investigating B efficiency in *B. napus* have been conducted since the 1990s (Xue et al., 1998; Stangoulis et al., 2001; Xu et al., 2001, 2002; Yang et al., 2013; Zhang et al., 2014a). Knowledge on physiological, molecular, and genetic factors influencing B efficiency only exists for a very limited number of cultivars (Zhao et al., 2012; Yang et al., 2013; Zhang et al., 2014a, 2017; Yuan et al., 2017; Zhou et al., 2017). Genetic analyses for yield-related traits under low B conditions suggested two major and various minor loci associating with B efficiency traits (Xu et al., 2001, 2002; Zhao et al., 2008, 2012; Zhang et al., 2014a,b). Recently, a strategy combining fine mapping and digital gene expression analyses identified *BnaA3.NIP5;1*, a Nodulin26-like intrinsic protein (NIP)-encoding gene as the responsible gene for one of those B efficiency loci in *B. napu*s *cv. Qingyou10* (Hua et al., 2016). BnaC4.BOR1;1c has also been described recently as an AtBOR1 homolog being crucial for the delivery of B to the inflorescences (Zhang et al., 2017). These findings are in agreement with the knowledge that boric acid-permeable NIP channel proteins in coordination with borate effluxing BOR1 transporters are essential for B uptake from the soil into roots and the subsequent translocation to shoots and flowers in monocot and dicot plants (Miwa and Fujiwara, 2010; Yoshinari and Takano, 2017).

Certain developmental stages are more prone to B-deficiency than others. Most sensitive phases are early leaf development, inflorescence emergence, and flowering. Hence, B deficiency tolerance is a complex trait that is governed by many loci, most of which act at certain restricted developmental phases. Therefore, the breeding of high-yielding oilseed rape lines and hybrids with improved B efficiency and B deficiency tolerance traits at sensitive stages is a highly sustainable approach to face the challenge of low B-containing soils and to deal with spatiotemporally varying B availabilities.

Genotypes exhibiting different or even contrasting nutrient efficiencies can be most useful for the discovery of genomic loci regulating the B nutritional status, B efficiency markers, as well as for understanding phenotypic, molecular, and physiological B deficiency responses of plants (Xu et al., 2002; Zhang et al., 2014a,b). The present study focusses on the identification of cultivars, which are tolerant to B-deficient soil conditions during the first weeks of growth, until about BBCH (“Biologische Bundesanstalt, Bundessortenamt und CHemische Industrie”) stage 15 (Lancashire et al., 1991). Genetic and physiological studies showed that *B. napus* plants are especially sensitive to B deficiency at these young growth stages and that at this point, the nutrient uptake, the tissue water content, and the seedling vigor highly depend on sufficient and continuous external soil B supply (Zhang et al., 2014b; Eggert and von Wirén, 2015). The identification of genotypes, which are B deficiency tolerant at this early developmental growth stage, would be highly desirable, as they must possess mechanisms ensuring a healthy seedling establishment even under spatiotemporally limiting B conditions. In this context, studies evaluating the basis and consequences of B deficiency would benefit from the development of stable, high-throughput compatible “B-free” soil substrate cultivation systems which allow the identification and characterization of B-efficient cultivars in defined growth conditions.

In this study, we successfully established such a soil substrate-based cultivation system as well as procedures and protocols to conduct B efficiency screenings in automated high-throughput phenotyping facilities under highly defined and repeatable soil substrate nutrient conditions. This allowed us to identify three highly B deficiency tolerant *B. napus* cultivars among a genetically diverse collection comprising 590 accessions from all over the world. Different phenotyping technologies were employed to unravel the different B-responsive shoot and root growth patterns of cultivars contrasting in their B deficiency tolerance. Moreover, elemental analyses of plants grown under various B conditions suggest a higher B utilization efficiency of identified cultivars rather than a higher B uptake capacity which is characteristic for the so far described B deficiency tolerant *B. napus* cultivars. Assaying response reactions to other nutrient deficiency treatments suggest that the cultivars identified in this study possess a specific tolerance to B-deficient conditions.

## Materials and Methods

### Plant Material

A manually assembled population of 234 spring-type and 356 winter-type *B. napus* L. *subsp. napus var. napus (Schübl. & Mart.) Thell.* accessions was used in this study (**Supplementary Data Sheet S1**). The *B. napus* cultivars were retrieved from the IPK Genbank^2^. The majority of winter-type cultivars are of the “++” type (with erucic acid and high glucosinolate content) to comprise genotypes with a broad genetic diversity. Passport data of used accessions are accessible on-line via the Genebank Information System of the IPK Gatersleben, GBIS/I^3^. Passport data include for instance the accession number, the scientific name, the accession name, the country of origin, the donor, the biological status, or the life form.

### Seed Sterilization

*Brassica napus* seeds used for the root cessation assay and the hydroponic growth analysis under different nutrient deficiencies were sterilized by adding 10 ml of 4.2% NaOCl to about 2 ml seeds in a 15 ml Falcon tube. The suspension was horizontally shaken for 10 min at room temperature. Seeds were washed three times with 10 ml sterile water and dried on sterile filter paper.

### Seed Germination

Seeds germinated on sterile agar medium containing a ½ strength modified Hoagland solution plus 0.3% Agar–Agar (Carl Roth, Germany) after stratification at 4°C for 2 days in the dark. Modified Hoagland solution used for germination contained the following nutrient salts (final concentration in 1× modified Hoagland in parentheses): MES (1 mM) pH 5.8, Ca(NO_3_)_2_ (2.5 mM), MgSO_4_ (2.5 mM), KNO_3_ (2.5 mM), KH_2_PO_4_ (0.5 mM), FeNaEDTA (5 μM), MnCl_2_ (2.25 μM), ZnSO_4_ (1.9 μM), H_3_BO_3_ (0.625 μM), CuSO_4_ (0.15 μM), and (NH_4_)_6_Mo_7_O_24_ (0.05 μM). Two to three days after germination (DAG), seedlings were transferred to an according medium and cultivated in a growth cabinet (Percival Scientific) with 22°C light and 19°C dark temperatures and a 10-h light period with a light intensity of 120 μmol photons m^-2^ s^-1^ at 60% humidity.

### Soil Substrate Preparation

Soil substrate was a white-peat volcanic clay mixture, a so-called “Fruhstorfer Nullerde” with B levels below 0.1 mg B (kg soil)^-1^ (as from now on referred to as zerosoil-substrate) (Eggert and von Wirén, 2017). This zerosoil-substrate was supplemented with 0.5% CaCO_3_ and 0.3% CaO. Calcium concentration was adjusted by mixing 20 kg soil with 1 l of CaCO_3_ (100 g l^-1^) and 1 l of CaO (60 g l^-1^) in a clean cement mixer for 10 min. Thereafter, soil substrate was manually mixed each other day. About 1–2 weeks after preparation clumps were sieved out and the soil (dry matter: ∼30%) was used for the experiments.

### Soil Cultivation Greenhouse Experiments With Defined B Conditions

Rapeseed plants germinated and have been grown in pots (∅ 16 cm) filled with 1 kg of zerosoil-substrate in the greenhouse. Plants were watered per pot every 2 days with 250 ml 1× Hoagland solution containing either 2 mM (B 2), 200 μM (B 1), or 0 μM boric acid (B 0) (see **Supplementary Data Sheet S2**). All solutions additionally contained the following concentrations of nutrients: 7.5 mM NH_4_NO_3_, 2.9 mM KH_2_PO_4_, 850 μM MgSO_4_, 340 μM K_2_SO_4_, 320 μM MnCl_2_, 52 μM CuSO_4_, 20 μM NaFeEDTA, 4.5 μM ZnSO_4_, and 0.4 μM NaMoO_4_. Nutrient solutions were prepared out of 100× stock solution mixes with MilliQ water. 100× stock solution mixes were prepared in 1 l plastic bottles (Nalgene): 100× macro element mix (60 g l^-1^ NH_4_NO_3_, 40 g l^-1^ KH_2_PO_4_, 20 g l^-1^ MgSO_4_, 6 g l^-1^ K_2_SO_4_), 100× micro element mix (4 g l^-1^ MnCl_2_, 1.3 g l^-1^ CuSO_4_ × 5H_2_O, 1.3 g l^-1^ ZnSO_4_ × 7H_2_O, 8.5 mg l^-1^ NaMoO_4_), and 100× NaFeEDTA mix (0.7 g l^-1^). No glassware was used in any process during the preparation of the nutrient solution or the irrigation of the plants to prevent additional input of B into the growth system. For growth analysis at toxic B soil concentrations, plants were germinated in zerosoil-substrate buffered with CaO and CaCO_3_ and fertilized with nutrient solution as mentioned-above supplemented with either sufficient (4.2 mg B per kg zerosoil-substrate), or toxic (360 or 420 mg B per kg zerosoil-substrate) B levels. Nutrients were supplied to the soil in four subsequent waterings. Thereafter, plants were fertilized weekly with nutrient solution without B. Greenhouse conditions were set to long-day conditions (16 h day/8 h night) at 22/18°C, 60/70% relative humidity, and ∼250 μmoles photons PAR m^-2^ s^-1^ light intensity.

### Soil Cultivation Growth Experiments in an Automated High-Throughput Plant Phenotyping Facility

The automated phenotyping experiment was conducted at the IPK Gatersleben employing an automated plant transport and imaging system (the IPK automated plant phenotyping system for small plants) situated in a plant growth chamber for controlled environmental conditions (Junker et al., 2015). The experiment included 13 selected accessions (**Supplementary Data Sheet S3**) with eight replicates per cultivar and treatment (–B, +B, and ++B). Replicates were distributed throughout the system using a randomized block design. Ten centimeter-diameter pots were filled with ca. 190 g of zerosoil-substrate supplemented with 0.5% (w/w) CaCO_3_ and 0.3% (w/w) CaO (prepared as described above). Zerosoil-substrate-filled pots were soaked with 250 ml of nutrient solution (as given above; according to treatment –B, +B, and ++B) prior to sowing. Seeds of analyzed rapeseed cultivars were stratified on wet filter paper for 2 days at 4°C in the dark and placed onto prepared zerosoil substrate. Pots were kept for 2 days at constant temperature (15°C) to favor germination. For the germination, the phytochamber conditions were set to long-day conditions (16 h day/8 h night) at 20/17°C, 60/70% relative humidity, and ∼260 μmoles photons PAR m^-2^ s^-1^ light intensity. After 5 days plants were grown with a higher light intensity (450 μmoles photons PAR m^-2^ s^-1^ light intensity). Plants were watered every day to readjust to 70% field capacity, alternatively with double distilled water using the automated weighing and watering system, or with manually added nutrient solution (to prevent B contamination). Manual applications of nutrient solution were done at day 5 (15 ml/pot), 7 (25 ml/pot), 10 (30 ml/pot), 12 (50 ml/pot), 14 (50 ml/pot), and 16 (50 ml/pot) after sowing (DAS). Nutrient solution consisted of 1× modified Hoagland solution containing no B (–B), +B (226 μM), and ++B (3 mM), prepared as mentioned above. Nutrient solution was pre-treated over-night with 3 g/l Amberlite IRA-743 (Sigma–Aldrich) to remove B traces. For the +B and ++B condition, boric acid was re-supplied after the Amberlite IRA-743 treatment. Between 5 and 13 DAS top and side view images of all plants were taken daily in the visible range of the light spectrum (VIS), and of static fluorescence signals (FLUOR) as described earlier in Junker et al. (2015). At the end of the experiment (17/18 DAS) shoot fresh weight (FW) was measured on single-plant basis by cutting the shoot directly above ground level. Different shoot fractions [cotyledons, oldest vegetative leaf (L1), third vegetative leaf (L3), rest of leaves (RL), and stem] were collected and stored at -80°C until further characterization [i.e., dry weight (DW), element content, or sugar analysis]. DW was measured after drying the material at 65°C for 48 h. Macro- and micro nutrient concentrations in the zerosoil-substrate have been quantified after the growth experiment (**Supplementary Data Sheet S4**).

### *In vitro* Agar Plate Root Cessation Assay

Sterilized seeds of the 229 spring-type *B. napus* cultivars (**Supplementary Data Sheet S1**) were pre-germinated in sterile plastic containers on a medium containing ½ strength Hoagland solution and 0.3% Agar–Agar (Carl Roth). Seeds were stratified without light at 4°C. After 2 days, germinating seeds were moved to a growth cabinet with 22°C light and 19°C dark temperatures and a 10-h light period with a light intensity of 120 mmol photons m^-2^ s^-1^ for <48 h before comparable seedlings with identical radicle length (about 3–5 mm) were transferred onto 12 × 12 cm plastic plates filled with 50 ml medium (½ strength modified Hoagland solution, 0.6% Agar–Agar, pH 5.8) supplemented with different B concentrations (5 μM boric acid = B-sufficient condition or 0.01 μM boric acid = B-deficient condition). To reduce B contamination in the Agar–Agar-based growth medium to a minimum, 3% (w/v) B chelating agent Amberlite IRA-743 (Sigma–Aldrich) was added to the non-sterile Hoagland medium in plastic flasks and the medium incubated overnight on a horizontal shaker. On the next morning, Amberlite IRA-743 had settled and the medium was carefully decanted into new plastic flasks for autoclaving. B concentrations were adjusted by adding autoclaved boric acid to final concentrations of 5 μM (B-sufficient condition) or 0.01 μM (B-deficient condition). The layer of Hoagland medium in the plastic plates was sloped. Five seedlings (radicle between 3 and 5 mm) were positioned at about 4 cm from the top of the plastic plate then being sealed with Leukopor tape (Smith and Nephew). Seedlings were grown on vertically oriented plastic plates in above-described growth cabinet conditions. Main root depth of all seedlings was marked directly after the transfer and each 24 h thereafter on the plastic plate. Five days after the transfer, plastic plates were scanned [Epson Expression 10000XL scanner (Seiko Epson)] for the documentation of the root growth and daily root-depth-gain was quantified. Each of the 229 accessions was assayed by three biological replications of up to five seedlings per accession. Within each experiment, the spatial arrangement of plastic plate replications of the different accessions was randomized in the growth cabinet.

### Hydroponic Growth Conditions for the Analysis of the Root System Architecture

Seedlings of the different *B. napus* accessions, with a radicle length of about 3–5 mm, which have been germinated as described above, were transferred onto a cut 200 μl pipette tip filled with 200 μl ½ strength modified Hoagland (conc. as above except for: 0.625 μM B) Agar–Agar medium. Pipette tips were inserted in a punched lid of 50 ml Falcon centrifuge tubes which were filled with 50 ml ½ strength modified Hoagland solution (control) or 50 ml ½ strength modified Hoagland solution without the macro- or micronutrient for which the nutrient deficiency response was assayed. Seedlings were cultivated in above-described growth cabinet conditions and Hoagland solution was refilled when necessary. After 7–8 days of growth, plants were transferred to a transparent plastic film, the roots were separated such that single roots were clearly distinguishable from one another, and scanned in gray scale at 400 dots per inch resolution using an Epson Expression 10000XL scanner (Seiko Epson). After scanning, shoots and roots were separated and weighted. Five to six root systems per cultivar and nutrient deficiency condition were analyzed using WinRhizo Pro version 2007d (Regent Instruments). The following root traits were determined and calculated: primary root (PR) length, first-order lateral root (1LR) number, total 1LR length, average 1LR length, and 1LR density. The experiment has been independently performed twice with similar results. Significance was determined using one-way ANOVA (*p* < 0.05).

### Boron Use Efficiency Ranking

The relative efficiency indices of B use [B-efficiency indices (BEIs)] of the 590 *B. napus* cultivars (**Supplementary Data Sheet S1**) were calculated from the ratio between the mean plant DW under B 0 (DW_*B*0_) and mean DW under B 1 (DW_*B*1_) multiplied by the ratio between the mean length of the first vegetative leaf (L1) under B 0 (L1_B0_) and under B 1 (L1_B1_) according to the following formula:

$$BEI=\frac{{DW}_{B0}}{{DW}_{B1}}\times \frac{{L1}_{B0}}{{L1}_{B1}}$$

At least 10–12 plants per accession and condition were used for the calculation of the mean DW_*B*0_, DW_*B*1_, L1_B0_, and L1_B1_ values. DW_*B*0_, DW_*B*1_, L1_B0_, and L1_B1_ values have been determined at BBCH13–BBCH17 of plants growing under B 0 or B 1 conditions.

### Carbohydrate Analyses

Soluble sugars glucose, fructose, and sucrose were extracted two-times for 60 min at 80°C in 1 ml of 80% ethanol in 2 ml reaction tubes from discs (1 cm in diameter, 50–100 mg FW) punched out of vegetative *B. napus* leaves at 5-, 10-, 15-, 20-, and 25-DAG. Clear ethanolic extracts were vacuum-evaporated until dryness in a Speed-Vac concentrator (Eppendorf) at 45°C. Pellets were resuspended in 200 μl double-distilled water on a horizontal shaker for 15 min at 4°C. Sugars were quantified from 10 μl solution using the enzymatic NAD^+^-dependent conversion of glucose-6-phosphate to 6-phosphogluconate as described in Kim et al. (2013).

### Image Processing and Statistical Analysis

The Integrated Analysis Plattform (IAP; Klukas et al., 2014) was used for automated image pre-processing and segmentation as well as for the extraction of 12 relevant biomass-related, architectural, and color-related features (**Supplementary Data Sheet S5**) in the automated high-throughput plant phenotyping experiment. Principal component (PC) analysis was computed on the basis of the means of all replicates for each cultivar, trait, and imaging day using the GREPEL R package. Analysis of variance with *post hoc* Bonferroni test was performed using GenStat (18th edition).

### HR–ICP–MS Analysis

Whole seeds or 10–20 mg of oven (65°C)- or freeze-dried *B. napus* plant material were analyzed for their elemental composition as described in Eggert and von Wirén (2015).

## Results

### Establishment of a B-Deficient Soil Substrate Growth System Suitable for High-Throughput Screening

In this study, we established a growth cultivation system in which plants can be grown on non-sandy soil substrate with clearly defined B concentrations. To this aim diverse soil substrates have been assayed for low hot-water extractable B concentrations and their ability to serve as a suitable soil substrate for normal plant cultivation. We selected the “Fruhstorfer Nullerde” soil substrate (zerosoil-substrate) based on the criteria that it has B levels below 0.1 mg B (kg zerosoil-substrate)^-1^. Soil B and general nutrient concentration determination demonstrated stable nutrient compositions over independently acquired zerosoil-substrate batches. A zerosoil-substrate fertilization protocol (see the “MATERIALS and METHODS” section) was elaborated to generate a soil substrate which allowed *B. napus* growth identical to that on other typically used commercially available soil substrates.

### Identification of B Deficiency Tolerant *Brassica napus* Cultivars

**FIGURE 1:**
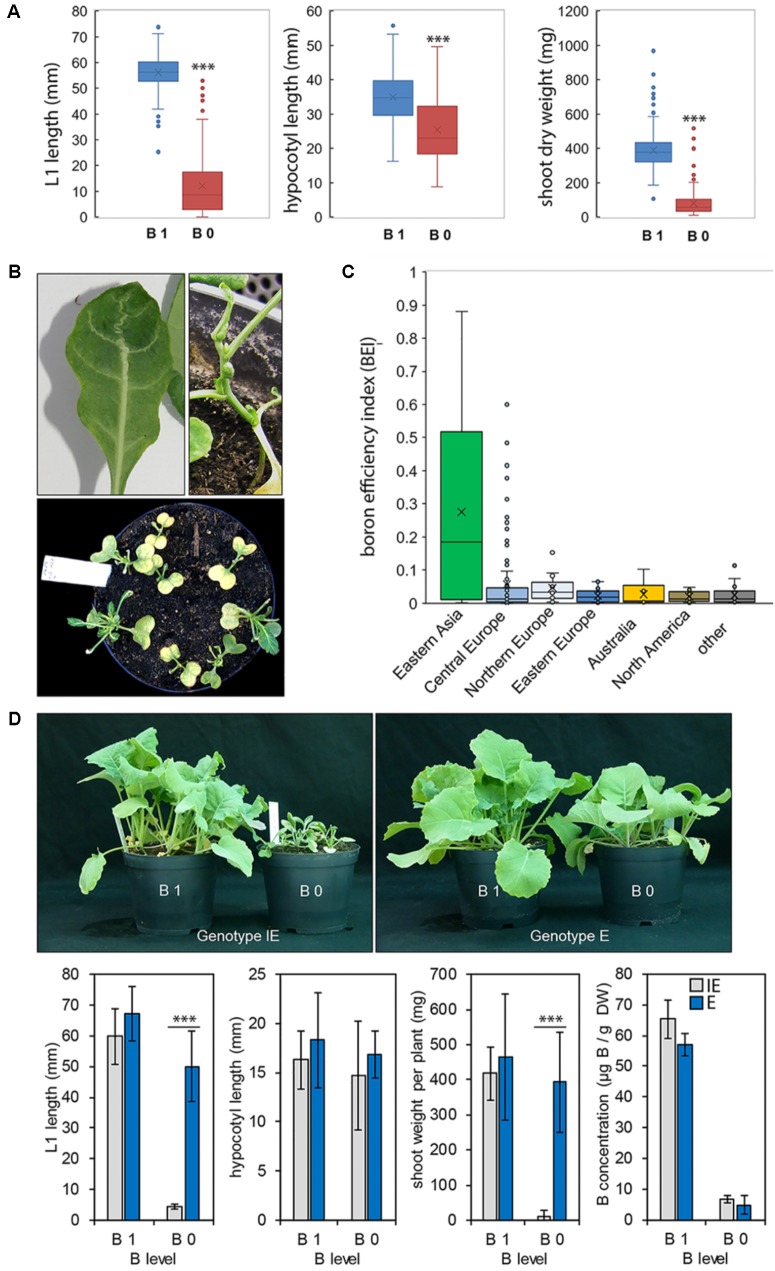
**(A)** Boxplots showing the distribution of boron (B) efficiency parameters recorded for 234 spring-type *Brassica napus* accessions. Left panel: length of leaf 1 ( = L1) in millimeters. Middle panel: hypocotyl length in millimeters. Right panel: shoot dry weight (DW) in milligrams. Crosses inside boxes show average values, horizontal lines within boxes mark the median value. Each boxplot consists of 234 parameter average values. Each parameter average value derives from *n* ≥ 10 plants. Significance between treatments (B 0/B 1) was calculated using *t-test*: ^∗∗∗^*p* < 0.001. **(B)** Exemplary pictures of plants of B-inefficient *B. napus* cultivars grown on B-deficient zerosoil-substrate and displaying B deficiency phenotypes such as arrested shoot apical meristem growth and leaf- and vasculature deformations. **(C)** Origin-dependent distribution of B efficiency indices (BEIs), calculated from the B 0/B 1 ratios of the B-related parameters L1 length and DW. Crosses inside boxes show average values, horizontal lines inside boxes mark the median value. Each boxplot consists of the BEI average values from *n* ≥ 10 plants. **(D)** Phenotypes (top panel), growth parameters (lower panel), and B concentrations (lower panel) in leaves of 25-day-old plants of B-inefficient (*CR2262* = IE) and B-efficient (*CR2267* = E) *B. napus* cultivars growing on zerosoil-substrate containing sufficient (B 1) or deficient (B 0) B levels. Growth parameters and B concentrations are means ± SD from *n* = 12 plants. Significance was calculated using *t-test*: ^∗∗∗^*p* < 0.001.

We selected a heterogeneous collection of 234 spring-type and 356 winter-type *B. napus* accessions from the Genebank of the IPK Gatersleben^4^ for the analysis of B-dependent growth of the individual accession (**Supplementary Data Sheet S1**). These cultivars represent a set of accessions from all over the world including cultivation areas with B-sufficient and B-deficient soils. Spring- and winter-type, Asian- and European-type, and oil- and leaf-type cultivars were included in the survey, also comprising 93 accessions (22 spring- and 71 winter-type cultivars) of a genetically divergent core collection (marked with “c” in **Supplementary Data Sheet S1**) (Lühs et al., 2003). The majority of the cultivars were from the ++ type [high glucosinolate (+) and erucic acid (+) levels] being genetically more diverse then their 00 (low glucosinolate and low erucic acid levels), 0+ (high glucosinolate levels), and +0 (high eruic acid levels) progenies being bred first from the 1970s onward. Genotypes were grown on soil substrate with very low [B 0; ≤0.1 mg B (kg zerosoil-substrate)^-1^] or adequate B supply [B 1; ≥2.4 mg B (kg dry zerosoil-substrate)^-1^]. Both conditions contained additionally a full supplementation of all essential macro- and micronutrients (see the “MATERIALS and METHODS” section). Most plants grown under the B 0 condition exhibited strong B deficiency symptoms such as an impaired leaf elongation and arrested shoot meristematic outgrow (**Figure 1B**). Development of the first emerging leaf (L1), hypocotyl length, and total shoot DW of most genotypes were significantly negatively affected (**Figure 1**). L1 length, hypocotyl length, and shoot DW were recorded 25 DAG for all genotypes (**Figure 1**). BEIs were calculated for the different cultivars from the B 0 / B 1 ratios of the B-dependent parameters “L1 length” and “DW” for all cultivars (for a complete list of all tested cultivars and their BEIs see **Supplementary Data Sheet S1**). We classified accessions with a BEI ≥ 0.7 as B-efficient and accessions with lower BEIs as B-inefficient. Interestingly, no winter-type cultivar could be classified as B-efficient using this cut-off BEI (**Supplementary Data Sheets S1**, **S6**). Among the spring-type accessions, B efficiency distribution was dependent on the geographical origin of the accessions showing a higher percentage of high BEIs for cultivars originating from the eastern Asiatic countries Japan, Taiwan, or China (**Figure 1C**). BEIs determined for members belonging to the core collection of Lühs et al. (2003) distributed evenly over the whole BEI range (**Supplementary Data Sheet S1**). Three cultivars with very high BEI have been identified, namely *cv. CR2267* (BEI = 0.7059), *cv. CR2280* (BEI = 0.7039), and *cv. CR2285* (BEI = 0.8800). These cultivars have been classified as B-deficiency tolerant cultivars, and are different from those described in previous screenings (Xue et al., 1998; Stangoulis et al., 2001; Xu et al., 2001, 2002; Yang et al., 2013; Zhang et al., 2014a).

The two cultivars *CR2262* (IE, B-inefficient, BEI = 0.0005) and *CR2267* (E, B-efficient, BEI = 0.7059) contrast in their B deficiency tolerance but possess similar shoot DW and L1 lengths under B-sufficient (B 1) conditions. The B accumulation in shoots of these contrasting accessions was dependent on the B supply level (B 0 or B 1), but not on the genotype (**Figure 1D**). Therefore, these two cultivars were selected for detailed analyses of their B-dependent shoot and root development, B nutritional status, and carbohydrate accumulation described later.

### Assessment of B-Dependent Growth Dynamics of Contrasting *Brassica napus* Genotypes in an Automated Plant Phenotyping Facility

**FIGURE 2:**
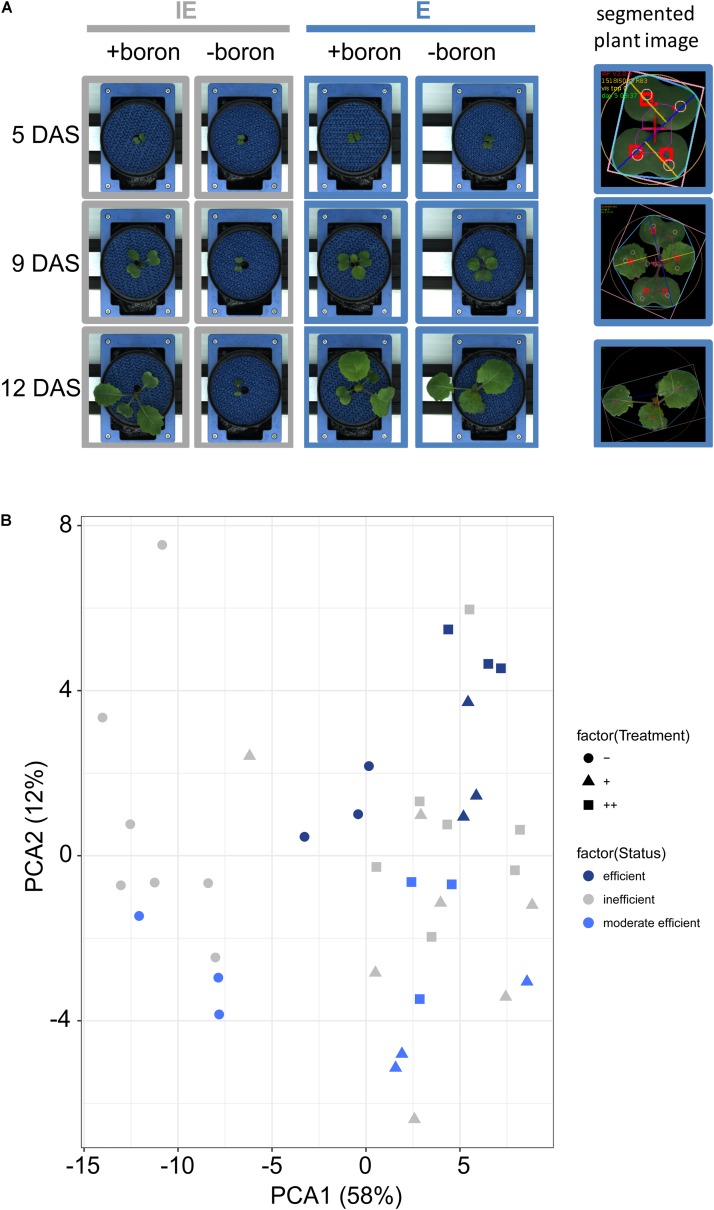
B-dependent growth behavior and analyses of *B. napus* cultivars grown in a LemnaTec cultivation system. **(A)** Left panel: Exemplary pictures of selected cultivars [IE (*CR2262*) = B deficiency-sensitive cultivar; E (*CR2267*) = B deficiency-tolerant cultivar] growing in B-deficient (-boron) or B-sufficient (+boron) zerosoil-substrate taken at days 5, 9, or 12 after sowing (DAS). Right panel: segmented plant images which are used for image analysis. **(B)** Principal component analysis (PCA) for 13 rapeseed cultivars based on recorded imaging-based growth parameters. Symbols indicate different B soil substrate conditions [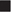 = B-deficient (-), 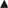 = B-sufficient (+) or 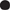 = B-surplus (++) conditions].

The cultivation and repeated imaging of selected *B. napus* accessions in an automated phenotyping system offered the possibility to precisely quantify the temporal dynamics of plant biomass-related, architectural, and color-related parameters (**Figure 2A**), which were hardly measurable manually in the prior screening approach (**Figure 1**). The cultivation was optimized for a camera-based quantification of the projected leaf area (**Figure 2A**). Thirteen cultivars, each with eight replicates per treatment, were used in the phenotyping approach including the three highly B-efficient accessions (*CR2267* = E, *CR2280* = E2, and *CR2285* = E3), and 10 additional B-inefficient accessions (**Supplementary Data Sheet S3**). Also, plants of *cv. Darmor-PBY018* (herein termed as “D”) which is genetically very similar to the recently sequenced winter-type cultivar “*Darmor-bzh*” (Chalhoub et al., 2014; Schmutzer et al., 2015) have been included in this analysis. In analogy to previous analyses, we set up three different B conditions, that are deficient [(-) <0.1 mg B (kg zerosoil-substrate)^-1^], adequate [(+) = 1.4 mg B (kg zerosoil-substrate)^-1^], or excessive [(++) = 37.4 mg B (kg zerosoil-substrate)^-1^; excessive but non-toxic at the monitored developmental stages] B conditions (described values are calculated values; **Supplementary Data Sheet S4** lists the determined values). For validation of the imaging and image analysis pipeline the image-derived biomass-related parameter “projected leaf area from the top view” extracted from images taken at 13 DAS was correlated with plant FW determined at 17/18 DAS (**Supplementary Data Sheet S7**). A highly significant positive correlation (*R* = 0.928, *p* < 0.001, **Supplementary Data Sheet S7**) confirmed the experimental setup and analysis pipeline. In general, results of the automated phenotyping confirmed the results of the initial screen and the B-deficiency tolerance classification (**Figure 2A**). Plants from the B-inefficient cultivars (for example, *CR2262* = IE in **Figure 2A**) displayed clearly visible B-deficiency symptoms and showed growth arrest at 9 DAS when grown under the (-) condition. No visible differences were detected from plants grown under (+) or (++) conditions (**Figure 2A**). For further analysis, a set of 12 B deficiency relevant imaging-based features was selected comprising biomass and color-related traits. In a PC analysis on the basis of these traits measured at all imaging days, the PC1 clearly separated the treatments for the B-inefficient cultivars (**Figure 2B**). In contrast, for B-efficient cultivars the different B treatments were not separated and clustered together (**Figure 2B**). PC1 explained 58% of the detected phenotypic variation and was mainly determined by biomass-related parameters (such as border length, **Supplementary Data Sheet S8A**). This parameter represents the number of pixels at the border between foreground (plant) and background and is highly related to biomass. This is supported by the fact that we observed a highly significant positive correlation between the traits “border length” and “projected leaf area from the top view” extracted from images of the whole growth period (*R* = 0.928, *p* < 0.001, **Supplementary Data Sheet S8B**). PC2 explained 12% of the phenotypic variance and was mainly determined by color-related traits describing intensity and coloration of plant pixels as determined in different color spaces (HSV, RGB, and LAB). No clear separation of cultivars, their efficiency status, and treatments was found, although cultivars with low resistance to B deficiency tended to cluster separated from the efficient cultivars.

**FIGURE 3:**
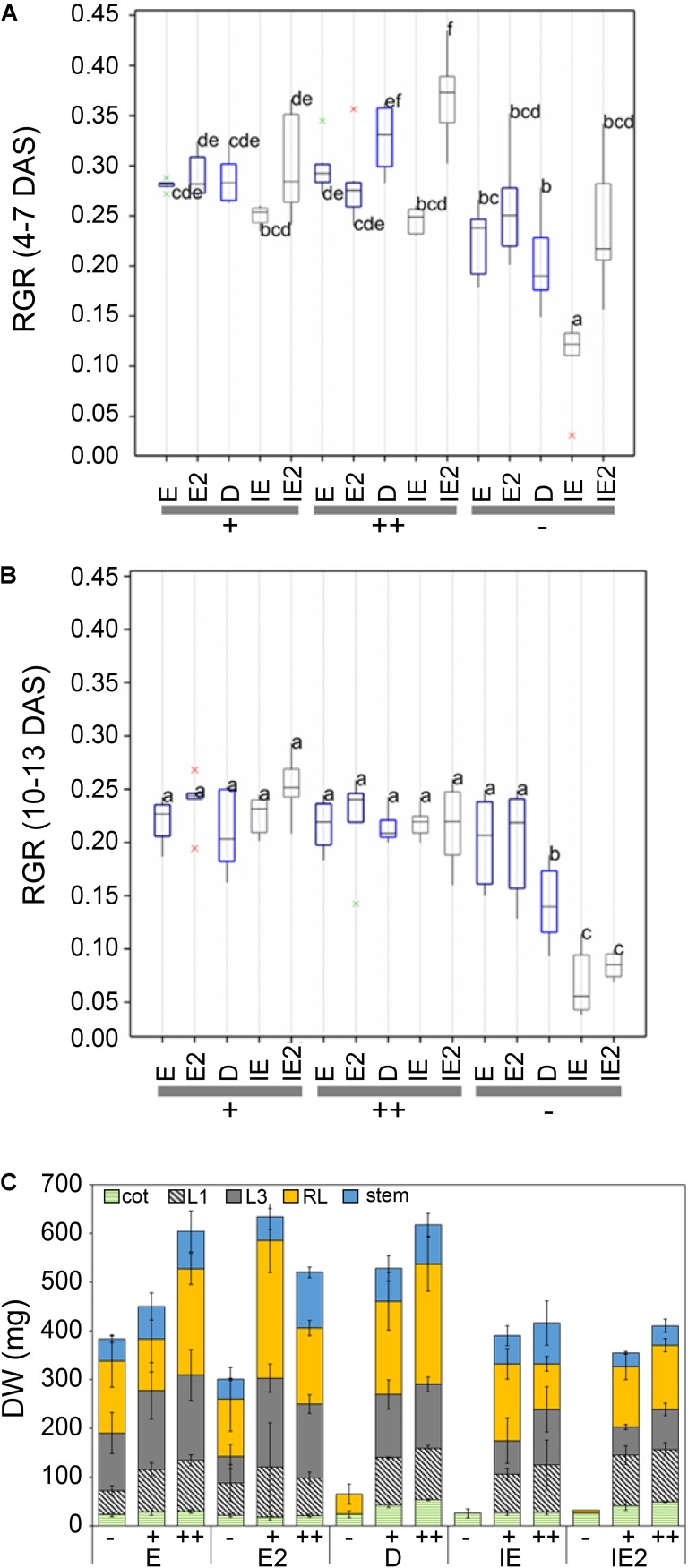
**(A)** B-dependent relative growth rates (RGR) based on the measurement of the leaf surface area (border length) of different B-efficient (E = *CR2267*, E2 = *CR2280*), moderate (*Darmor*; D), or inefficient (IE = *CR2262*, IE2 = *CR3153*) *B. napus* cultivars recorded in the LemnaTec facility at early (4–7 DAS) **(A)**, or later (10–13 DAS) **(B)** growth stages. Different letters indicate significantly different RGRs. Red and green crosses mark outliers outside the 5 and 2.5% range of the standard deviation of the value distribution, respectively. **(C)** Measurements of aboveground physical growth parameters of *B. napus* plants grown in the automated phenotyping facility. DW per plant part [cotyledons, leaf 1 (L1), leaf 3 (L3), remaining leaves (RL), stems] of indicated *B. napus* cultivars grown under B-deficient (–), B-sufficient (+), or B-surplus (++) conditions. DW values are means ± SD from *n* = 8 plants.

The B deficiency-induced phenotypes of inefficient cultivars were characterized by a strong retardation or arrest of growth at cotyledon stage. In order to assess differences in B deficiency-induced growth patterns, relative growth rates (RGR) were calculated on the basis of the “projected top view leaf area” values according to Poorter and Lewis (1986) for two time windows during the phenotyping experiment: early stress (4–7 DAS) and late stress (10–13 DAS) (**Figures 3A,B**). These analyses revealed that the inefficient cultivars IE (*CR2262*), IE2 (*CR3153*), and D (*Darmor-PBY018*) differed in their B-dependent growth patterns. In contrast to the IE cultivar, young plants of the D and the IE2 cultivar did not exhibit different RGRs before the emergence of the first vegetative leaf (L1) during growth from 4 to 7 DAS when compared to the efficient cultivars (**Figure 3A**). At later time points, at 10–13 DAS, when cotyledons were fully expanded, and first vegetative leaves started to emerge in plants grown under (+) or (++), also IE2 responded with a complete growth arrest on the (-) condition (**Figure 3B**). Plants of the B-inefficient (IE, IE2, D) accessions again showed drastically retarded growth and development under B-deficient (-) condition compared to the (+) or (++) condition. Growth dynamics were not altered between accessions under normal B conditions (+) as well as in response to excess B (++) (**Figure 3B**).

At the (-) condition FW and DW of the B-inefficient cultivars were reduced more than 90% (**Figure 3C** and **Supplementary Data Sheet S9**). The (-) condition also negatively affected the water content of the accessions IE and IE2 (**Supplementary Data Sheet S9**). However, under the conditions used in the automated phenotyping facility, the (-) condition also affected FW of two B-efficient accessions (E and E2), which responded with a loss of about 50% FW under this condition in comparison to the (+) condition (**Supplementary Data Sheet S9**).

### Elemental Analysis of Contrasting *Brassica napus* Genotypes Grown Under Different B Soil Conditions

**FIGURE 4:**
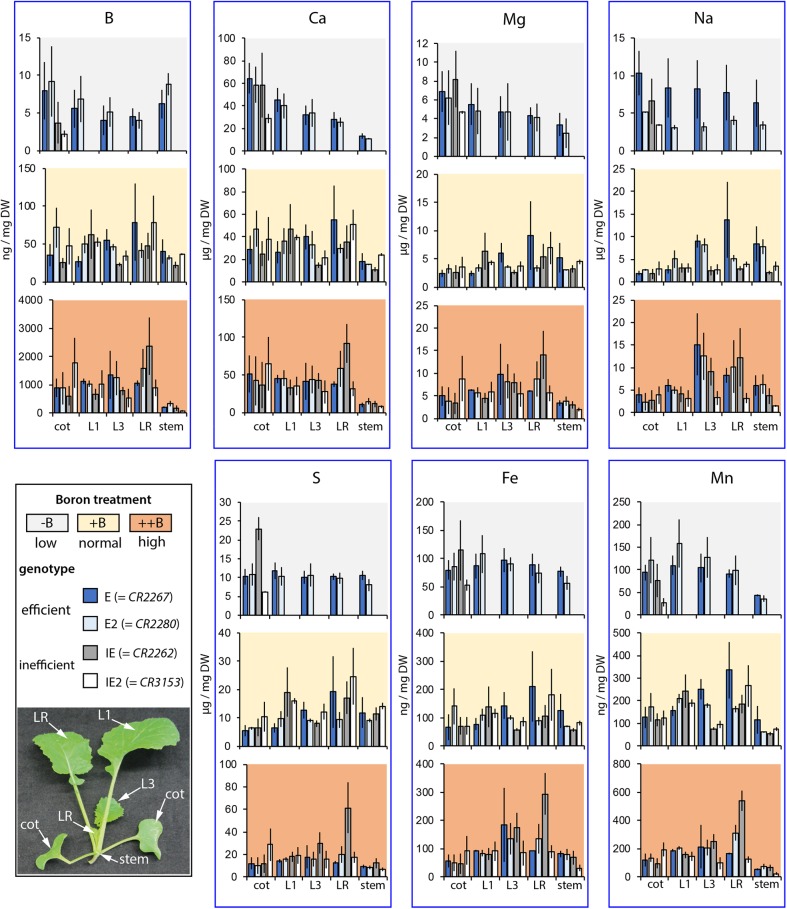
Macro- and microelement concentrations of different *B. napus* plant parts. HR–ICP–MS-based element analysis of different aerial *B. napus* plant parts of two B deficiency tolerant (E = *CR2267*, E2 = *CR2280*) and two B deficiency sensitive (IE = *CR2262*, IE2 = *CR3153*) cultivars grown in an automated phenotyping facility under B-deficient (–), B-sufficient (+), or B-surplus (++) conditions. Bars represent concentrations of specific plant parts or organs (cot, cotyledon; L1, first vegetative leaf; L3, third vegetative leaf; RL, remaining leaves; stem, remaining stem tissue without leaves). Values show mean element concentrations in ng or μg per mg DW from *n* = 3 biological replicates ± SE.

Elemental analyses of E, E2, IE, and IE2 accessions grown on the zerosoil-substrate, which have been prepared and fertilized according to our developed protocol, proved that the growth substrate is mimicking standard soil nutrient conditions (**Supplementary Data Sheet S4**) as macro- and micronutrient contents and concentrations were matching values which have been determined to be normal for *B. napus* in a diversity panel of 387 accessions (Thomas et al., 2016). Moreover, analysis of elemental contents of different organs of E, E2, IE, and IE2 plants grown under the different B soil conditions revealed that the two B-efficient cultivars accumulated B to similar concentrations in a soil B-dependent manner (**Figure 4**). Interestingly, B-tissue concentrations of phenotypically almost normal E and E2 plants, grown under B-deficient conditions, represent values which were below the critical B tissue concentration (Huang et al., 1996). The different soil B conditions affected the accumulation of other nutrients, namely S and Fe which accumulated to higher concentrations in young leaves in a soil B-dependent manner (**Figure 4**). Interestingly, Na concentrations and contents simultaneously decreased with higher soil B levels in the cotyledons of these plants indicating altered transport and distribution of nutrients via the vasculature (**Figure 4** and **Supplementary Data Sheet S10**). Ca concentrations were of particular interest, because Ca is also able to crosslink pectic polysaccharides to regulate cell wall stability and flexibility (Hepler, 2005). We measured higher Ca concentrations in cotyledons, but not in other organs of B-efficient plants grown under the low (-) B conditions compared to the normal (+) B conditions. This suggest that the consequences of the lack of B in the E and E2 cultivars are not compensated by an increased level of Ca.

**FIGURE 5:**
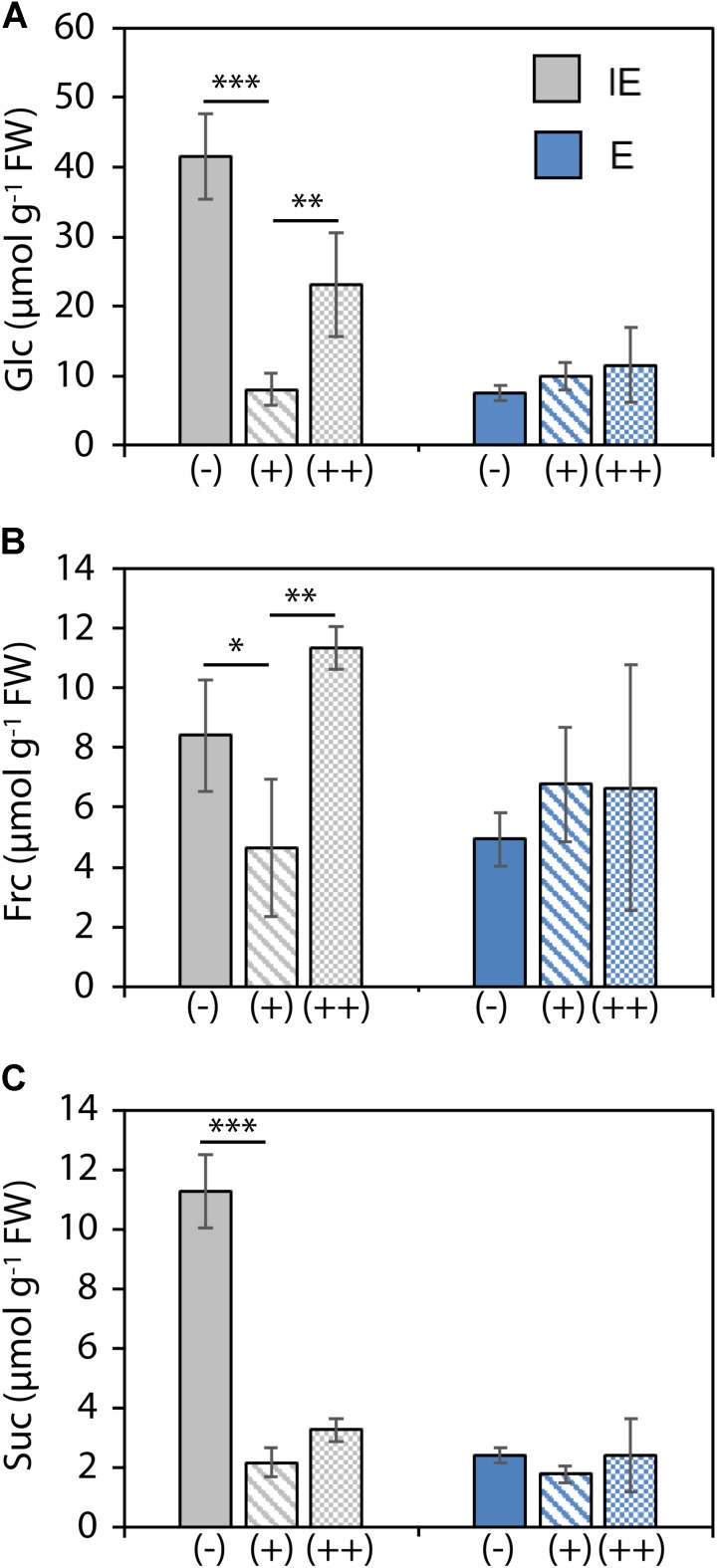
Soluble sugar concentrations [Glc, glucose **(A)**; Frc, fructose **(B)**; and Suc, sucrose **(C)**] in cotyledons from *B. napus* plants of B-deficiency tolerant (E = *CR2267*) and B-deficiency sensitive (IE = *CR2262*) cultivars grown in an automated phenotyping facility under B-deficient (–), B-sufficient (+), or B-surplus (++) conditions. Values are means ± SD from *n* = 4 plants. Significance was calculated using *t-test*: ^∗∗∗^*p* < 0.001, ^∗∗^*p* < 0.01, ^∗^*p* < 0.05.

Analysis of soluble sugars in cotyledons of plants from the IE and E cultivar showed differential B-dependent sugar accumulation. Cotyledons of IE plants accumulated drastically increased monosaccharide and sucrose levels under B-deficient (-) in comparison to B-sufficient (+) growth conditions. Under the (++) B condition, such sugar increase could only be measured for glucose and fructose but not for sucrose, indicating different carbon fixation and carbohydrate transport-related responses to either low or excess B. Plants from the E cultivar on the other hand were unaffected in their sugar accumulation under both low or excess B (**Figure 5**).

### Screening for B-Efficient Root Growth at the Seedling Stage: Root Cessation Assay

**FIGURE 6:**
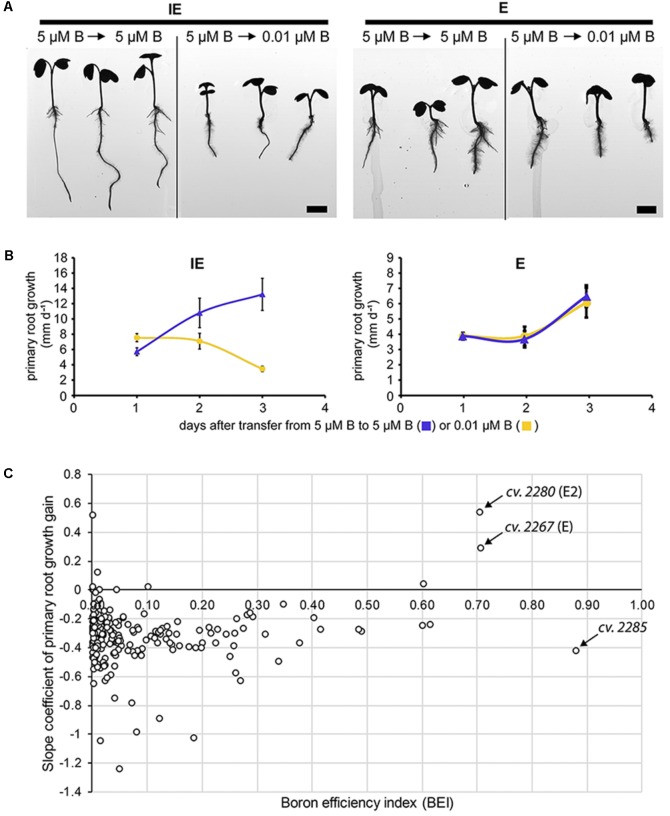
Analysis of B-dependent *Brassica napus* root growth. **(A)** Root cessation assays of the identified B-efficient (E = *CR2267*) and B-inefficient (IE = *CR2262*) *B. napus* cultivars. Plants were transferred from B-sufficient (= control = 5 μM B) to control or B-deficient ( = 0.01 μM B) conditions. IE-, but not E-plants showed growth arrest of root and shoot tissues after transfer to B-deficient conditions. **(B)** Primary root (PR) growth depth per day was recorded for all cultivars and acceleration curves plotted. Acceleration curves for the IE (*CR2262*) and the E (*CR2267*) cultivar after transfer to control or B-deficient conditions are shown. Values represent the average ± SD of three replicates with five plants each (*n* = 15). **(C)** Slope coefficients calculated from the acceleration curves of 229 spring-type *B. napus* accessions plotted against their BEIs. *CR2267* (E) and *CR2280* (E2) exhibited both BEIs greater 0.7 and a positive slope coefficient of PR growth gain. Cultivar *CR2285* with the highest BEI of all tested accessions exhibited a negative slope coefficient. Slope coefficient values of all spring-type rapeseed cultivars are listed in **Supplementary Data Sheet S1**.

In earlier studies, squash (*Cucurbita pepo*), sunflower (*Helianthus annuus*), and Arabidopsis seedlings transferred from B-sufficient to B-deficient medium respond with a growth arrest of the PR within hours (Cohen and Lepper, 1977; Hirsch and Torrey, 1980; Abreu et al., 2014). The roots primarily sense B-deficient soil conditions, and detrimental B deficiency symptoms appear very fast at the root level. We elucidated, whether rapeseed accessions, which have been determined to be B deficiency-tolerant, based on shoot growth parameters at the seedling stage, would display also B deficiency-tolerant traits with respect to root growth parameters. Therefore, we tested, whether the transfer of rapeseed plants to altered B concentrations would result in a similar cessation of root growth. To this aim, seeds from the IPK Genebank spring-type rapeseed collection were germinated on modified Hoagland agar medium with sufficient B levels (5 μM B) and subsequently transferred to new agar plates with either sufficient (5 μM B) or deficient (0.01 μM B) B concentrations. PR elongation per day after transfer was recorded and quantified. Consistently with the results obtained in other species, seedlings from almost all cultivars decelerated the growth of their roots after transfer from B-sufficient to deficient conditions within a day and ceased it completely after 3 more days (**Figure 6**). To visualize root growth as a function over time, we recorded acceleration curves of the root elongation after transfer to B-sufficient or B-deficient conditions. For these acceleration curves, slope coefficients of the PR growth gain were calculated and used as a measure for B efficiency of the root growth during the seedling stage. The slope coefficient values calculated for the spring-type accessions can be found in **Supplementary Data Sheet S1**. Seedlings of the E and E2 accessions were the only B-efficient (BEI > 0.7) ones which did not respond with root cessation ( = negative acceleration of root growth) after transfer to B-deficient conditions and, therefore, showed similar kinetics of their acceleration curves on B-sufficient and B-deficient conditions (**Figure 6**). Roots of E and E2 were thicker and shorter in comparison to roots of the IE and IE2 cultivars. This difference in the root morphology was, however, not causative for the observed B efficiency of these cultivars as also other cultivars with similar roots like E and E2 were identified as B inefficient. Moreover, the cultivar *Qingyou10*, which had been described as a B-efficient genotype before (Xu et al., 2002), exhibited a root system morphology similar to the B-inefficient cultivars IE or IE2 identified in this study. Only one additional cultivar (*cv. CR3195*) exhibited also a positive slope coefficient after transfer to deficient B conditions. This accession however had a low BEI (0.10) characterizing it as highly B inefficient regarding shoot development under B-deficient conditions (**Supplementary Data Sheet S1**).

### Detected B Efficiency of Seedlings Does Not Depend on the B Seed Content

**FIGURE 7:**
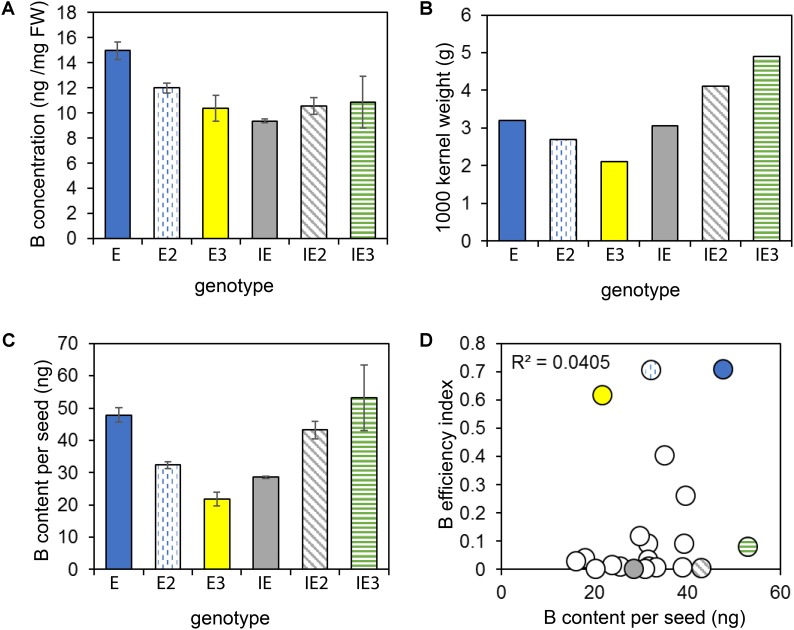
B seed concentrations and contents. B seed concentration **(A)**, 1000-kernel weights **(B)**, and B seed contents **(C)** of selected B-efficient (E = *CR2267*, plain blue; E2 = *CR2280*, vertically dashed blue; E3 = *CR2285*, yellow) and inefficient (IE = *CR2262*, plain gray; IE2 = *CR3153*, diagonally dashed gray; IE3 = *CR3035*, horizontally dashed green) cultivars. **(D)** B efficiency does not correlate with B seed contents. B seed contents of 20 cultivars plotted against their BEIs. Values in **A** and **(C)** represent means from *n* = 3 measurements ± SE.

To test whether differential B accumulation in seeds of the tested accessions were causal for the different B efficiencies of these plants, B concentrations (**Figure 7A**) and contents (**Figure 7C**) were analyzed in mature seeds of the E and IE cultivars as well as in seeds of cultivars of the core collection. The B concentration ranged from 9.4 to 15 ng per mg seed tissue. The B seed contents varied between 21.8 ng B per seed to 53.1 ng B per seed and reflected the relative differences of the 1000 kernel weights (**Figure 7B**). However, the seed B content did not correlate with the calculated BEI among 20 tested accessions (**Figure 7D**). These data indicated that the different B efficiencies recorded for the individual cultivars were not due to differential B storage in the seeds of these accessions.

### Root Growth Responses Toward Various Nutrient Deficiencies of *Brassica napus* Genotypes Contrasting in Their B Deficiency Tolerance

**FIGURE 8:**
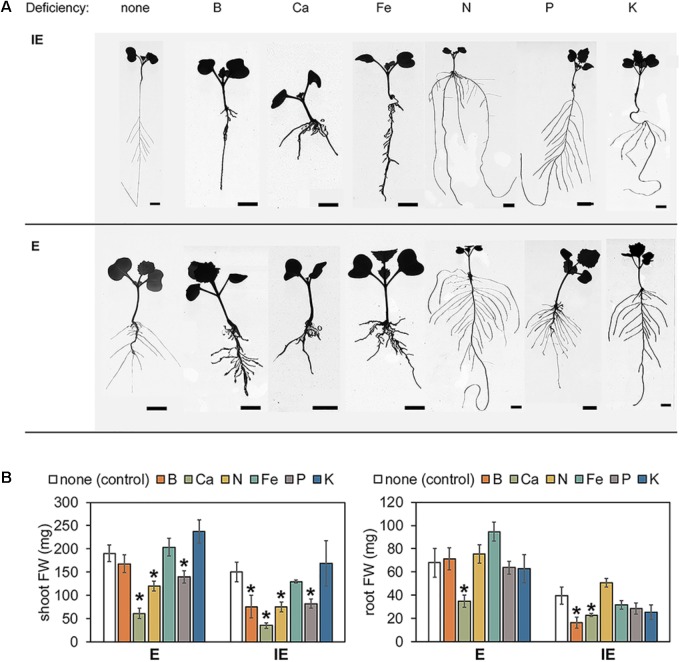
Root system architecture parameters and shoot and root fresh weights (FW) from B-efficient (E = *CR2267*) and B-inefficient (IE = *CR2262*) *Brassica napus* cultivars grown in hydroponic culture medium with different nutrient deficiencies. **(A)** Representative pictures of seedlings grown under indicated nutrient deficiencies for 7–8 days. **(B)** Shoot (left panel) and root (right panel) FW from E and IE plants grown under above conditions. Values represent averages ± SD from *n* ≥ 6 plants. Asterisks indicate significant differences (^∗^*p* < 0.05, *t-test*) of the recorded parameter in comparison to the control (“none”) condition. Bars = 1 cm.

Arabidopsis plants modulate their root system architecture (RSA) quite specifically in response to different nutrient deficiencies including B deficiency (Martín-Rejano et al., 2011; Giehl et al., 2013; Gruber et al., 2013). When grown in hydroponic modified Hoagland solution supplemented with sufficient B levels (5 μM B), plants from the E cultivar exhibited a shorter but thicker PR and a higher total root mass in comparison to the plants from the IE cultivar (**Table 1** and **Figure 8**). When IE plants were transferred from B-sufficient control conditions (5 μM B) to B-deficient conditions (0.01 μM B), they responded with a growth arrest of their lateral roots resulting in a strongly reduced total root mass in comparison to control conditions, as it has already been reported for other plant species (Cohen and Lepper, 1977; Martín-Rejano et al., 2011; Camacho-Cristóbal et al., 2015). Such alterations were under these conditions less pronounced in plants of the E cultivar, underlined by non-significant changes of lateral root number and length resulting in unchanged total root mass in comparison to control conditions (**Table 1** and **Figure 8**). However, similar to IE, the E cultivar displayed significant alterations of its RSA in response to macro- (Ca, N, P, K) and micronutrient (Fe) deficiencies other than B, demonstrating a strong B-specific nutrient efficiency of the E cultivar at the seedling stage (**Table 1** and **Figure 8**). When grown under N-deficient conditions on zerosoil-substrate both cultivars responded with severe stress and N deficiency-related symptoms, indicating that the cultivars did not differ in their N deficiency tolerance (**Supplementary Data Sheet S11**).

**Table 1:** Root system architecture parameters for the IE (*CR2262*) and the E (*CR2267*) *Brassica napus* cultivars grown under different nutrient (B, Ca, Fe, N, P, and K) deficiency- and control conditions.

IE deficiency	PR length (cm)	1° LR number	Total 1° LR length (cm)	Average 1° LR length (cm)	1° LR density (number cm^-1^ PR)
None (control)	7.23 ± 1.61	18.00 ± 4.53	18.86 ± 4.35	1.09 ± 0.32	2.50 ± 0.39
B	5.82 ± 2.07	3.33 ± 1.51^∗^	3.75 ± 0.94^∗^	1.26 ± 0.49	0.48 ± 0.29^∗^
Ca	5.97 ± 0.80	6.80 ± 1.64^∗^	4.60 ± 1.52^∗^	0.66 ± 0.12^∗^	1.17 ± 0.34^∗^
Fe	11.24 ± 3.61	27.60 ± 6.58^∗^	15.68 ± 4.75	0.56 ± 0.08^∗^	2.57 ± 0.54
N	12.40 ± 3.01^∗^	24.00 ± 8.74	41.38 ± 12.27^∗^	1.93 ± 0.86	2.16 ± 1.24
P	11.38 ± 2.67^∗^	18.80 ± 4.27	32.81 ± 4.44^∗^	1.77 ± 0.27^∗^	1.71 ± 0.48^∗^
K	13.81 ± 4.11^∗^	17.17 ± 2.86	27.18 ± 3.53^∗^	1.62 ± 0.36^∗^	1.33 ± 0.39^∗^
					
E deficiency					
None (control)	6.49 ± 1.97	16.80 ± 2.28	20.69 ± 7.79	1.24 ± 0.42	2.83 ± 1.06
B	5.23 ± 0.98	14.17 ± 1.17	14.47 ± 5.52	1.01 ± 0.33	2.79 ± 0.58
Ca	2.01 ± 0.28^∗^	15.50 ± 2.59	5.52 ± 1.92^∗^	0.35 ± 0.10^∗^	7.88 ± 1.77^∗^
Fe	6.24 ± 0.38	25.33 ± 2.94^∗^	21.77 ± 7.00	0.87 ± 0.27	4.06 ± 0.38^∗^
N	17.38 ± 6.58^∗^	32.40 ± 4.72^∗^	71.00 ± 9.96^∗^	2.21 ± 0.30^∗^	2.04 ± 0.73
P	8.65 ± 4.01	26.60 ± 6.58^∗^	44.79 ± 2.71^∗^	1.76 ± 0.37^∗^	3.39 ± 1.29
K	8.60 ± 3.04	31.20 ± 3.19^∗^	43.81 ± 6.58^∗^	1.41 ± 0.23	3.83 ± 3.83


*Values are means from five to six replicates ± SD. Asterisks mark significant differences to control conditions (*t*-test, ^∗^*P* < 0.05). (PR, primary root; LR, lateral root; 1°, first order). The experiment has been repeated independently with similar results.*

### Responses Toward Toxic B Conditions of *Brassica napus* Genotypes Contrasting in Their B-Deficiency Tolerance

**FIGURE 9:**
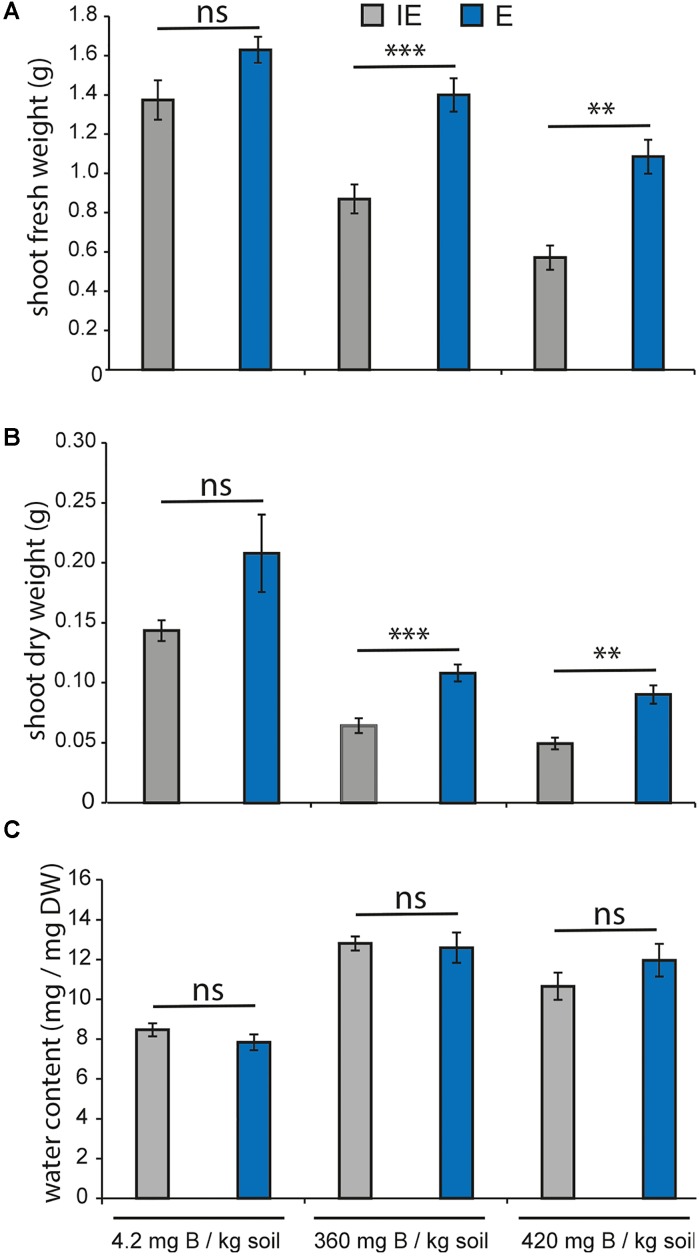
Boron toxicity growth assays of B-efficient (E = *CR2267*, blue chart bars) and B-inefficient (IE = *CR2262*, gray chart bars) *B. napus* cultivars on zerosoil-substrate with toxic B levels. Values represent averages ± SE from shoot material (*n* ≥ 17). Asterisks indicate significant differences (^∗∗^*p* < 0.01, ^∗∗∗^*p* < 0.001, *t-test*) of the recorded parameter between the two cultivars in the same growth condition (Ns = not significant). **(A)** Shoot FW in grams, **(B)** shoot DW in grams, and **(C)** water content in % are displayed.

Next, we tested whether the two contrasting cultivars, E and IE, would show differential tolerance to toxic B soil concentration. To this aim, plants from both accessions were grown on 100-fold higher B concentrations than the control conditions and shoot FW and DW at 25 DAG were measured (**Figures 9A,B**). FW of both cultivars decreased between 35 and 50% on the high B concentrations in comparison to the control condition (**Figure 9A**), and DW (**Figure 9B**) decreased more than 75% on the high B concentration. The soil B-dependent loss of both FW and DW was significantly greater in the IE than in the E cultivar indicating a higher tolerance also to toxic soil B conditions of the E cultivar. The water content of plants grown on the high B concentrations was higher than that of plants grown under control conditions, suggesting that both, E and IE, responded with increased water uptake to the excess B inflow (**Figure 9C**).

### Developmental- and B-Dependent B Accumulation in *Brassica napus* Genotypes Contrasting in Their B-Deficiency Tolerance

**FIGURE 10:**
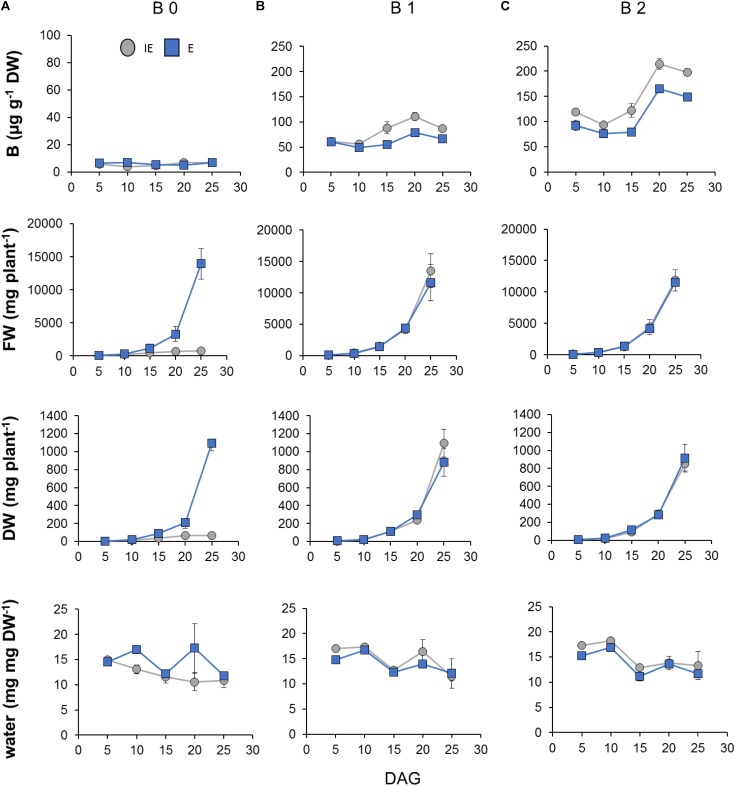
Boron (B)- and cultivar-dependent B accumulation and growth-reflecting parameters during early vegetative development (until 25 days after germination; DAG) of *B. napus* plants from B-inefficient (IE = *CR2262*, gray dots) and B-efficient (E = *CR2267*, blue squares) cultivars growing on B-deficient (B 0), B-adequate (B 1), or B-surplus (B 2) zerosoil-substrate conditions. **(A)** Shoot FW in mg per plant, **(B)** shoot DW in mg per plant, **(C)** water content in %, and shoot B concentrations are displayed. Measurements are means ± SD from *n* = 4 biological replicates containing each six to eight plants.

For a more detailed developmental-dependent analysis of the B-dependent response of the B-deficiency tolerant and sensitive cultivars during early vegetative development, both cultivars had been germinated on B 0 or B 1 zerosoil-substrate conditions and additionally on surplus B supply [B2 = 12.8 mg B (kg zerosoil-substrate)^-1^] and were then grown until 25 DAG (**Supplementary Data Sheets S2A,B**). At 5, 10, 15, 20, and 25 DAG the fresh and DWs, the water contents, and the shoot B concentrations have been determined. The differential B supply levels resulted in significantly altered fresh and DWs of the IE, but not the E cultivar (**Figures 10A,B**). Plant water contents, however, were similar between cultivars and B supply levels (**Figure 10C**). Interestingly, at these young developmental stages, IE accumulated B to higher concentrations than E under B 1 and B 2 conditions indicating a higher B demand for IE in comparison to E (**Figure 10D**). Under the low B 0 condition, however, both cultivars accumulated 10- (E) to 12-fold (IE) less B than under B 1, and did not differ significantly in their absolute B concentration over the analyzed time. These results show that the E cultivar could develop normally under low internal B concentrations, which clearly impaired growth of the IE cultivar (**Figure 10**).

**FIGURE 11:**
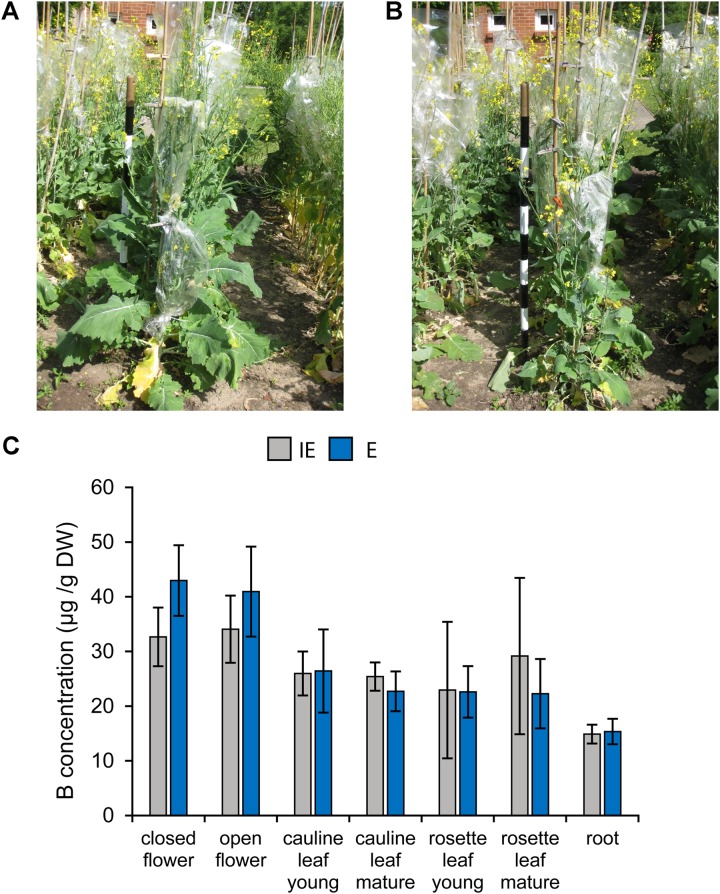
Growth of B deficiency tolerant and sensitive *B. napus* cultivars under near-field conditions with sufficient B supply (0.39 mg B/kg soil; hot water extract). Exemplary pictures of flowering plants of the IE (*CR2262*) **(A)** or E (*CR2267*) **(B)** cultivars grown under near-field conditions. **(C)** B concentrations of indicated plant tissues from IE (gray chart bars) and E (blue chart bars) cultivars grown under near-field conditions.

Both cultivars did not exhibit obvious morphological differences when grown with suboptimal, but adequate B supply [at 0.39 and 0.44 mg B (kg soil)^-1^] under near-field conditions at two different locations in Poel, Germany (**Figures 11A,B**). In addition, no significant differences in the B concentrations were detected between the same plant tissues of the different cultivars grown under near-field conditions. Aerial plant parts, namely leaves and inflorescences of both cultivars had twofold to threefold higher B concentrations, respectively, than root tissue indicating efficient B-translocation into shoots. This is in accordance with an increased B demand of leaves and flowers, compared to roots (**Figure 11C**).

## Discussion

### A Newly Established Soil Cultivation System Allows Resolution of B-Dependent Growth Effects

In this study, we identified rapeseed cultivars with increased tolerance toward B-deficient conditions. The growth behavior in B-deficient conditions was recorded in different cultivation systems, namely in soil-grown plants, in plants grown in agar medium, and in plants grown in hydroponic culture medium. The identification of these cultivars demanded to work with defined and repeatable B nutrient regimes. This is challenging because B is a trace element with a very small range between deficient, sufficient, and toxic conditions and a high mobility in various soils. Plant-available B can vary tremendously even in locally restricted field plots. To circumvent the heterogeneous B availability in soils, very often, hydroponic growth systems are chosen in which B supply can be adequately adjusted, e.g., by the use of B chelating resins (Asad et al., 1997; Stangoulis et al., 2000; Savić, 2013). However, soil substrates are superior to hydroponic or pure sand cultivation systems as they enable the rhizosphere to develop in a way similar to natural conditions. This is of importance, as parameters shaping the rhizosphere and subsequently the rhizosphere itself have a particularly high impact on the uptake of water and nutrients and therewith on plant growth traits (Gregory, 2006; Oburger and Schmidt, 2015). In the present study a novel soil substrate-based soil column cultivation system was developed for assessment of plant growth in an automated high-throughput phenotyping facility under highly defined soil B conditions. Because of the high variability of B-bioavailability in most soils, we pre-screened soil substrates and chose one with B concentrations below 0.1 mg B (kg soil)^-1^ (=B-deficient). The successful identification of B deficiency-tolerant rapeseed cultivars demonstrated that this substrate allowed to set and control the nutrient status of plants in a highly defined manner. Moreover, the herein developed and established procedures and protocols using this soil substrate will allow performing successful micronutrient efficiency screenings of *Brassica* crops in automated high-throughput phenotyping facilities which is of great relevance and timely. The system can in the future also be adapted to other plant species.

### Screening of Independent *B. napus* Collections Identified B-Efficient and B-Inefficient Cultivars

In the 1990s, 210 cultivars from the germplasm collections of the Genetic Breeding Institute of Rapeseed at Huazhong Agricultural University and the Oil Crop Research Institute of the Agricultural Academy of China have been screened for B-efficient *B. napus* accessions. This screening led to identification and characterization of the B-efficient *cv. Qingyou10* (Wang and Lan, 1995; Wang et al., 1996; Xu et al., 2002). In the present survey, we screened 234 spring- and 356 winter-type *B. napus* cultivars from the Genebank of the IPK Gatersleben for various B deficiency tolerance growth traits in plants grown until around BBCH15 on B-sufficient and B-deficient conditions (**Supplementary Data Sheet S1**). All 590 assayed accessions have been indexed for their B-deficiency tolerance growth behavior. We included all spring-type cultivars of the IPK Genebank as well as winter-type cultivars incorporating a representative collection from all over the world. We chose mainly winter-type cultivars belonging to the “++”-type being assumed to represent most of the genepool in which modern traits such as low erucic acid and glucosinolate levels or Phoma resistance have been introgressed. We also included *cv. Bronowski* (*CR0270*) and *cv. Liho* (*CR0704*) in our screen. *Bronowski* was the first cultivar identified to possess very low-glucosinolate levels and a spontaneous mutant of *Liho* was identified to be free of erucic acid. Both these spring-type traits have been subsequently introgressed into our-days commonly used spring- and winter-type cultivars. *Bronowski* and *Liho* are B deficiency-sensitive (see BEI in **Supplementary Data Sheet S1**). This suggests that the trait “B deficiency tolerance” was not introduced into modern elite lines during this bottleneck breeding phase. Moreover, we included *B. napus* cultivars which have been described in the literature to be B-efficient, moderate B-efficient, or B-inefficient as reference material to demonstrate the power and validity of our screening conditions. *Zhongshuang11* and *Qingyou10*, which were described as B-efficient cultivars in the past (Xu et al., 2001, 2002), were not ranked as B-deficiency-tolerant in our screening conditions (see **Supplementary Data Sheet S1**), due to the fact that we defined a more stringent BEI threshold value for a B deficiency tolerance assignment within our *B. napus* accession panel. Additionally, we employed harsher B-deficient conditions in our screen [<0.1 mg vs. 0.25 mg B (kg soil)^-1^] than those applied by Wang and co-workers (Wang and Lan, 1995; Wang et al., 1996). As a consequence, *cv. Ningyou7* (*CR0796*), another cultivar which has been described in the literature to be moderately B-efficient (Zhao et al., 2008), was classified as B-deficiency-sensitive by us according to its growth parameter scores. A variety of cultivars described as B deficiency-sensitive before, including *Westar* (*CR1054*) or *Campino* (*CR3430*) (Zhang et al., 2014a; Eggert and von Wirén, 2017) did also rank as highly B deficiency-sensitive in our study. Together, these results highlight the power and validity of our screening conditions and scoring method.

### An Automated Phenotyping Approach Revealed Informative Parameters of the B Nutritional Status

In this study, visual light and fluorescence imaging modules have been employed in high-throughput plant phenotyping analyses to assess morphological and physiological plant traits. Although also the application of hyperspectral imaging modules is increasingly tested for the evaluation of *in vivo* plant vigor and the plant nutrient status, this technique clearly failed in the quantification and prediction of the plant nutritional B status during high-throughput analyses of crop plants despite being successful for other tested macro- and micronutrients (Pandey et al., 2017). Besides B, the only other element which could not be quantified by hyperspectral imaging was Na (Pandey et al., 2017). In our study, we demonstrate that in high-throughput phenotyping approaches the evaluation of biomass and growth parameters by visual light and fluorescence imaging modules or by physical parameter determination are the methods of choice to reliably assess and identify B deficiency-tolerant genotypes. Quantified color-related imaging parameters tended to separate cultivars with low tolerance from those with high tolerance to B deficiency but not the different B treatments. Their relevance for the detection of B deficiency tolerant cultivars will be subject of further detailed investigations.

Moreover, the results demonstrate that traits which contribute to B efficiency may appear at different developmental stages. Therefore, for identification of B-deficiency-related traits, recording of growth dynamics seems preferable to one time-point examination in particular when studying underlying genetic and physiological mechanisms. Our high-resolution analyses of the growth rate inhibition of IE and IE2 genotypes in response to B deficiency (**Figure 3**) clearly demonstrated that modern high-throughput phenotyping facilities and subsequent image analysis can provide such advantages. Such a high-resolution-knowledge is of great relevance for the identification of specific B deficiency tolerance traits but also for the implementation of an efficient agricultural fertilization management. Early developmental stages are relevant growth phases for the identification of B-efficient genotypes/accessions as vegetative responses to B deficiency of young *B. napus* plants grown in soil column experiments reflected also their B efficiency performance over the entire growth period, also under field conditions, as suggested and demonstrated by other studies (Chu et al., 1999; Stangoulis et al., 2000). Investigations at early growth stages can advantageously be performed under highly controlled conditions (with rather small plant bodies) and reduce the impact of secondary biotic and/or abiotic effects occurring during the long growth period of rapeseed. The high-resolution growth analysis is, at these early growth stages, more informative for impact and severity of B-deficient conditions than other color-related traits (e.g., darkening of leaves, accumulation of anthocyanins) since latter start only to appear at later growth stages after the establishment of the shoot growth arrest and the onset of other severe and irreversible B deficiency symptoms.

### Comparative Characterization of *B. napus* Cultivars With Contrasting B Efficiencies

In this work three highly B deficiency-tolerant Asian spring-type *B. napus* cultivars were identified and characterized. One of these cultivars named “*Hiyauchina*” = *cv. CR2267* (herein termed as “E”) was subsequently analyzed in more detail. *CR2267* developed large leaves and exhibited late flowering, typical characteristics for leafy or forage but not oilseed *B. napus* cultivars (Soengas et al., 2008). According to the IPK’s Genebank documentation, this accession had been collected at the island of Taiwan in 1988. Interestingly, all in this study identified accessions with high BEIs originated from eastern Asiatic countries. Main cultivable acreages of these countries suffer from low B conditions, e.g., granite derived soils in Korea [0.07–0.15 mg hot-water soluble B (kg soil)^-1^] and large parts of China [0.25 mg hot-water soluble B (kg soil)^-1^] (Shorrocks, 1997; Yan et al., 2006). This might suggest that certain *B. napus* cultivars succeeded in evolving B deficiency tolerance mechanisms as an adaptation to the limited B availability at these agricultural sites. Despite its susceptibility for B-deficient conditions, *B. napus* is one of the major oil crops in China (Hu et al., 2017) reaching 16.8 million tons in 2016/2017 (USDA^5^) and Chinese groups already succeeded in the isolation and crossbreeding of B-efficient genotypes (Hu et al., 1991; Xu et al., 2002; Shi et al., 2009). The cultivars identified in this study have not yet been described with respect to their B deficiency tolerance and are different to the already published B-efficient accessions (Xu et al., 2002; Yang et al., 2013). These accessions are, therefore, a highly valuable genetic resource for the search of genomic B efficiency loci and molecular and physiological B efficiency traits. The results from our implemented screening demonstrated that B efficiency is a very rare trait in *B. napus*, since only 3 out of 590 analyzed accessions exhibited clear B-efficient growth and development (**Figure 1** and **Supplementary Data Sheet S1**). Moreover, the B deficiency tolerance trait seem to be predominantly encoded in spring-type cultivars, as no highly B-efficient winter-type cultivar has been identified yet.

For comparison of physiological and nutritional responses, among the many B-inefficient accessions, we selected B-inefficient cultivars with similar morphologic properties under adequate B conditions compared to the E cultivar. For instance, *cv. CR2262* (=IE), exhibited DW, hypocotyl length, and leaf length values under adequate B conditions like *cv. CR2267* (=E). Plants of *CR2262* had large leaves and a late onset of flowering, which was indicative that also this genotype is a leafy or fodder rapeseed cultivar. However, no information existed, whether the cultivar was indeed a leafy or fodder rapeseeds. *CR2262* had been collected 1981, 4 km Northeast of Pago Veiano, in the province Benevento, Campania, Italy.

### B-Dependent Sugar Accumulation in Contrasting Cultivars

Boron deficiency disrupts vascular tissue differentiation and integrity which results in irreparable damage of the affected transport routes. These impairments have detrimental and irreversible consequences on long distance transport processes and often generate transport bottlenecks, which are of particular concern when occurring early in the development or in minor leaf veins (Letho et al., 2010; Wimmer and Eichert, 2013). Our analysis of soluble sugars in cotyledons of IE and E plants cultivated in the high-throughput phenotyping facility indicated such B-dependent impairment of sugar transport and accumulation (**Figure 4**). Both glucose and sucrose levels showed a drastic increase under B-deficient zerosoil-substrate conditions in comparison to adequate B conditions. Especially, the high sucrose levels indicated impaired phloem loading and transport. Interestingly, surplus fertilization of the soil substrate with B [as in the (++) B condition] led also to an increase of monosaccharides glucose and fructose, but not to an accumulation of sucrose, indicating that vascular transport was unaffected in plants grown under high B soil conditions, but carbon assimilation was elevated. The results from the DW measurements (**Figure 3**) supported a scenario, in which plants of at least E used such elevated availability of building blocks for the development of biomass. In this case, the elevated B soil conditions had beneficial effects on shoot growth but no detrimental consequences as it would appear under toxic B conditions. High levels of soluble sugars in the cytosol of source leaves normally feedback negatively on both photosynthetic activity and expression of genes related to CO_2_ fixation (Rolland et al., 2002; Eveland and Jackson, 2011). The perception of constantly elevated cytosolic sugar levels represents an emergency signal for the plant metabolism reflecting growth arrest and persistently elevated stress levels (Rolland et al., 2002). B-efficient genotypes, however, apparently had the ability to keep soluble sugars low by incorporation of sugars into biomass and proceeding growth and development.

### Boron and Nutrient Efficiency in Contrasting Cultivars

Boron concentrations in aerial tissues of the E cultivar under B 0 conditions were extremely low and not exceeding the B concentration of IE tissues, clearly excluding the possibility that B contamination in the test soil substrate was causative for the superior growth and the higher B efficiency of the E in comparison to the IE cultivar. Seed B level quantifications further showed that B deficiency tolerance and vigorous seedling growth under B-deficient conditions does not correlate or depend on the seed B content. This is in agreement with earlier studies (Eggert and von Wirén, 2015). Under B1 and B2 conditions in the greenhouse, both cultivars accumulated significant amounts of B in a soil B concentration-dependent manner (**Figures 1**, **11**). Under these conditions, the leaf B concentration of plants from the IE cultivar was roughly 10–20% higher than that of the E cultivar indicating an elevated B demand or uptake of the IE cultivar. The same observations applied to plants grown in the phenotyping facility. One may speculate that the different physiological and nutritional B-dependent responses of the different B-efficient cultivars (E and E2) were due to different B-related QTLs. In the same context, it can be argued that also different sensitivities toward B deficiency were caused by different underlying genetic mechanisms. The results from the analysis of these cultivars under B-deficient conditions in the automated plant phenotyping facility supported such a scenario since the calculated RGRs revealed cultivar-dependent differences, in both B deficiency-sensitive and -tolerant accessions (**Figure 3**). These results further indicate that the B-inefficient cultivars differ in susceptibility to B-deficiency depending on specific developmental time points. Whereas the IE cultivar apparently responded with a growth arrest immediately after the outgrowth of the cotyledons, plants from the D and IE2 cultivars were able to expand their cotyledons but failed to develop successive vegetative leaves. This marks the emergence of the first vegetative leaf as a critical developmental time point for the timely allocation of B in the shoot apical meristem.

However, leaves of adult plants with fully developed inflorescences grown under near-field conditions did not exhibit a genotype-dependent B leaf accumulation (**Figure 11**). Interestingly, flower B concentrations of such plants clearly exceeded those of leaves and roots and were slightly higher in the E than in the IE cultivar demonstrating that inflorescences are strong B sinks and may suggest that plants of the E cultivar allocate B more efficiently into these sinks than plants of the IE cultivar. A high B requirement of generative organs is general to all seed plant species, also those which have a very low B demand during vegetative plant growth such as cereals (Marschner, 2012). Under B-deficient soil conditions or due to a lack of active B transport proteins, plants fail to develop fertile flowers and seeds (Noguchi et al., 1997; Rerkasem and Jamjod, 2004; Marschner, 2012). Crop plant disorders such as “flowering without seed setting” and “budding without flowering” in oilseed rape and cotton, respectively, which were occurring in Chinese agricultural areas had been discovered to be caused by B deficiency (Wang et al., 2007). One possible explanation for an increased B deficiency tolerance phenotype under B limiting conditions is an enhanced B uptake and translocation ability of the cultivar, as it was demonstrated for *cv. Qingyou10* (Hua et al., 2016; Zhang et al., 2017). While transport kinetics of the contrasting cultivars, E, E2, IE, and IE2, will have to be determined in future, the similarly low B tissue concentrations of these cultivars under B deficient conditions argue against the fact that B uptake traits are solely responsible for the B deficiency tolerance. Moreover, the results which have been obtained under toxic B growth conditions showed that the E cultivar was also less sensitive to toxic B concentrations (**Figure 9**) suggesting that E can fine-regulate its B transporters according to the B status of the growth medium and that the B deficiency tolerance is not due to a permanently active B uptake system. Otherwise E should be more sensitive to toxic B conditions. Together our results suggest that the herein identified B deficiency tolerance traits seem to be based on B utilization mechanisms and not solely on B transport-related traits. A combination of the different B efficiency mechanisms by crossing the independently identified B deficiency tolerant *B. napus* genotypes (e.g., *CR2267* and *Qingyou10*) will provide the possibility to succeed in a trait stacking of plant B nutritionally valuable traits within one genotype. This would be highly desirable given that plants with united beneficial traits can be used as pre-breeding material to generate highly B-efficient rapeseed cultivars suitable for a sustainable agricultural usage in B-deficient areas.

The specific modulation of a plant’s RSA is exemplary of the impact of a specific nutrient deficiency (Giehl et al., 2013; Gruber et al., 2013). Both the E and the IE cultivar responded with significant and typical changes of several RSA parameters and with changes in root and shoot FWs to Ca, Fe, N, P, and K deficiencies (**Table 1** and **Figure 7**). In detail, both cultivars showed a dramatic increase in the total first order LR length to N, P, and K deficiency indicative for a typical response reaction toward these nutrient deficiencies. These results indicated that the E cultivar did not possess any elevated general nutrient efficiency due to an intrinsic different RSA or other root traits. Interestingly, cultivars which have been identified as B-deficiency-tolerant based on shoot growth parameters displayed also lower root growth inhibition under low B conditions both in hydroponics and in *in vitro* agar-based medium. This observation strongly suggests that both root and shoot traits contribute to their resilience under B-deficient conditions. The underlying mechanisms remain to be identified. The non-differing low B concentrations in shoots of E and IE plants (**Figures 1**, **10**) grown at B-deficient soil conditions, however, indicate that the B-independent root and shoot growth ability of E plants is most likely not solely related to an increased B uptake affinity of B transport mechanisms, but may rather hint toward a cell expansion- and tissue growth ability in the presence of trace amounts of B, possibly maintained by an altered cell wall polysaccharide composition or B-independent growth signaling transductions. To elucidate, whether such underlying mechanisms are eventually mediated by altered B-dependent signaling cascades or phytohormone responses will be issue of follow-up studies.

### Future Perspectives of B-Deficiency Tolerant Varieties

Rapeseed has been cultivated worldwide in different climatic and environmental regions as well as on B-deficient and B-sufficient soils for a long period of time. Spring-, semi-winter-, and winter-type cultivars have been developed as well as oil-, fodder-, and cover crop varieties. Therefore, it is likely that different B deficiency tolerance mechanisms exist in this species. It has been hypothesized that the B uptake capacity, the re-translocation rate to sink tissues, the B utilization ability, as well as the ROS protection capacity contribute to B efficiency in different plant species including *B. napus* (Geng et al., 1999; Nachiangmai et al., 2004; Zeng et al., 2007; Zhang et al., 2014a; Hua et al., 2016). Conventional, biotechnological, and Selection with Markers and Advanced Reproductive Technologies (SMART) breeding strategies benefit from the identification of B-efficient *B. napus* genotypes. In future, the cultivars identified in this study will be investigated in further detail at the transcriptomic level in response to different B treatments, with respect to their B uptake and translocation abilities, their cell wall composition, their B-dependent signaling cascades and in terms of differing quantitative trait loci in order to unravel underlying mechanisms and genetic determinants of B efficiency.

## Conclusion

In summary, we succeeded to develop a reliable soil-substrate-based cultivation system which is compatible with high-throughput phenotyping facilities and which allows, in the first instance, identifying B deficiency tolerant cultivars as well as development-dependent B-sensitive growth stages and subsequently, characterizing B-dependent and cultivar-specific growth characteristics in detail. In accordance with other reports (Pandey et al., 2017), our study suggests that biomass-related traits are superior to color-related imaging traits when assessing B-efficient growth of young plants. Comparison of imaging-based and physical property-based biomass quantification validated the herein used experimental set-up and analysis pipeline and highlights the potential of this set-up in the future identification of further B-efficient genotypes, also in other species. Using different phenotyping technologies and approaches, we screened IPK Genebank rapeseed cultivars for B deficiency tolerance. We identified three highly B deficiency tolerant Asian spring-type cultivars, which grow phenotypically normal on 0.1 mg B (kg soil)^-1^ until flowering, while others stopped their growth at the cotyledon stage. This indicates that the trait “B deficiency tolerance” is very rare in *B. napus* and probably evolved as an adaption to the cultivation on agricultural soils with low B levels. Elemental analyses revealed significant differences in B contents of B deficiency tolerant- and sensitive cultivars when grown under B-deficient but not under standard conditions. These results indicate that the herein identified cultivars have a very low demand for B and an increased B utilization efficiency as they can grow with a very limited amount of this micronutrient. In contrast, so far identified cultivars which are more tolerant to B limiting conditions inhere in beneficial B uptake and translocation traits. Moreover, we can conclude that the nutrient deficiency tolerance of the herein identified cultivars is restricted to B-deficient conditions and is not subjected to a general growth vigor at the seedling stage. Comparisons of root and shoot growth describing B deficiency tolerance indices of plants grown under B-deficient and -sufficient conditions indicated that the used *B. napus* genotype panel encodes for both root- and shoot-based traits which can independently or synergistically contribute to B deficiency tolerance. Our collection of genetically diverse *B. napus* genotypes set the basis for successive genetic, molecular, phenotypic, and metabolic analyses to identify shoot- and root-based B efficiency mechanisms as well as their underlying responsible loci/genes in *B. napus*. The identified B deficiency tolerant cultivars will potentially allow to introduce the B utilization-related B deficiency tolerance traits alone, or by trait stacking, in combination with previously identified B-uptake and translocation traits, into modern elite lines to generate highly nutrient- and resource-efficient genotypes in a sustainable manner in the future.

## Author Contributions

All authors prepared, read, and approved the final manuscript. BP and GB conceived the study and wrote the paper with the input of all authors. EW performed the plant cultivation experiment in near-field conditions. B-toxicity assays have been performed by MB. The B deficiency tolerance screen, the root cession assay, growth assays, and related experiments were conducted mainly by AB, JF, BP, and GB but with the help of other authors. Work related to the automated imaging/phenotyping and subsequent analyses have been performed by AJ, IA, BP, TA, and GB. Characterization of seeds was done by BP. BP, GB, MB, TA, JF, AB, AJ, and IA conducted experiments and analyzed the data.

## Conflict of Interest Statement

The authors declare that the research was conducted in the absence of any commercial or financial relationships that could be construed as a potential conflict of interest.

## Footnotes

1 www.fao.org

2 https://gbis.ipk-gatersleben.de/gbis2i/

3 https://gbis.ipk-gatersleben.de/gbis2i/

4 http://www.ipk-gatersleben.de/en/dept-genebank/satellite-collections-north/

5 http://apps.fas.usda.gov/psdonline/
