# Supplementary material for: Identification of Rapeseed (Brassica napus) Cultivars With a High Tolerance to Boron-Deficient Conditions
Source: Front Plant Sci. 2018 Aug 7;9:1142. doi: 10.3389/fpls.2018.01142 (PMC6091279; doi:10.3389/fpls.2018.01142)
Supplement: Supplementary file 1 [file Data_Sheet_1.docx]

**Supplementary_Data_Sheet_S1:** List of *Brassica napus* genotypes which have been used for the B-deficiency tolerance screen. 234 spring-type and 356 winter-type are listed and named by their IPK Genebank Accession code. The designation to the *B. napus* core collection described by Lühs *et al.* (2003) is indexed with a ‘c’ following the accession code. Moreover, the life form, the country-origin, the dry weight (DW) in mg and the length of leaf 1 (L1) (in mm) under B1 (2.4 mg B (kg soil)^-1^) and B0 (<0.1 mg B (kg soil)^-1^) growth conditions and the calculated ‘Boron Efficiency Index’ is listed. The bigger the value the higher the B-deficiency tolerance of the genotype. Additionally, the ‘Slope coefficient’ of primary rot growth gain, which was determined in *in vitro* growth transfer assays is displayed for the genotypes which have been evaluated for their root growth behavior after the transfer to B-deficient (0.01 µM boric acid) or B-sufficient (5 µM boric acid) conditions.

| **Genotype - Accession Code** | **Life form** | **Origin** | **DW B1 (mg)** | **DW B0 (mg)** | **L1 B1 (mm)** | **L1 B0 (mm)** | **Boron Efficiency Index** | **Slope Coefficient of Primary Root Growth Gain** |
| --- | --- | --- | --- | --- | --- | --- | --- | --- |
| CR 3126 | winter-type | Germany | 207,2727273 | 180,1 | 53,33333333 | 34,83333333 | 0,567502604 | - |
| CR 3019 | winter-type | Italy | 234,8571429 | 202,4545455 | 42,54545455 | 30,91666667 | 0,626416595 | - |
| CR 3219 | winter-type | Austria | 234,8571429 | 202,4545455 | 42,54545455 | 30,91666667 | 0,626416595 | - |
| CR 3061 | winter-type | Germany | 308,75 | 214,3333333 | 54,91666667 | 27,58333333 | 0,34867863 | - |
| CR 3218 | winter-type | Netherlands | 258,5083333 | 173,8 | 46,33333333 | 24,45454545 | 0,354847111 | - |
| CR 0320 | winter-type | Netherlands | 44,75 | 29,66666667 | 39,75 | 16,33333333 | 0,272403952 | - |
| CR 3286 | winter-type | Germany | 222,125 | 145,7777778 | 48,875 | 31,44444444 | 0,422231899 | - |
| CR 0813 | winter-type | Italy | 247,2727273 | 156,8333333 | 46,58333333 | 21,66666667 | 0,29500114 | - |
| CR 0169 | winter-type | Sweden | 281,8166667 | 178,4333333 | 42,5 | 32,58333333 | 0,485418022 | - |
| CR 3313 | winter-type | Germany | 419,5833333 | 247,1666667 | 78,16666667 | 36,08333333 | 0,271929754 | - |
| CR 0170 | winter-type | France | 250,25 | 146,3333333 | 40,5 | 17,41666667 | 0,251465955 | - |
| CR 3223 | winter-type | Germany | 384,9166667 | 224,1666667 | 52,58333333 | 28,75 | 0,318415392 | - |
| CR 0323 | winter-type | x | 615,9166667 | 351,0833333 | 67,58333333 | 29,91666667 | 0,252325912 | - |
| CR 3075 | winter-type | Germany | 203,375 | 111 | 49,45454545 | 16 | 0,176579052 | - |
| CR 0689 c | winter-type | Germany | 393,8333333 | 213,9166667 | 69 | 34,75 | 0,273550725 | - |
| CR 0332 | winter-type | Germany | 196,6666667 | 106 | 53,5 | 14,16666667 | 0,142721369 | - |
| CR 1106 | winter-type | Sweden | 292,0916667 | 157,25 | 47,33333333 | 27,33333333 | 0,310883015 | - |
| CR 3311 | winter-type | Germany | 205,5 | 110,5833333 | 48,41666667 | 18,16666667 | 0,201910178 | - |
| CR 0758 | winter-type | x | 418,75 | 220 | 57,41666667 | 32,25 | 0,295093473 | - |
| CR 2194 | winter-type | Germany | 250 | 129 | 59,25 | 23 | 0,200303797 | - |
| CR 1036 | winter-type | Sweden | 288,1333333 | 145,7 | 49,16666667 | 23,54545455 | 0,242159974 | - |
| CR 3293 | winter-type | Germany | 260,5 | 130,8333333 | 47,41666667 | 14,66666667 | 0,155349937 | - |
| CR 0853 c | winter-type | Poland | 321 | 156,65 | 62,08333333 | 22 | 0,172931067 | - |
| CR 1057 | winter-type | Netherlands | 283,3833333 | 133,4583333 | 44,08333333 | 21,58333333 | 0,230576735 | - |
| CR 1108 | winter-type | Sweden | 343,3666667 | 161,4083333 | 47,16666667 | 27,75 | 0,276563984 | - |
| CR 3130 | winter-type | Germany | 314,5 | 147,3333333 | 66,16666667 | 21,16666667 | 0,149862709 | - |
| CR 3287 | winter-type | Germany | 398,5 | 180,5454545 | 69,91666667 | 22,63636364 | 0,146684485 | - |
| CR 3054 | winter-type | Germany | 364 | 156,3333333 | 52,66666667 | 14,33333333 | 0,116885751 | - |
| CR 3137 | winter-type | Netherlands | 275,4916667 | 115,85 | 47 | 19,08333333 | 0,17074341 | - |
| CR 3113 | winter-type | Netherlands | 534,6 | 223,6916667 | 37,27272727 | 24,41666667 | 0,274104429 | - |
| CR 0193 | winter-type | Netherlands | 278,9166667 | 115,2 | 44,33333333 | 18,2 | 0,169558285 | - |
| CR 3168 | winter-type | Germany | 262,75 | 104,6666667 | 53,33333333 | 17,33333333 | 0,129464003 | - |
| CR 0238 | winter-type | Sweden | 285,75 | 113,3666667 | 44,75 | 22,58333333 | 0,200213862 | - |
| CR 0834 c | winter-type | Italy | 199,875 | 78,18181818 | 37,58333333 | 18,63636364 | 0,193960444 | - |
| CR 0917 | winter-type | Sweden | 313,375 | 122,1416667 | 46,91666667 | 22,75 | 0,188996494 | - |
| CR 3056 | winter-type | Germany | 263 | 101,4545455 | 44 | 14,33333333 | 0,125663828 | - |
| CR 3155 | winter-type | Germany | 330,1428571 | 126,9166667 | 55,41666667 | 20,83333333 | 0,144522383 | - |
| CR 0871 | winter-type | Netherlands | 204,9166667 | 78,58333333 | 51,08333333 | 20,66666667 | 0,155147353 | - |
| CR 3217 | winter-type | Netherlands | 242,4583333 | 92,84166667 | 42,66666667 | 16,83333333 | 0,151073128 | - |
| CR 0182 c | winter-type | Germany | 431,2 | 162,9 | 58,58333333 | 25,3 | 0,163150637 | - |
| CR 2186 c | winter-type | Germany | 307,75 | 115,6666667 | 67,08333333 | 18,75 | 0,105050179 | - |
| CR 1031 | winter-type | Germany | 221,625 | 82,16666667 | 53,66666667 | 12,66666667 | 0,087505357 | - |
| CR 3278 | winter-type | Netherlands | 292,275 | 108,0416667 | 42,58333333 | 16 | 0,138892864 | - |
| CR 3032 | winter-type | Germany | 288,4444444 | 106,5 | 52 | 18,66666667 | 0,132541188 | - |
| CR 0767 | winter-type | Sweden | 396,6833333 | 145,85 | 45,66666667 | 28,25 | 0,227447737 | - |
| CR 0870 c | winter-type | Germany | 290,75 | 105 | 51,66666667 | 22,33333333 | 0,156103514 | - |
| CR 3079 | winter-type | Germany | 424,3333333 | 152,125 | 65,5 | 22,75 | 0,124518403 | - |
| CR 0164 c | winter-type | Germany | 493 | 175,6363636 | 68,27272727 | 25,45454545 | 0,13282677 | - |
| CR 3091 | winter-type | Germany | 276,7777778 | 97,5 | 45,41666667 | 18,58333333 | 0,144139084 | - |
| CR 2195 | winter-type | Germany | 194,3333333 | 67,66666667 | 46,5 | 8,833333333 | 0,066145324 | - |
| CR 3218 | winter-type | Netherlands | 157,5555556 | 52,55555556 | 52,5 | 9,636363636 | 0,061226409 | - |
| CR 1879 | winter-type | Germany | 189 | 62,75 | 59,5 | 15,66666667 | 0,087420153 | - |
| CR 0875 | winter-type | Germany | 198,6666667 | 65,41666667 | 49,16666667 | 5,5 | 0,036834547 | - |
| CR 2532 | winter-type | x | 409,3333333 | 133 | 57,75 | 13,16666667 | 0,074079558 | - |
| CR 1043 | winter-type | Netherlands | 284,075 | 91,60833333 | 45,16666667 | 17,08333333 | 0,121970988 | - |
| CR 0296 | winter-type | Germany | 208,125 | 66,83333333 | 54,08333333 | 19,16666667 | 0,113802554 | - |
| CR 0172 c | winter-type | Germany | 208,4166667 | 65,16666667 | 51,41666667 | 8,75 | 0,053210482 | - |
| CR 0629 c | winter-type | Germany | 339,1666667 | 102,8181818 | 75,08333333 | 10,54545455 | 0,042577339 | - |
| CR 0743 c | winter-type | Poland | 509,4166667 | 153,0833333 | 67,83333333 | 22,16666667 | 0,098200114 | - |
| CR 3312 | winter-type | Germany | 386,8571429 | 110,8333333 | 62,08333333 | 14,16666667 | 0,065375109 | - |
| CR 0314 | winter-type | Great Britain | 271,25 | 76,83333333 | 41,25 | 15,25 | 0,10471908 | - |
| CR 0984 | winter-type | Sweden | 252,925 | 70,61666667 | 41,58333333 | 14,5 | 0,097356322 | - |
| CR 0318 c | winter-type | Germany | 403,25 | 110,8333333 | 65,75 | 13,08333333 | 0,054691353 | - |
| CR 3140 | winter-type | Germany | 212,2857143 | 57,55555556 | 51,33333333 | 9,888888889 | 0,052229337 | - |
| CR 2268 | winter-type | Italy | 199,75 | 54,13333333 | 38,25 | 10,66666667 | 0,075574497 | - |
| CR 0157 | winter-type | Netherlands | 316,1666667 | 85,58333333 | 41,25 | 9,25 | 0,060700308 | - |
| CR 0972 c | winter-type | France | 397,4444444 | 107,25 | 57,22222222 | 16,41666667 | 0,077417854 | - |
| CR 3214 | winter-type | Germany | 223,2857143 | 60,25 | 50,91666667 | 11,83333333 | 0,062710931 | - |
| CR 2261 | winter-type | Italy | 213,75 | 56 | 41,18181818 | 12,5 | 0,079521836 | - |
| CR 0871 | winter-type | Netherlands | 257,6083333 | 67,44166667 | 45,16666667 | 15,58333333 | 0,090325569 | - |
| CR 0962 | winter-type | Germany | 377,6363636 | 98,08333333 | 53,90909091 | 23,58333333 | 0,113622565 | - |
| CR 0756 | winter-type | Netherlands | 272,8 | 70,325 | 38,41666667 | 9 | 0,060393223 | - |
| CR 0780 c | winter-type | New Zealand | 252,7142857 | 65 | 42,33333333 | 8,083333333 | 0,049112448 | - |
| CR 3150 | winter-type | x | 393,3 | 100,125 | 47,3 | 10,75 | 0,057858332 | - |
| CR 0700 | winter-type | Germany | 264,3636364 | 66,83333333 | 50,36363636 | 8 | 0,040157282 | - |
| CR 3261 | winter-type | France | 323 | 81,58333333 | 71,5 | 9,416666667 | 0,033265195 | - |
| CR 1060 c | winter-type | Germany | 284,125 | 71,1 | 50 | 11 | 0,055053234 | - |
| CR 3136 | winter-type | Germany | 295,4 | 73,7 | 47,25 | 12,08333333 | 0,063803123 | - |
| CR 1058 | winter-type | Netherlands | 304,4166667 | 74,79166667 | 44,58333333 | 16,75 | 0,09230539 | - |
| CR 3064 | winter-type | Germany | 308,75 | 75,5 | 51,91666667 | 13,25 | 0,062409264 | - |
| CR 1058 | winter-type | Netherlands | 165,5 | 40,33333333 | 47,41666667 | 5,916666667 | 0,030409704 | - |
| CR 0279 c | winter-type | Germany | 369,0833333 | 89,75 | 62,41666667 | 15,41666667 | 0,06006202 | - |
| CR 3305 | winter-type | Netherlands | 282,7833333 | 68,74545455 | 39,66666667 | 13,45454545 | 0,082458134 | - |
| CR 3161 | winter-type | Germany | 230 | 55,16666667 | 49,25 | 3,583333333 | 0,017451384 | - |
| CR 0873 | winter-type | New Zealand | 230,9 | 55,16666667 | 42,58333333 | 11,08333333 | 0,062184701 | - |
| CR 0923 | winter-type | x | 455 | 107 | 55,91666667 | 9 | 0,037850674 | - |
| CR 0904 c | winter-type | France | 307,2857143 | 72,16666667 | 44,33333333 | 13,625 | 0,072177261 | - |
| CR 0658 c | winter-type | Germany | 210,4545455 | 49,33333333 | 54,08333333 | 4,583333333 | 0,019865529 | - |
| CR 0178 | winter-type | Netherlands | 290,05 | 67,78333333 | 46,25 | 7,5 | 0,037896542 | - |
| CR 0283 c | winter-type | New Zealand | 307,5 | 71,21666667 | 39,16666667 | 8,666666667 | 0,05124742 | - |
| CR 1107 | winter-type | Sweden | 414,6818182 | 95,54166667 | 56,72727273 | 5 | 0,020307475 | - |
| CR 0952 | winter-type | Germany | 255,75 | 58,5 | 63,33333333 | 6,583333333 | 0,023776817 | - |
| CR 3288 | winter-type | Germany | 199,75 | 45,63636364 | 48 | 4,083333333 | 0,019435595 | - |
| CR 0642 | winter-type | Sweden | 245,1818182 | 55,98333333 | 48,09090909 | 14 | 0,066471509 | - |
| CR 0762 | winter-type | Sweden | 292,7 | 66,75 | 48,16666667 | 15,08333333 | 0,07141333 | - |
| CR 0810 | winter-type | Germany | 173,125 | 39,25 | 45,54545455 | 10 | 0,049777701 | - |
| CR 0874 | winter-type | Great Britain | 263,1 | 58,85 | 46,5 | 7,666666667 | 0,036879009 | - |
| CR 0294 | winter-type | France | 245,6666667 | 54,5 | 68,75 | 8,6 | 0,027750833 | - |
| CR 3029 | winter-type | Germany | 321,3333333 | 71,2 | 48,27272727 | 8,5 | 0,039015871 | - |
| CR 3264 | winter-type | Netherlands | 486,9166667 | 105,5833333 | 42,08333333 | 10,41666667 | 0,053673432 | - |
| CR 0300 | winter-type | New Zealand | 379,075 | 81,175 | 50,83333333 | 7,916666667 | 0,033349623 | - |
| CR 0919 | winter-type | Sweden | 378,475 | 79,93333333 | 54,16666667 | 11,66666667 | 0,045488897 | - |
| CR 0327 c | winter-type | Hungary | 340,5 | 71,83333333 | 46 | 7,75 | 0,035542893 | - |
| CR 3448 | winter-type | Germany | 261,5833333 | 55,16666667 | 50,16666667 | 8,333333333 | 0,035032424 | - |
| CR 0779 | winter-type | x | 615,25 | 129,75 | 59,75 | 8 | 0,028236302 | - |
| CR 3070 | winter-type | Germany | 217,4166667 | 45,81818182 | 50,58333333 | 3,181818182 | 0,013256013 | - |
| CR 3405 | winter-type | Netherlands | 385,5 | 80,45 | 38,75 | 10,83333333 | 0,058343444 | - |
| CR 0780 | winter-type | New Zealand | 281,2416667 | 58,175 | 39,75 | 2,5 | 0,01300947 | - |
| CR 0307 c | winter-type | Germany | 457,5833333 | 94,63636364 | 57,25 | 19,36363636 | 0,069951861 | - |
| CR 3088 | winter-type | Germany | 257,5 | 53,18181818 | 49,16666667 | 7,545454545 | 0,031695718 | - |
| CR 2309 | winter-type | Germany | 294,625 | 60,83333333 | 59,16666667 | 11,08333333 | 0,038678116 | - |
| CR 0982 | winter-type | Sweden | 297,6416667 | 61,31666667 | 47,83333333 | 12,16666667 | 0,052399335 | - |
| CR 1028 | winter-type | Germany | 280,8 | 57,5 | 40,09090909 | 8,416666667 | 0,042989754 | - |
| CR 0776 | winter-type | x | 362,8 | 73,6 | 42,5 | 8 | 0,038186653 | - |
| CR 0877 | winter-type | Germany | 318,875 | 64,44444444 | 63,08333333 | 15 | 0,048055338 | - |
| CR 0276 | winter-type | New Zealand | 284,2857143 | 57,33333333 | 43,16666667 | 10,41666667 | 0,048666757 | - |
| CR 2196 | winter-type | Germany | 258,3333333 | 51,90909091 | 48,41666667 | 9,909090909 | 0,04112462 | - |
| CR 0636 | winter-type | Germany | 231,8571429 | 46,28571429 | 50,66666667 | 2,666666667 | 0,010506859 | - |
| CR 1017 | winter-type | France | 294,5 | 58,16666667 | 44,41666667 | 9,583333333 | 0,042614707 | - |
| CR 0919 | winter-type | Sweden | 405,0416667 | 79,8 | 62,5 | 10,91666667 | 0,034412262 | - |
| CR 0578 | winter-type | Great Britain | 260,1666667 | 51,25 | 40,5 | 6,333333333 | 0,030804881 | - |
| CR 0751 | winter-type | Germany | 358,1428571 | 70,09090909 | 58,58333333 | 12,54545455 | 0,041910006 | - |
| CR 0715 c | winter-type | Germany | 403,25 | 78,66666667 | 57,66666667 | 8,833333333 | 0,029882446 | - |
| CR 3379 | winter-type | Germany | 262,4166667 | 51,16666667 | 53,75 | 4,75 | 0,017231015 | - |
| CR 0954 c | winter-type | Germany | 299,3636364 | 58,33333333 | 52,09090909 | 10,41666667 | 0,038965888 | - |
| CR 0717 | winter-type | Germany | 266,0909091 | 51,8 | 50,5 | 2,5 | 0,009637144 | - |
| CR 0561 c | winter-type | Germany | 486,6363636 | 94,63636364 | 63,27272727 | 8,181818182 | 0,025147033 | - |
| CR 3267 | winter-type | Germany | 235,5833333 | 45,58333333 | 45 | 6,833333333 | 0,029382017 | - |
| CR 3196 | winter-type | Germany | 228,7777778 | 44,16666667 | 49,5 | 7,666666667 | 0,029900756 | - |
| CR 3183 | winter-type | Germany | 333 | 62,58333333 | 53,27272727 | 11,66666667 | 0,041158195 | - |
| CR 0718 c | winter-type | Germany | 459,5833333 | 85,41666667 | 79,25 | 17,83333333 | 0,041822656 | - |
| CR 3025 | winter-type | Germany | 386,1666667 | 71,41666667 | 69,08333333 | 14,83333333 | 0,03970912 | - |
| CR 0304 c | winter-type | Great Britain | 226,4 | 41,39166667 | 43,66666667 | 4,666666667 | 0,019538591 | - |
| CR 0762 | winter-type | Sweden | 469,8833333 | 85,85 | 49,33333333 | 14 | 0,051848695 | - |
| CR 0838 | winter-type | Germany | 220,375 | 40,09090909 | 44,9 | 4,75 | 0,019245573 | - |
| CR 0277 | winter-type | Great Britain | 245,8571429 | 44,71428571 | 42,08333333 | 3,571428571 | 0,015434597 | - |
| CR 0872 c | winter-type | France | 333,875 | 60 | 42,83333333 | 9,416666667 | 0,039507784 | - |
| CR 3129 | winter-type | x | 349,1416667 | 62,11666667 | 45,25 | 4,916666667 | 0,019331192 | - |
| CR 3190 | winter-type | Germany | 289,4166667 | 50,5 | 49,66666667 | 5,5 | 0,019322598 | - |
| CR 3160 | winter-type | Sweden | 331,2666667 | 57,58333333 | 50,91666667 | 13,5 | 0,046088531 | - |
| CR 3151 | winter-type | Germany | 417,625 | 72,09090909 | 51,33333333 | 11,27272727 | 0,037907361 | - |
| CR 0647 | winter-type | Great Britain | 260,4285714 | 44,5 | 40,75 | 5,333333333 | 0,02236364 | - |
| CR 0627 | winter-type | x | 355,0416667 | 60,575 | 41,33333333 | 4,583333333 | 0,018918866 | - |
| CR 0788 | winter-type | x | 348 | 59,3 | 52,3 | 6,2 | 0,020200655 | - |
| CR 0321 | winter-type | Great Britain | 292,25 | 49,66666667 | 42,33333333 | 9,142857143 | 0,03670371 | - |
| CR 3115 | winter-type | Netherlands | 272,625 | 46,25 | 46,25 | 12,41666667 | 0,045544857 | - |
| CR 1057 | winter-type | Netherlands | 193,4166667 | 32,75 | 49,25 | 2,916666667 | 0,010027622 | - |
| CR 0686 c | winter-type | Germany | 225,1666667 | 38 | 49,75 | 2,083333333 | 0,007067164 | - |
| CR 0879 | winter-type | x | 466,2 | 78,3 | 50,5 | 9,5 | 0,031595244 | - |
| CR 0169 | winter-type | Sweden | 455,2166667 | 76,31666667 | 52,33333333 | 7,333333333 | 0,023492231 | - |
| CR 0290 | winter-type | France | 373,375 | 62,375 | 43,4 | 8,125 | 0,031275118 | - |
| CR 1860 | winter-type | Netherlands | 363,5 | 60,5 | 43,75 | 9,416666667 | 0,035823672 | - |
| CR 1107 | winter-type | Sweden | 233,1111111 | 38,33333333 | 50,08333333 | 6,666666667 | 0,021889162 | - |
| CR 0736 | winter-type | Germany | 275 | 44,75 | 46,25 | 9,583333333 | 0,033718264 | - |
| CR 2192 | winter-type | Germany | 251,5 | 40,91666667 | 46,83333333 | 9,25 | 0,032132826 | - |
| CR 3044 | winter-type | Germany | 263,9166667 | 42,81818182 | 52,83333333 | 9,727272727 | 0,029870637 | - |
| CR 3275 | winter-type | Germany | 425,125 | 68,83333333 | 70,83333333 | 9,083333333 | 0,020762982 | - |
| CR 3180 | winter-type | x | 357,3 | 57,6 | 43,25 | 3,9 | 0,014536771 | - |
| CR 0950 c | winter-type | x | 286,3833333 | 45,94166667 | 40,58333333 | 4,083333333 | 0,01614084 | - |
| CR 0192 c | winter-type | Netherlands | 248 | 39,65 | 38,16666667 | 26,66666667 | 0,111705874 | - |
| CR 3138 | winter-type | Germany | 234,6 | 37,45454545 | 46,4 | 5,181818182 | 0,017829564 | - |
| CR 0754 c | winter-type | Netherlands | 331,0833333 | 52,66666667 | 44,08333333 | 17,33333333 | 0,062546956 | - |
| CR 3307 | winter-type | Germany | 376,1428571 | 59,8 | 53,16666667 | 7,666666667 | 0,022925326 | - |
| CR 0858 c | winter-type | France | 457,75 | 72,58333333 | 77,33333333 | 12,41666667 | 0,025459323 | - |
| CR 0925 c | winter-type | x | 304,7916667 | 48,325 | 41,75 | 4,416666667 | 0,016772852 | - |
| CR 0963 | winter-type | Germany | 198,5 | 31,41666667 | 49,08333333 | 1,916666667 | 0,006180337 | - |
| CR 3176 | winter-type | Germany | 294,6666667 | 46,2 | 57 | 6,6 | 0,018154322 | - |
| CR 2197 | winter-type | Italy | 244,2857143 | 37,8 | 34,75 | 7,8 | 0,034732298 | - |
| CR 1038 | winter-type | Sweden | 460,9333333 | 71,08333333 | 49,66666667 | 6,333333333 | 0,019665138 | - |
| CR 3258 | winter-type | Germany | 350,2 | 54 | 54,58333333 | 6,272727273 | 0,017720418 | - |
| CR 0884 c | winter-type | Germany | 426 | 64,41666667 | 68,33333333 | 16,08333333 | 0,035590337 | - |
| CR 3216 | winter-type | Germany | 260,5833333 | 39,33333333 | 51,41666667 | 6,666666667 | 0,019571267 | - |
| CR 3157 | winter-type | Germany | 378,7 | 57 | 58,5 | 12,75 | 0,032804534 | - |
| CR 0985 | winter-type | Sweden | 310 | 46,58333333 | 39,36363636 | 8,416666667 | 0,032130226 | - |
| CR 2187 c | winter-type | Germany | 243,0833333 | 36,33333333 | 47,75 | 6,166666667 | 0,019303104 | - |
| CR 0961 | winter-type | Germany | 250 | 37,08333333 | 52,72727273 | 6,333333333 | 0,01781705 | - |
| CR 0740 c | winter-type | Germany | 452,9166667 | 67,08333333 | 68,33333333 | 11,75 | 0,025468396 | - |
| CR 3104 | winter-type | Sweden | 340,55 | 50,4 | 38,66666667 | 7,5 | 0,028706099 | - |
| CR 0641 | winter-type | Sweden | 356,5363636 | 52,65 | 50 | 6,5 | 0,0191972 | - |
| CR 0942 | winter-type | x | 482,8 | 70,8 | 44,8 | 5,3 | 0,017348577 | - |
| CR 1109 | winter-type | Sweden | 273,3 | 39,66666667 | 44,58333333 | 5,666666667 | 0,018447657 | - |
| CR 0851 | winter-type | x | 367 | 53 | 52 | 3 | 0,008331587 | - |
| CR 3294 | winter-type | France | 230,7142857 | 33,27272727 | 36,75 | 6,916666667 | 0,027142723 | - |
| CR 3049 | winter-type | Germany | 371 | 53,5 | 61,83333333 | 3,666666667 | 0,008551231 | - |
| CR 1106 | winter-type | Sweden | 511,275 | 73,525 | 54,91666667 | 9,25 | 0,024222448 | - |
| CR 3215 | winter-type | Germany | 271,5 | 38,72727273 | 58,91666667 | 4,363636364 | 0,010564707 | - |
| CR 0733 | winter-type | Netherlands | 506,6666667 | 71,66666667 | 39,08333333 | 13,66666667 | 0,04946134 | - |
| CR 0782 | winter-type | x | 309 | 43,6 | 44,3 | 3,2 | 0,010192348 | - |
| CR 3449 | winter-type | Germany | 256,6 | 36 | 56,6 | 36 | 0,08923432 | - |
| CR 0981 | winter-type | Sweden | 322,5714286 | 45,14285714 | 35,33333333 | 9,083333333 | 0,035976904 | - |
| CR 0645 | winter-type | Sweden | 325,3 | 45,45 | 52,5 | 4,083333333 | 0,010866892 | - |
| CR 0577 | winter-type | New Zealand | 315,075 | 43,86666667 | 41,75 | 2,416666667 | 0,008058996 | - |
| CR 1036 | winter-type | Sweden | 456,9333333 | 63,44166667 | 59,91666667 | 6,083333333 | 0,014096643 | - |
| CR 3065 | winter-type | Germany | 211,4166667 | 29,33333333 | 44,08333333 | 2,916666667 | 0,009179829 | - |
| CR 0638 | winter-type | x | 282,8 | 39,1 | 41,6 | 1,75 | 0,005816237 | - |
| CR 0873 | winter-type | New Zealand | 422,4583333 | 58,40833333 | 45,91666667 | 5,25 | 0,015808108 | - |
| CR 0300 | winter-type | New Zealand | 276,9166667 | 38,16666667 | 45,75 | 6,5 | 0,019582016 | - |
| CR 0292 | winter-type | x | 56,6 | 7,8 | 56 | 8 | 0,019687027 | - |
| CR 3149 | winter-type | x | 370,5333333 | 50,5 | 53,33333333 | 4,333333333 | 0,011073565 | - |
| CR 3124 | winter-type | Germany | 270,5555556 | 36,83333333 | 56,22222222 | 3,833333333 | 0,009282248 | - |
| CR 0760 | winter-type | France | 271,5714286 | 36,83333333 | 40,83333333 | 6 | 0,01992936 | - |
| CR 3024 | winter-type | Germany | 344,2857143 | 46,5 | 69,18181818 | 8,5 | 0,016594375 | - |
| CR 3159 | winter-type | x | 316,9 | 42,63333333 | 42,83333333 | 4,083333333 | 0,012825078 | - |
| CR 0299 c | winter-type | x | 289,8 | 38,8 | 45,75 | 6,7 | 0,019607266 | - |
| CR 0981 | winter-type | Sweden | 359,5583333 | 47,96666667 | 37,16666667 | 6,583333333 | 0,023629929 | - |
| CR 0688 | winter-type | x | 416,9083333 | 55,2 | 52,75 | 7,5 | 0,0188251 | - |
| CR 0285 c | winter-type | Germany | 464,5833333 | 61,5 | 61,58333333 | 8,25 | 0,017733818 | - |
| CR 0772 | winter-type | x | 463 | 61 | 56 | 7 | 0,016468683 | - |
| CR 3263 | winter-type | x | 390,4 | 51,2 | 50,1 | 7 | 0,018324008 | - |
| CR 0985 | winter-type | Sweden | 380,7166667 | 49,875 | 47,08333333 | 4,416666667 | 0,012288771 | - |
| CR 0643 | winter-type | x | 406 | 53 | 54 | 4 | 0,009669768 | - |
| CR 0983 | winter-type | Sweden | 434,7272727 | 56,59166667 | 43,90909091 | 3,833333333 | 0,011364694 | - |
| CR 0787 c | winter-type | x | 407,8 | 52,85 | 48,4 | 5,4 | 0,014459263 | - |
| CR 0794 | winter-type | x | 332,4 | 43 | 54,3 | 2,8 | 0,006670611 | - |
| CR 0276 | winter-type | x | 374,725 | 48,38333333 | 47,75 | 6,75 | 0,018252128 | - |
| CR 0167 c | winter-type | New Zealand | 227,4 | 29,22222222 | 44,2 | 5,916666667 | 0,017201947 | - |
| CR 0659 c | winter-type | Poland | 630,0833333 | 80,75 | 68,66666667 | 12,83333333 | 0,023951794 | - |
| CR 0948 c | winter-type | x | 320,6 | 41 | 42,3 | 4,7 | 0,014209468 | - |
| CR 3184 | winter-type | Germany | 449,5555556 | 57,41666667 | 67 | 6,583333333 | 0,012549478 | - |
| CR 0893 | winter-type | x | 452,3 | 57,4 | 46,3 | 6,4 | 0,017542209 | - |
| CR 0550 | winter-type | France | 369,4285714 | 46,42857143 | 46,66666667 | 5 | 0,013465363 | - |
| CR 1018 | winter-type | x | 471,0583333 | 59,15833333 | 47,08333333 | 4 | 0,010669253 | - |
| CR 0966 | winter-type | Germany | 407,4 | 51,11111111 | 61,25 | 7,583333333 | 0,01553275 | - |
| CR 3271 | winter-type | Sweden | 340,55 | 42,58333333 | 52,41666667 | 4,166666667 | 0,009939811 | - |
| CR 3074 | winter-type | Germany | 264,4166667 | 33 | 49,41666667 | 3,166666667 | 0,007997496 | - |
| CR 0286 c | winter-type | Germany | 412,75 | 51,5 | 72,91666667 | 8,583333333 | 0,014687549 | - |
| CR 3048 | winter-type | Germany | 369,2727273 | 45,90909091 | 55,90909091 | 4,636363636 | 0,010309712 | - |
| CR 0789 | winter-type | Germany | 352,0833333 | 43,66666667 | 51,5 | 14 | 0,033715172 | - |
| CR 0641 | winter-type | Sweden | 462,7727273 | 57,375 | 57,09090909 | 6,416666667 | 0,013934695 | - |
| CR 3067 | winter-type | Germany | 274,5833333 | 33,41666667 | 43,5 | 6,916666667 | 0,019350694 | - |
| CR 3116 | winter-type | France | 315 | 38,33333333 | 51,75 | 9 | 0,021164021 | - |
| CR 0847 | winter-type | Germany | 333,25 | 40,5 | 59,58333333 | 4,333333333 | 0,008838573 | - |
| CR 0165 | winter-type | x | 313 | 38 | 44,5 | 1,6 | 0,004365151 | - |
| CR 3063 | winter-type | Germany | 422,4444444 | 51,27272727 | 56,18181818 | 8,090909091 | 0,017479071 | - |
| CR 0894 | winter-type | x | 475,4666667 | 57,475 | 55,83333333 | 9,166666667 | 0,019846174 | - |
| CR 0947 | winter-type | x | 390,7 | 47,2 | 48,4 | 4,8 | 0,011981038 | - |
| CR 3204 | winter-type | Germany | 316,2222222 | 38,16666667 | 50,90909091 | 8 | 0,018966469 | - |
| CR 0672 | winter-type | x | 399 | 48 | 58 | 4 | 0,008296604 | - |
| CR 0800 c | winter-type | Germany | 227,4166667 | 27,16666667 | 51,33333333 | 4,75 | 0,011053714 | - |
| CR 0856 | winter-type | x | 339,5916667 | 40,54166667 | 48 | 6,333333333 | 0,015751999 | - |
| CR 0771 | winter-type | x | 371,3 | 44,1 | 54,8 | 2,8 | 0,006068636 | - |
| CR 3018 | winter-type | Germany | 266,4166667 | 31,5 | 54 | 3,083333333 | 0,006751121 | - |
| CR 3163 | winter-type | Germany | 312,8333333 | 36,91666667 | 58,75 | 4,583333333 | 0,009206256 | - |
| CR 0673 | winter-type | x | 432,9 | 51 | 54 | 2,8 | 0,006108673 | - |
| CR 3066 | winter-type | Germany | 256,5 | 30,11111111 | 40,33333333 | 4,6 | 0,013388537 | - |
| CR 1038 | winter-type | Sweden | 319,3 | 37,33333333 | 45,08333333 | 6 | 0,015560842 | - |
| CR 0325 | winter-type | Great Britain | 324,8571429 | 37,875 | 45,08333333 | 4 | 0,010344374 | - |
| CR 3304 | winter-type | x | 413,6 | 48,2 | 53 | 4,1 | 0,009015182 | - |
| CR 0310 | winter-type | Great Britain | 260,5 | 30,33333333 | 42,08333333 | 3,666666667 | 0,010145506 | - |
| CR 0983 | winter-type | Sweden | 283,3416667 | 32,73333333 | 43,66666667 | 6,666666667 | 0,017637559 | - |
| CR 3083 | winter-type | Germany | 380,75 | 43,83333333 | 50,08333333 | 4,083333333 | 0,009386122 | - |
| CR 2193 | winter-type | France | 380,5 | 43,75 | 41,75 | 7,625 | 0,020999394 | - |
| CR 0238 | winter-type | x | 413,4363636 | 47,47272727 | 42,90909091 | 15,45454545 | 0,041356372 | - |
| CR 1041 | winter-type | x | 323 | 37 | 46,9 | 5,8 | 0,014166232 | - |
| CR 0975 | winter-type | Germany | 236,4545455 | 27,08333333 | 52 | 4,666666667 | 0,010279166 | - |
| CR 0287 | winter-type | Germany | 318,75 | 36,41666667 | 48,25 | 4,454545455 | 0,010547659 | - |
| CR 0293 | winter-type | x | 306,4 | 35 | 42,3 | 4,2 | 0,011341962 | - |
| CR 0880 c | winter-type | Germany | 234,25 | 26,66666667 | 53,83333333 | 1,5 | 0,00317197 | - |
| CR 0719 c | winter-type | Germany | 436,0833333 | 49,25 | 57,83333333 | 5,666666667 | 0,011065886 | - |
| CR 0707 c | winter-type | France | 373,5833333 | 42,16666667 | 65,66666667 | 8,181818182 | 0,01406328 | - |
| CR 0984 | winter-type | Sweden | 346,3416667 | 39 | 47,75 | 2,916666667 | 0,006878176 | - |
| CR 0645 | winter-type | Sweden | 318,8090909 | 35,46 | 49,72727273 | 6,166666667 | 0,013793163 | - |
| CR 0642 | winter-type | Sweden | 334,9333333 | 37,24166667 | 49,83333333 | 3 | 0,00669379 | - |
| CR 0177 | winter-type | x | 413,2636364 | 45,85 | 44,81818182 | 5,5 | 0,013615093 | - |
| CR 3211 | winter-type | Germany | 348,1666667 | 38,6 | 52,25 | 3,416666667 | 0,00724964 | - |
| CR 1110 | winter-type | Sweden | 534,5666667 | 58,875 | 55,83333333 | 3,5 | 0,006904044 | - |
| CR 3082 | winter-type | Netherlands | 378,9 | 41,66666667 | 50,66666667 | 9,166666667 | 0,019895427 | - |
| CR 0328 | winter-type | x | 257 | 28 | 48 | 1,9 | 0,004312581 | - |
| CR 0892 | winter-type | x | 377 | 41 | 42,3 | 4,9 | 0,012597902 | - |
| CR 0298 c | winter-type | Germany | 218,4545455 | 23,66666667 | 52,08333333 | 3,916666667 | 0,008146927 | - |
| CR 3271 | winter-type | Sweden | 421,75 | 45,61666667 | 47,75 | 4,166666667 | 0,009438084 | - |
| CR 0167 | winter-type | New Zealand | 199,1 | 21,49166667 | 37,25 | 2,166666667 | 0,006278627 | - |
| CR 0265 | winter-type | x | 296,4583333 | 31,75833333 | 41,16666667 | 1,583333333 | 0,004120223 | - |
| CR 0767 | winter-type | Sweden | 590,6416667 | 63,15 | 58,25 | 6,083333333 | 0,011165931 | - |
| CR 0778 | winter-type | x | 318,7 | 34 | 46,2 | 4 | 0,009236658 | - |
| CR 3280 | winter-type | Netherlands | 303,1111111 | 32,16666667 | 45,66666667 | 4,166666667 | 0,009682637 | - |
| CR 1191 c | winter-type | Germany | 196 | 20,75 | 47,36363636 | 1,333333333 | 0,002980271 | - |
| CR 0312 | winter-type | Ireland | 298,5 | 31,42857143 | 42,08333333 | 2,555555556 | 0,006393748 | - |
| CR 0949 | winter-type | Hungary | 174,7363636 | 18,34166667 | 33,63636364 | 4 | 0,01248264 | - |
| CR 1015 | winter-type | France | 312,7777778 | 32,71428571 | 42,54545455 | 5,571428571 | 0,013696669 | - |
| CR 1026 c | winter-type | x | 362,6 | 37,7 | 45,3 | 5,7 | 0,013082484 | - |
| CR 0837 c | winter-type | France | 305,3333333 | 31,66666667 | 46,54545455 | 6,833333333 | 0,015225917 | - |
| CR 0784 | winter-type | x | 460,9166667 | 47,58333333 | 69,33333333 | 2,916666667 | 0,004342873 | - |
| CR 0579 | winter-type | Germany | 363,6666667 | 37,33333333 | 57,08333333 | 15,25 | 0,027425452 | - |
| CR 0683 | winter-type | Great Britain | 322 | 33 | 41,66666667 | 3,428571429 | 0,008433008 | - |
| CR 3058 | winter-type | Germany | 367,4 | 37,5 | 61,83333333 | 5,833333333 | 0,009629112 | - |
| CR 3119 | winter-type | Germany | 338,8333333 | 34,58333333 | 57,58333333 | 5,166666667 | 0,009157868 | - |
| CR 0862 c | winter-type | x | 514,1 | 51,8 | 55,9 | 5,7 | 0,010274133 | - |
| CR 0241 c | winter-type | Germany | 418,75 | 41,72727273 | 56,16666667 | 7,181818182 | 0,012741511 | - |
| CR 0261 | winter-type | x | 459,8083333 | 45,425 | 44,91666667 | 3,833333333 | 0,008431157 | - |
| CR 0799 | winter-type | Sweden | 370,8181818 | 36,33333333 | 42,18181818 | 5,166666667 | 0,01200133 | - |
| CR 1108 | winter-type | Sweden | 699,11 | 68,48888889 | 59,4 | 9,777777778 | 0,016126062 | - |
| CR 3201 | winter-type | Germany | 333 | 32,58333333 | 54,33333333 | 3 | 0,005402642 | - |
| CR 1863 | winter-type | Great Britain | 333,2 | 32,6 | 43,58333333 | 4 | 0,0089795 | - |
| CR 1047 | winter-type | x | 395,9 | 38,3 | 49,5 | 4,25 | 0,008306097 | - |
| CR 1110 | winter-type | Sweden | 295,375 | 28,5 | 49,41666667 | 3,666666667 | 0,007159276 | - |
| CR 0866 | winter-type | France | 423,5 | 40,71428571 | 40,83333333 | 7,416666667 | 0,017461733 | - |
| CR 3306 | winter-type | x | 411,2 | 39,49166667 | 49,08333333 | 3,833333333 | 0,007500581 | - |
| CR 0160 c | winter-type | Sweden | 269,1111111 | 25,83333333 | 44,5 | 2,5 | 0,00539298 | - |
| CR 3068 | winter-type | Germany | 407,2222222 | 38,91666667 | 60,09090909 | 6,5 | 0,010337339 | - |
| CR 0181 c | winter-type | Germany | 434,5 | 41 | 65,16666667 | 3,833333333 | 0,005550667 | - |
| CR 0682 c | winter-type | x | 255 | 24 | 40,9 | 1,8 | 0,004142097 | - |
| CR 1008 c | winter-type | x | 476,25 | 44,33333333 | 67,75 | 2,5 | 0,003434995 | - |
| CR 3104 | winter-type | Sweden | 430,1 | 39,81666667 | 42,25 | 5,083333333 | 0,01113826 | - |
| CR 0330 | winter-type | Sweden | 392,0166667 | 35,96666667 | 49,75 | 3,75 | 0,006915663 | - |
| CR 3160 | winter-type | Sweden | 423,9916667 | 38,80833333 | 52,83333333 | 4,833333333 | 0,008373488 | - |
| CR 0331 | winter-type | Sweden | 467,3166667 | 42,68333333 | 46 | 3,166666667 | 0,006287697 | - |
| CR 3127 | winter-type | Germany | 311,9166667 | 28,41666667 | 54,41666667 | 3,833333333 | 0,006417697 | - |
| CR 1109 | winter-type | Sweden | 472,1916667 | 42,93333333 | 54 | 2,666666667 | 0,004490051 | - |
| CR 0302 | winter-type | x | 471,8 | 42,7 | 53,6 | 4,6 | 0,007767173 | - |
| CR 3302 | winter-type | x | 445,325 | 39,6 | 52 | 0,9 | 0,001539066 | - |
| CR 0982 | winter-type | Sweden | 448,725 | 39,875 | 50,66666667 | 4,083333333 | 0,007161647 | - |
| CR 0329 | winter-type | x | 343 | 30 | 49,4 | 1,7 | 0,003009879 | - |
| CR 0949 | winter-type | Hungary | 293 | 25,33333333 | 40,41666667 | 4,833333333 | 0,010339772 | - |
| CR 3193 | winter-type | France | 315,6 | 27,28571429 | 41,36363636 | 5,375 | 0,011234612 | - |
| CR 0799 | winter-type | Sweden | 497,6083333 | 41,06363636 | 49,33333333 | 8,545454545 | 0,014294352 | - |
| CR 3147 | winter-type | Germany | 344,7 | 28,33333333 | 54,36363636 | 3,583333333 | 0,005417951 | - |
| CR 0646 c | winter-type | x | 494,3333333 | 39,58333333 | 71,75 | 2,416666667 | 0,00269704 | - |
| CR 1019 | winter-type | Germany | 291,2 | 23,27272727 | 60,3 | 2,272727273 | 0,003012215 | - |
| CR 3219 | winter-type | Austria | 416 | 32,8 | 40,9 | 3,1 | 0,005976114 | - |
| CR 0670 | winter-type | France | 321,4285714 | 24,83333333 | 43,7 | 4,5 | 0,007955759 | - |
| CR 1007 | winter-type | Germany | 249 | 19,16666667 | 50,58333333 | 2,833333333 | 0,00431159 | - |
| CR 0577 | winter-type | New Zealand | 323,3333333 | 24,71428571 | 46,58333333 | 2,571428571 | 0,004219311 | - |
| CR 2266 | winter-type | x | 559,0833333 | 41,83333333 | 66,08333333 | 1 | 0,00113228 | - |
| CR 0330 | winter-type | Sweden | 349,725 | 26,16666667 | 51,66666667 | 3,5 | 0,005068499 | - |
| CR 2191 | winter-type | x | 409,4 | 30,2 | 43,2 | 4,2 | 0,007171742 | - |
| CR 0331 | winter-type | Sweden | 343,8333333 | 25,33333333 | 45,66666667 | 2,333333333 | 0,003764626 | - |
| CR 0184 | winter-type | x | 514 | 37 | 56,6 | 1,9 | 0,002416439 | - |
| CR 0283 | winter-type | New Zealand | 370,1666667 | 26,5 | 55,8 | 3,75 | 0,004811114 | - |
| CR 3175 | winter-type | x | 564,75 | 40,41666667 | 57,66666667 | 4,833333333 | 0,005998272 | - |
| CR 0444 | winter-type | x | 240,825 | 17,05 | 32,33333333 | 1,166666667 | 0,002554578 | - |
| CR 1039 c | winter-type | x | 486 | 34,3 | 47,5 | 1,25 | 0,001857267 | - |
| CR 0327 | winter-type | Hungary | 455,2 | 31,5 | 50,8 | 3,4 | 0,00463152 | - |
| CR 0576 | winter-type | Germany | 449,5 | 30,44444444 | 65,41666667 | 2,555555556 | 0,002645911 | - |
| CR 0917 | winter-type | x | 480,1166667 | 32,36666667 | 47,25 | 3,083333333 | 0,004399161 | - |
| CR 2198 c | winter-type | x | 443,0833333 | 29,75 | 54,16666667 | 3,083333333 | 0,003821993 | - |
| CR 3059 | winter-type | x | 421 | 28 | 46,3 | 1,75 | 0,002513813 | - |
| CR 0160 | winter-type | Sweden | 419,45 | 27,6 | 51,5 | 1,75 | 0,002235938 | - |
| CR 2712 | winter-type | x | 509,8333333 | 33,41666667 | 58,91666667 | 2,166666667 | 0,002410398 | - |
| CR 3055 | winter-type | x | 365,8 | 23,7 | 46,3 | 3,2 | 0,004477892 | - |
| CR 0655 | winter-type | x | 481,3333333 | 30,33333333 | 69,16666667 | 3,25 | 0,002961152 | - |
| CR 1250 | winter-type | x | 454 | 26,3 | 45,5 | 3,1 | 0,003946846 | - |
| CR 0155 | winter-type | x | 552,5833333 | 32 | 64,08333333 | 5,25 | 0,004744237 | - |
| CR 0783 | winter-type | x | 496,9166667 | 28,66666667 | 68,91666667 | 2,5 | 0,002092712 | - |
| CR 0175 | winter-type | x | 497,4166667 | 28,33333333 | 64,41666667 | 3 | 0,002652775 | - |
| CR 0156 c | winter-type | x | 625,0909091 | 34,41666667 | 64,90909091 | 3,75 | 0,00318091 | - |
| CR 3172 | winter-type | x | 522,5 | 27,08333333 | 56,41666667 | 1,916666667 | 0,001760982 | - |
| CR 0907 | winter-type | x | 467,25 | 23,75 | 51,83333333 | 1,166666667 | 0,001144068 | - |
| CR 3173 | winter-type | Germany | 338,6363636 | 16,83333333 | 53,63636364 | 4,333333333 | 0,004016052 | - |
| CR 0750 | winter-type | France | 396 | 19,16666667 | 46,5 | 2,666666667 | 0,002775666 | - |
| CR 3380 | winter-type | x | 675 | 28,66666667 | 66,45454545 | 3,083333333 | 0,001970467 | - |
| CR 0774 c | winter-type | x | 578,4166667 | 22,75 | 72,58333333 | 1,5 | 0,000812821 | - |
| CR 0773 c | winter-type | x | 493,8333333 | 17,33333333 | 69,08333333 | 1,5 | 0,000762114 | - |
| CR 0281 | winter-type | x | 693,6666667 | 23,83333333 | 70,25 | 2,083333333 | 0,001018935 | - |
| CR 2237 | winter-type | x | 571,1666667 | 16,5 | 49,91666667 | 2,181818182 | 0,001262682 | - |
| CR 0171 | winter-type | x | 647,4166667 | 15,9 | 73,83333333 | 2,6 | 0,000864837 | - |
| CR 0162 | spring-type | Canada | 432,5 | 57,5 | 64,3 | 11,75 | 0,024294537 | -0,629310345 |
| CR 0163 | spring-type | Canada | 267,7 | 47 | 57,5 | 10 | 0,030533855 | -0,203125 |
| CR 0173 | spring-type | Germany | 424,3 | 98,6 | 54,25 | 10,75 | 0,046048194 | -0,36492891 |
| CR 0174 | spring-type | Germany | 415,2 | 122,1 | 62,75 | 27,3 | 0,127940262 | -0,266129032 |
| CR 0183 | spring-type | Denmark | 419,2 | 93,8 | 57,9 | 10,1 | 0,039032321 | -0,363309353 |
| CR 0234 | spring-type | Germany | 328,3 | 114 | 55 | 18,2 | 0,11490599 | -0,283980583 |
| CR 0235 | spring-type | Finland | 346 | 108 | 61,3 | 21,1 | 0,1074409 | -0,319548872 |
| CR 0268 | spring-type | France | 348,1 | 55,5 | 56,5 | 9,8 | 0,027654549 | -0,257075472 |
| CR 0270 c | spring-type | Poland | 246,7 | 34,25 | 49,75 | 3,4 | 0,009488056 | -0,409836066 |
| CR 0271 | spring-type | France | 206,6 | 129,8 | 53,5 | 35,4 | 0,415713239 | -0,273333333 |
| CR 0280 c | spring-type | Belarus | 312 | 111 | 55 | 14 | 0,090559441 | -0,403954802 |
| CR 0288 | spring-type | France | 394 | 175 | 62,7 | 27,5 | 0,194808086 | -0,278074866 |
| CR 0291 | spring-type | Poland | 290,8 | 71,2 | 51,5 | 11,7 | 0,055624257 | -0,189453125 |
| CR 0303 c | spring-type | Germany | 379,2 | 61 | 52,3 | 11,1 | 0,034141516 | -0,279545455 |
| CR 0316 | spring-type | Poland | 300,2 | 75,8 | 49,75 | 16,25 | 0,08247433 | -0,417218543 |
| CR 0317 | spring-type | Germany | 272 | 12 | 25,3 | 0,7 | 0,001220646 | -0,27393617 |
| CR 0546 | spring-type | Germany | 434 | 32 | 53,4 | 1,8 | 0,002485373 | -0,547945205 |
| CR 0547 | spring-type | Germany | 402 | 36 | 55,9 | 2,3 | 0,003684618 | -0,421052632 |
| CR 0581 | spring-type | Germany | 430 | 101,25 | 69,1 | 14 | 0,047706391 | -0,311619718 |
| CR 0624 | spring-type | Canada | 316,8 | 96,2 | 63,7 | 17,4 | 0,082946815 | -0,262195122 |
| CR 0626 | spring-type | Denmark | 446 | 20 | 54 | 3 | 0,002491281 | -0,651685393 |
| CR 0632 | spring-type | Guatemala | 372,75 | 156 | 54 | 29,5 | 0,228631046 | -0,27259887 |
| CR 0633 | spring-type | Sweden | 333,4 | 56,3 | 52,5 | 8,9 | 0,028626846 | -0,197164948 |
| CR 0634 | spring-type | Sweden | 354 | 25 | 65 | 3 | 0,003259452 | -0,375 |
| CR 0637 c | spring-type | Australia | 333 | 223 | 56,4 | 10,5 | 0,124672545 | -0,306818182 |
| CR 0639 | spring-type | Sweden | 378 | 43 | 60,1 | 10 | 0,018927889 | -0,382608696 |
| CR 0674 | spring-type | Germany | 380,3 | 67 | 65,1 | 7,1 | 0,019214356 | -0,315340909 |
| CR 0677 | spring-type | Germany | 321,25 | 42,25 | 54,7 | 4,7 | 0,011300408 | -0,281879195 |
| CR 0679 | spring-type | Germany | 204 | 11 | 46,8 | 1 | 0,00115217 | -0,3625 |
| CR 0680 c | spring-type | Russia | 363 | 76,2 | 59,4 | 11 | 0,038873584 | -0,302681992 |
| CR 0687 | spring-type | Great Britain | 429,5 | 201,9 | 62,1 | 32,2 | 0,243745958 | -0,307522124 |
| CR 0704 c | spring-type | Germany | 377 | 26 | 60,3 | 2 | 0,002287414 | -0,270042194 |
| CR 0706 | spring-type | Germany | 374 | 38 | 55,3 | 2,8 | 0,00514452 | -0,468309859 |
| CR 0724 | spring-type | Germany | 467 | 52 | 54,8 | 2,3 | 0,004673408 | -0,323308271 |
| CR 0725 | spring-type | Germany | 365,1 | 55,3 | 56,25 | 3,6 | 0,009693783 | -0,269230769 |
| CR 0728 | spring-type | Germany | 432 | 94 | 56,3 | 10,1 | 0,039035261 | -0,357142857 |
| CR 0730 | spring-type | Germany | 435 | 15 | 59,6 | 1,1 | 0,000636427 | - |
| CR 0734 | spring-type | Germany | 537 | 14 | 44,5 | 0,9 | 0,000527274 | -0,219626168 |
| CR 0752 | spring-type | Germany | 516 | 57 | 46,9 | 5,3 | 0,012483265 | -0,397540984 |
| CR 0764 | spring-type | France | 484,8 | 66,6 | 61 | 10,5 | 0,023646729 | -0,472972973 |
| CR 0765 | spring-type | Australia | 358,2 | 186,1 | 51,1 | 33,3 | 0,33856661 | -0,494535519 |
| CR 0766 | spring-type | Germany | 409 | 87 | 56,7 | 19,7 | 0,0739059 | -0,428571429 |
| CR 0775 | spring-type | Canada | 284 | 39,8 | 49,8 | 33,4 | 0,093990045 | -0,164233577 |
| CR 0790 | spring-type | Marocco | 289,6 | 111 | 56,1 | 17,6 | 0,120246994 | -0,291878173 |
| CR 0796 | spring-type | Vietnam | 555 | 65 | 64,8 | 11,3 | 0,020423201 | -0,301136364 |
| CR 0802 | spring-type | Russia | 388 | 46 | 54,2 | 7,1 | 0,01553049 | -0,271028037 |
| CR 0807 c | spring-type | Canada | 465 | 51 | 58 | 3 | 0,00567297 | -0,246453901 |
| CR 0812 | spring-type | Sweden | 319,8 | 76,9 | 56,3 | 19 | 0,081150852 | -0,983870968 |
| CR 0814 | spring-type | Sweden | 232 | 21 | 41,9 | 1,3 | 0,002808411 | - |
| CR 0831 | spring-type | France | 424,9 | 120,5 | 62,2 | 21,4 | 0,097571662 | -0,343642612 |
| CR 0832 | spring-type | Hungary | 205,6 | 90,6 | 45,8 | 16,5 | 0,158753589 | -0,388888889 |
| CR 0842 | spring-type | Germany | 380 | 93 | 66 | 19,5 | 0,072308612 | -0,782051282 |
| CR 0844 | spring-type | Canada | 285,8 | 56,7 | 55,8 | 13,4 | 0,047642159 | -0,216463415 |
| CR 0861 | spring-type | Germany | 319,8 | 67,1 | 62,9 | 12,3 | 0,041029718 | -0,75 |
| CR 0881 | spring-type | Canada | 305 | 24 | 46,2 | 1,6 | 0,002725144 | -0,376068376 |
| CR 0882 | spring-type | Sweden | 332,3 | 61,7 | 53,6 | 11 | 0,038105066 | -0,35042735 |
| CR 0883 | spring-type | Canada | 475,75 | 82,5 | 65,75 | 18 | 0,047473571 | -0,24829932 |
| CR 0889 | spring-type | Australia | 401,3 | 162 | 50 | 22,9 | 0,18488911 | -1,024193548 |
| CR 0891 | spring-type | Germany | 437 | 40 | 54 | 11 | 0,018645648 | -0,252777778 |
| CR 0899 | spring-type | x | 392 | 44 | 60,5 | 8,25 | 0,015306122 | -1,043478261 |
| CR 0900 | spring-type | Germany | 375,275 | 77,4 | 58 | 15,1 | 0,053695795 | -0,384 |
| CR 0955 | spring-type | Germany | 447 | 36 | 61,6 | 9,7 | 0,012681949 | - |
| CR 0992 | spring-type | Sweden | 522 | 14 | 63,9 | 1,7 | 0,000713519 | -0,254658385 |
| CR 0993 | spring-type | Denmark | 354 | 116 | 56 | 11 | 0,064366425 | -0,418719212 |
| CR 0994 | spring-type | Sweden | 218 | 16 | 51,3 | 1,6 | 0,002289107 | -0,435779817 |
| CR 1011 | spring-type | Canada | 313 | 29 | 52,25 | 1,8 | 0,003191831 | -0,263975155 |
| CR 1014 | spring-type | Canada | 361,4 | 71 | 57,4 | 10,4 | 0,035595217 | -0,308823529 |
| CR 1016 | spring-type | Germany | 344 | 32 | 47 | 2 | 0,003958436 | -0,13015873 |
| CR 1020 | spring-type | Sweden | 365 | 34 | 55,7 | 3,6 | 0,006020511 | -0,192307692 |
| CR 1023 | spring-type | France | 363 | 145 | 56,3 | 26,6 | 0,188727253 | -0,40060241 |
| CR 1025 | spring-type | Canada | 306 | 60,5 | 64 | 13,3 | 0,041087112 | -0,528846154 |
| CR 1027 | spring-type | Canada | 222,5 | 43,6 | 54,7 | 13,4 | 0,048003615 | -0,411764706 |
| CR 1029 | spring-type | Netherlands | 287,4 | 135 | 56,3 | 23,9 | 0,199405214 | -0,368995633 |
| CR 1030 | spring-type | x | 210 | 13,25 | 37 | 1 | 0,001705277 | -0,26056338 |
| CR 1032 | spring-type | Canada | 255,3 | 123,8 | 54,9 | 28,3 | 0,249967715 | -0,459854015 |
| CR 1050 | spring-type | Germany | 332 | 57,9 | 60 | 17,2 | 0,049993976 | -0,335766423 |
| CR 1051 | spring-type | Australia | 342 | 32 | 54,8 | 2,8 | 0,004780808 | -0,488439306 |
| CR 1052 | spring-type | x | 323,6 | 180,6 | 56,6 | 27,3 | 0,269187847 | -0,631205674 |
| CR 1053 | spring-type | Australia | 423,2 | 149,2 | 51 | 28,8 | 0,19908818 | -0,397297297 |
| CR 1054 | spring-type | Canada | 382 | 46 | 59,8 | 4,4 | 0,00886025 | -0,516853933 |
| CR 1055 c | spring-type | x | 485,5 | 260,6 | 70,75 | 34,4 | 0,260985979 | -0,573275862 |
| CR 1056 | spring-type | Denmark | 317,5 | 22,1 | 49 | 1,8 | 0,002556966 | -0,368421053 |
| CR 1099 | spring-type | Sweden | 693 | 74 | 53,2 | 8,6 | 0,017261769 | -0,285185185 |
| CR 1100 | spring-type | Sweden | 382 | 40 | 46,7 | 4 | 0,008968912 | -0,364864865 |
| CR 1101 | spring-type | Sweden | 334 | 91 | 51,2 | 12,2 | 0,064920939 | -0,238636364 |
| CR 1170 | spring-type | Czech Republic | 283,4 | 139,2 | 55,1 | 32,8 | 0,292389407 | -0,183673469 |
| CR 1171 | spring-type | Ukraine | 374,7 | 127,7 | 51,3 | 21,1 | 0,140175558 | -0,299528302 |
| CR 1174 | spring-type | Russia | 372 | 43 | 62,1 | 8,1 | 0,015077139 | -0,326829268 |
| CR 1855 | spring-type | Ukraine | 483 | 33 | 59,3 | 12,6 | 0,014517193 | -0,291836735 |
| CR 1861 | spring-type | x | 299,5 | 33,9 | 51,5 | 5,8 | 0,012747459 | - |
| CR 1883 | spring-type | Romania | 387,8 | 200,6 | 55,25 | 29,3 | 0,274320625 | -0,266129032 |
| CR 1885 | spring-type | Russia | 295,5 | 63,1 | 57,75 | 9,2 | 0,034017917 | -0,587628866 |
| CR 1886 | spring-type | Russia | 305,9 | 122,9 | 58,9 | 22,4 | 0,152793588 | -0,306962025 |
| CR 1887 | spring-type | Italy | 420 | 23 | 62,3 | 3,5 | 0,003076512 | -0,13740458 |
| CR 2005 c | spring-type | Canada | 345,9 | 84,9 | 58,5 | 21,3 | 0,089367759 | -0,452941176 |
| CR 2012 | spring-type | Sweden | 381 | 30,5 | 50,5 | 2,75 | 0,004359294 | -0,181564246 |
| CR 2013 | spring-type | Sweden | 314,9 | 93,5 | 58,3 | 13,4 | 0,068245684 | -0,37745098 |
| CR 2014 | spring-type | Sweden | 279,2 | 55,7 | 64,2 | 10,8 | 0,033560507 | -0,289156627 |
| CR 2015 | spring-type | Sweden | 280,8 | 37,6 | 58 | 6,4 | 0,014775518 | -0,540697674 |
| CR 2214 | spring-type | Sweden | 430 | 236 | 55,3 | 25,9 | 0,257050339 | -0,388888889 |
| CR 2250 | spring-type | Sweden | 362 | 87 | 42 | 8,8 | 0,05035517 | -0,350515464 |
| CR 2256 | spring-type | x | 719 | 32 | 51,8 | 1,9 | 0,001632469 | -0,275390625 |
| CR 2257 | spring-type | Poland | 105,1 | 36,7 | 39,5 | 5,6 | 0,049505594 | -1,24137931 |
| CR 2258 | spring-type | x | 283 | 86 | 58,9 | 26 | 0,134143634 | -0,341584158 |
| CR 2259 | spring-type | x | 497 | 310 | 54 | 13,5 | 0,155935614 | -0,216738197 |
| CR 2260 | spring-type | x | 586 | 43 | 55,6 | 5,9 | 0,007786603 | -0,364197531 |
| CR 2262 | spring-type | Italy | 318 | 12 | 59,9 | 0,83 | 0,000522884 | -0,43373494 |
| CR 2263 | spring-type | France | 466 | 45 | 63,7 | 3,1 | 0,00469947 | -0,34 |
| CR 2264 | spring-type | China | 835 | 23 | 54,5 | 4,8 | 0,002425974 | -0,282978723 |
| CR 2267 | spring-type | Taiwan | 329 | 297,7 | 60,5 | 47,2 | 0,705942877 | 0,291666667 |
| CR 2271 | spring-type | Italy | 361 | 135 | 53,8 | 32,2 | 0,223820655 | -0,365384615 |
| CR 2272 c | spring-type | Pakistan | 347,6 | 303,8 | 67,1 | 47,2 | 0,61479097 | -0,238095238 |
| CR 2273 | spring-type | Korea | 520 | 56 | 73,8 | 6,8 | 0,009922868 | -0,081632653 |
| CR 2274 | spring-type | Korea | 309,6 | 234,6 | 55,3 | 35,8 | 0,490551888 | -0,283088235 |
| CR 2275 | spring-type | Korea | 479,5 | 155,5 | 61,4 | 27,25 | 0,143926219 | -0,383435583 |
| CR 2276 | spring-type | Korea | 366,3 | 172,9 | 62 | 37 | 0,281687846 | -0,171532847 |
| CR 2277 c | spring-type | Korea | 219,8 | 69,25 | 46 | 17,3 | 0,118489635 | -0,369918699 |
| CR 2278 | spring-type | Korea | 372 | 140 | 42,4 | 24 | 0,213024954 | -0,25877193 |
| CR 2279 | spring-type | Korea | 564 | 456 | 67,3 | 50,1 | 0,601877905 | 0,041666667 |
| CR 2280 | spring-type | Vietnam | 671 | 516 | 57,9 | 53 | 0,703921917 | 0,540540541 |
| CR 2281 | spring-type | Korea | 261,7 | 73 | 51,8 | 13,9 | 0,074852133 | -0,290697674 |
| CR 2282 | spring-type | Korea | 345 | 167 | 58,9 | 35 | 0,28764056 | -0,158450704 |
| CR 2283 | spring-type | Sweden | 293,1 | 62,2 | 52,75 | 9,75 | 0,039224437 | -0,444 |
| CR 2284 c | spring-type | China | 345,9 | 191,5 | 52 | 37,9 | 0,403509796 | -0,190647482 |
| CR 2285 | spring-type | China | 390,5 | 399,1 | 63 | 54,25 | 0,880075402 | -0,42 |
| CR 2321 | spring-type | x | 433 | 78 | 59,5 | 6,2 | 0,018770742 | -0,281553398 |
| CR 2411 | spring-type | Korea | 492,5 | 246,2 | 65 | 45,2 | 0,34762171 | -0,095588235 |
| CR 2425 | spring-type | Germany | 359 | 63 | 60,8 | 4,8 | 0,013854274 | -0,064220183 |
| CR 2614 | spring-type | Russia | 443 | 123 | 58,2 | 6,6 | 0,031486351 | -0,14 |
| CR 2626 | spring-type | x | 754 | 200 | 66,8 | 7,7 | 0,030575454 | -0,1953125 |
| CR 2633 | spring-type | Canada | 498 | 32 | 60 | 7,2 | 0,007710843 | -0,325431034 |
| CR 2674 | spring-type | Czech Republic | 451 | 237 | 55,3 | 27,7 | 0,26322458 | -0,2 |
| CR 2698 | spring-type | x | 317,2 | 105,4 | 53,7 | 19,8 | 0,122517559 | -0,888888889 |
| CR 3017 | spring-type | x | 377,95 | 117,2 | 57,9 | 21,3 | 0,114076005 | -0,3359375 |
| CR 3020 | spring-type | Germany | 587 | 17 | 59,6 | 1,3 | 0,000631696 | -0,36875 |
| CR 3021 | spring-type | Sweden | 412 | 56 | 64,3 | 7,4 | 0,015642694 | -0,25 |
| CR 3023 | spring-type | Germany | 275,1 | 218 | 59,7 | 36,5 | 0,484489575 | -0,270833333 |
| CR 3033 | spring-type | x | 465 | 22 | 64,3 | 6 | 0,00441479 | -0,177083333 |
| CR 3034 | spring-type | Germany | 462 | 115 | 57,8 | 4,7 | 0,020240717 | -0,308743169 |
| CR 3035 c | spring-type | Germany | 294 | 18 | 48,7 | 0,3 | 0,000377153 | -0,289719626 |
| CR 3037 | spring-type | Germany | 345 | 53 | 48,5 | 2,1 | 0,006651726 | -0,46 |
| CR 3039 | spring-type | Russia | 399,6 | 90 | 55,75 | 8,8 | 0,035551246 | -0,348258706 |
| CR 3040 | spring-type | Germany | 340 | 26 | 61,7 | 5,2 | 0,006444847 | -0,267241379 |
| CR 3046 | spring-type | France | 429,7 | 100,2 | 71,2 | 14,3 | 0,046833694 | -0,353535354 |
| CR 3050 | spring-type | Germany | 462 | 44 | 62 | 11,3 | 0,017357911 | -0,363333333 |
| CR 3052 | spring-type | Romania | 361,5 | 181,7 | 58 | 24,9 | 0,215783374 | -0,300947867 |
| CR 3062 | spring-type | Germany | 478 | 29 | 65 | 1,8 | 0,001680077 | -0,387931034 |
| CR 3072 | spring-type | Germany | 324 | 49,75 | 52,5 | 5,7 | 0,016671076 | -0,40625 |
| CR 3076 | spring-type | Germany | 664 | 21 | 56,4 | 1,25 | 0,000700942 | -0,289308176 |
| CR 3077 | spring-type | Australia | 412 | 21 | 58 | 4 | 0,003515233 | -0,165745856 |
| CR 3080 | spring-type | Germany | 607 | 66 | 63,7 | 6,2 | 0,010582968 | -0,175438596 |
| CR 3085 | spring-type | Germany | 366 | 34 | 51,3 | 3 | 0,005432525 | -0,357142857 |
| CR 3086 | spring-type | Sweden | 384 | 22 | 60 | 3 | 0,002864583 | -0,35078534 |
| CR 3090 | spring-type | Sweden | 432 | 78 | 48 | 15 | 0,056423611 | -0,18537415 |
| CR 3095 | spring-type | Germany | 968 | 92 | 54,9 | 7 | 0,012118201 | -0,262032086 |
| CR 3096 | spring-type | Australia | 389 | 56 | 54,2 | 13,7 | 0,036388127 | -0,337349398 |
| CR 3100 | spring-type | Sweden | 434 | 20 | 55 | 2 | 0,001675744 | -0,183673469 |
| CR 3102 | spring-type | Germany | 393 | 66 | 60,2 | 11,2 | 0,031244452 | -0,233576642 |
| CR 3105 | spring-type | Germany | 239 | 164 | 51 | 28 | 0,37673312 | -0,369565217 |
| CR 3111 | spring-type | Germany | 258,1 | 51 | 60,8 | 9,9 | 0,032174647 | -0,28 |
| CR 3117 | spring-type | x | 330 | 32 | 52 | 3 | 0,005594406 | -0,330357143 |
| CR 3122 | spring-type | x | 283 | 86,3 | 59,2 | 14,25 | 0,073403627 | -0,277385159 |
| CR 3123 c | spring-type | Germany | 523 | 87 | 59,9 | 6 | 0,01666257 | -0,344311377 |
| CR 3133 c | spring-type | Canada | 293 | 35 | 54,9 | 6,6 | 0,014360581 | -0,265243902 |
| CR 3135 c | spring-type | Canada | 830 | 34 | 59,2 | 3 | 0,002075871 | -0,31875 |
| CR 3141 | spring-type | Canada | 388 | 45 | 61,3 | 5 | 0,009459982 | -0,299295775 |
| CR 3142 | spring-type | Germany | 325,4 | 266,4 | 56,2 | 41,2 | 0,600174546 | -0,248366013 |
| CR 3143 c | spring-type | Germany | 513 | 79 | 67 | 3 | 0,006895348 | -0,017241379 |
| CR 3152 | spring-type | Canada | 422 | 67 | 56,9 | 2,7 | 0,007533796 | -0,21474359 |
| CR 3153 c | spring-type | Canada | 346 | 37 | 47,8 | 1 | 0,002237164 | -0,31875 |
| CR 3156 | spring-type | Czech Republic | 450 | 71 | 58 | 4 | 0,010881226 | -0,247292419 |
| CR 3162 | spring-type | Germany | 350 | 34 | 55,2 | 11,2 | 0,019710145 | -0,503846154 |
| CR 3165 | spring-type | x | 337 | 49 | 54 | 4 | 0,010770414 | -0,222513089 |
| CR 3166 | spring-type | Finland | 388 | 81 | 56 | 12 | 0,044734904 | - |
| CR 3167 | spring-type | Germany | 377 | 76 | 54,8 | 5,5 | 0,020232725 | -0,378453039 |
| CR 3170 | spring-type | Germany | 421 | 28 | 51 | 4 | 0,005216338 | -0,160087719 |
| CR 3182 | spring-type | Denmark | 490 | 17 | 54,2 | 1,3 | 0,000832141 | -0,46641791 |
| CR 3185 | spring-type | Germany | 423 | 109 | 58,5 | 9,6 | 0,042286476 | -0,224264706 |
| CR 3186 | spring-type | Germany | 264,7 | 144 | 51,3 | 24,4 | 0,258750389 | -0,297687861 |
| CR 3189 | spring-type | Poland | 654 | 84 | 55,6 | 11,3 | 0,026103888 | -0,224242424 |
| CR 3192 | spring-type | Germany | 255 | 16 | 53,4 | 1,6 | 0,001880003 | -0,47752809 |
| CR 3194 | spring-type | Germany | 345 | 37 | 59,6 | 1,1 | 0,001979379 | -0,276041667 |
| CR 3195 c | spring-type | Australia | 378 | 107 | 49,2 | 17,7 | 0,101835721 | 0,020833333 |
| CR 3197 | spring-type | Marocco | 427 | 54 | 57 | 5 | 0,011093307 | -0,290948276 |
| CR 3198 | spring-type | Canada | 329,4 | 121 | 61,75 | 20,9 | 0,124328616 | -0,276824034 |
| CR 3205 | spring-type | Germany | 378 | 21 | 47,2 | 1,5 | 0,001765537 | -0,266990291 |
| CR 3207 | spring-type | Germany | 332,125 | 83,9 | 54,7 | 38 | 0,175491733 | -0,39453125 |
| CR 3210 | spring-type | Canada | 298 | 44 | 53,7 | 2,1 | 0,005774062 | -0,158844765 |
| CR 3212 | spring-type | Germany | 399 | 98 | 60,7 | 10,7 | 0,043296049 | -0,452380952 |
| CR 3213 | spring-type | Germany | 400 | 25 | 58,4 | 2,9 | 0,003103596 | -0,403061224 |
| CR 3226 c | spring-type | Germany | 401 | 76 | 57,9 | 2,5 | 0,008183341 | -0,235849057 |
| CR 3227 | spring-type | Germany | 419,7 | 106,4 | 60 | 19,8 | 0,083659757 | -0,273869347 |
| CR 3228 c | spring-type | Germany | 298 | 55 | 54,4 | 8 | 0,027141729 | -0,608695652 |
| CR 3229 | spring-type | Germany | 378 | 37 | 50,5 | 1,8 | 0,00348892 | -0,237980769 |
| CR 3231 c | spring-type | Germany | 316 | 124 | 60,5 | 12 | 0,077832409 | -0,450819672 |
| CR 3239 | spring-type | Germany | 386,3 | 77 | 60,6 | 41,3 | 0,135844933 | -0,25 |
| CR 3243 | spring-type | Canada | 495 | 63 | 60,6 | 8,5 | 0,017851785 | -0,308383234 |
| CR 3245 | spring-type | Australia | 432 | 52 | 64,3 | 5,4 | 0,010108865 | -0,406666667 |
| CR 3246 | spring-type | x | 464 | 30 | 55 | 3 | 0,003526646 | -0,225531915 |
| CR 3247 | spring-type | Australia | 466 | 31 | 55,6 | 3 | 0,003589403 | -0,29950495 |
| CR 3248 | spring-type | Japan | 389 | 20 | 55 | 2 | 0,001869596 | -0,270428016 |
| CR 3249 | spring-type | x | 501 | 86 | 63,3 | 8,8 | 0,023863805 | -0,521428571 |
| CR 3250 | spring-type | Poland | 283 | 24 | 50 | 2 | 0,003392226 | -0,398026316 |
| CR 3251 | spring-type | x | 436 | 35 | 57 | 2 | 0,002816675 | -0,131436314 |
| CR 3254 | spring-type | China | 443 | 65 | 46 | 3 | 0,009569143 | 0,121052632 |
| CR 3255 | spring-type | Germany | 436 | 34 | 58 | 2 | 0,002689022 | -0,096625767 |
| CR 3256 | spring-type | Germany | 356 | 54 | 57 | 9 | 0,023950325 | -0,188741722 |
| CR 3257 | spring-type | Germany | 422 | 34 | 63 | 3 | 0,003836606 | -0,256997455 |
| CR 3281 | spring-type | Germany | 465 | 64 | 62 | 7 | 0,015539369 | -0,217391304 |
| CR 3282 | spring-type | Netherlands | 340,7 | 106,5 | 62,4 | 28,25 | 0,141517887 | -0,367816092 |
| CR 3283 | spring-type | Sweden | 361,6 | 22,7 | 53,3 | 2,2 | 0,002591152 | -0,336363636 |
| CR 3284 | spring-type | Canada | 371 | 89 | 54,9 | 6,3 | 0,027528611 | -0,25625 |
| CR 3289 | spring-type | x | 421 | 145 | 59,6 | 25,3 | 0,146204307 | -0,413705584 |
| CR 3290 | spring-type | Sweden | 376 | 54 | 60,9 | 9,4 | 0,022167488 | -0,528846154 |
| CR 3291 | spring-type | Germany | 332,4 | 177 | 50,3 | 29,7 | 0,31441316 | -0,306629834 |
| CR 3292 | spring-type | Germany | 302 | 19 | 35,3 | 0,1 | 0,000178226 | -0,299107143 |
| CR 3300 | spring-type | Germany | 345 | 27 | 54,3 | 5,3 | 0,007638722 | -0,449275362 |
| CR 3301 | spring-type | Sweden | 502 | 71 | 61,5 | 13,9 | 0,031966443 | -0,37037037 |
| CR 3401 | spring-type | Germany | 367 | 28 | 61 | 7 | 0,008755081 | -0,113861386 |
| CR 3402 | spring-type | Denmark | 400 | 78 | 56 | 6 | 0,020892857 | -0,142857143 |
| CR 3403 | spring-type | Germany | 334 | 46 | 59 | 7 | 0,016340201 | -0,196202532 |
| CR 3404 | spring-type | Germany | 378 | 23 | 60 | 3 | 0,003042328 | -0,169491525 |
| CR 3423 | spring-type | Cuba | 298 | 51 | 39 | 9,4 | 0,041249355 | -0,435114504 |
| CR 3430 | spring-type | Germany | 421 | 25 | 47 | 2 | 0,002526912 | -0,202531646 |
| CR 3431 | spring-type | Denmark | 378 | 43 | 56 | 18 | 0,036564626 | -0,314606742 |
| CR 3432 | spring-type | Germany | 512 | 40 | 46 | 6 | 0,010190217 | -0,536111111 |
| CR 3433 | spring-type | Germany | 402 | 34 | 58 | 7 | 0,010207583 | -0,364485981 |
| CR 3481 | spring-type | Netherlands | 337 | 44 | 55 | 4 | 0,009495549 | -0,389925373 |
| CR 3500 | spring-type | Germany | 265 | 21 | 50 | 1 | 0,001584906 | 0,518867925 |
| CR 3501 | spring-type | Germany | 347 | 14 | 50 | 1 | 0,000806916 | 0,023529412 |
| CR 3502 | spring-type | Germany | 395 | 19 | 53 | 3 | 0,002722713 | -0,210743802 |
| CR 3503 | spring-type | Germany | 525 | 22 | 51 | 2 | 0,001643324 | -0,297687861 |
| CR 3504 | spring-type | Germany | 471 | 55 | 51 | 3 | 0,00686899 | -0,402116402 |
| CR 3505 | spring-type | Germany | 490 | 55 | 60 | 2 | 0,003741497 | -0,5 |
| Zhongshuang | spring-type | China | 241 | 146 | 54,6 | 18,6 | 0,206374538 | - |
| Qingyou | spring-type | China | 366 | 173 | 63,9 | 8,4 | 0,062136022 | - |
