# Supplementary material for: Identification of Rapeseed (Brassica napus) Cultivars With a High Tolerance to Boron-Deficient Conditions
Source: Front Plant Sci. 2018 Aug 7;9:1142. doi: 10.3389/fpls.2018.01142 (PMC6091279; doi:10.3389/fpls.2018.01142)

**Supplementary_Data_Sheet_S2:** **Diagram showing the B supply regime in the screen. (A)** Diagram showing the supplied levels of B in the soil substrates used for B-dependent growth analyses of B-efficient and B-inefficient genotypes. B was supplied as boric acid dissolved in water and supplied by watering pots and plants from the bottom and from above. Yellow area indicates deficient (B 0), green area adequate (B 1), and orange area excessive (B 2) B supply. Red asterisks indicate time points of B supply. Plant samples were harvested at 5-, 10-, 15-, 20-, and 25-DAG. **(B)** Exemplary pictures of plants from B-efficient (E) and B-inefficient (IE) genotypes at 10-DAG growing on different B supply levels (B 0, B 1, B 2).


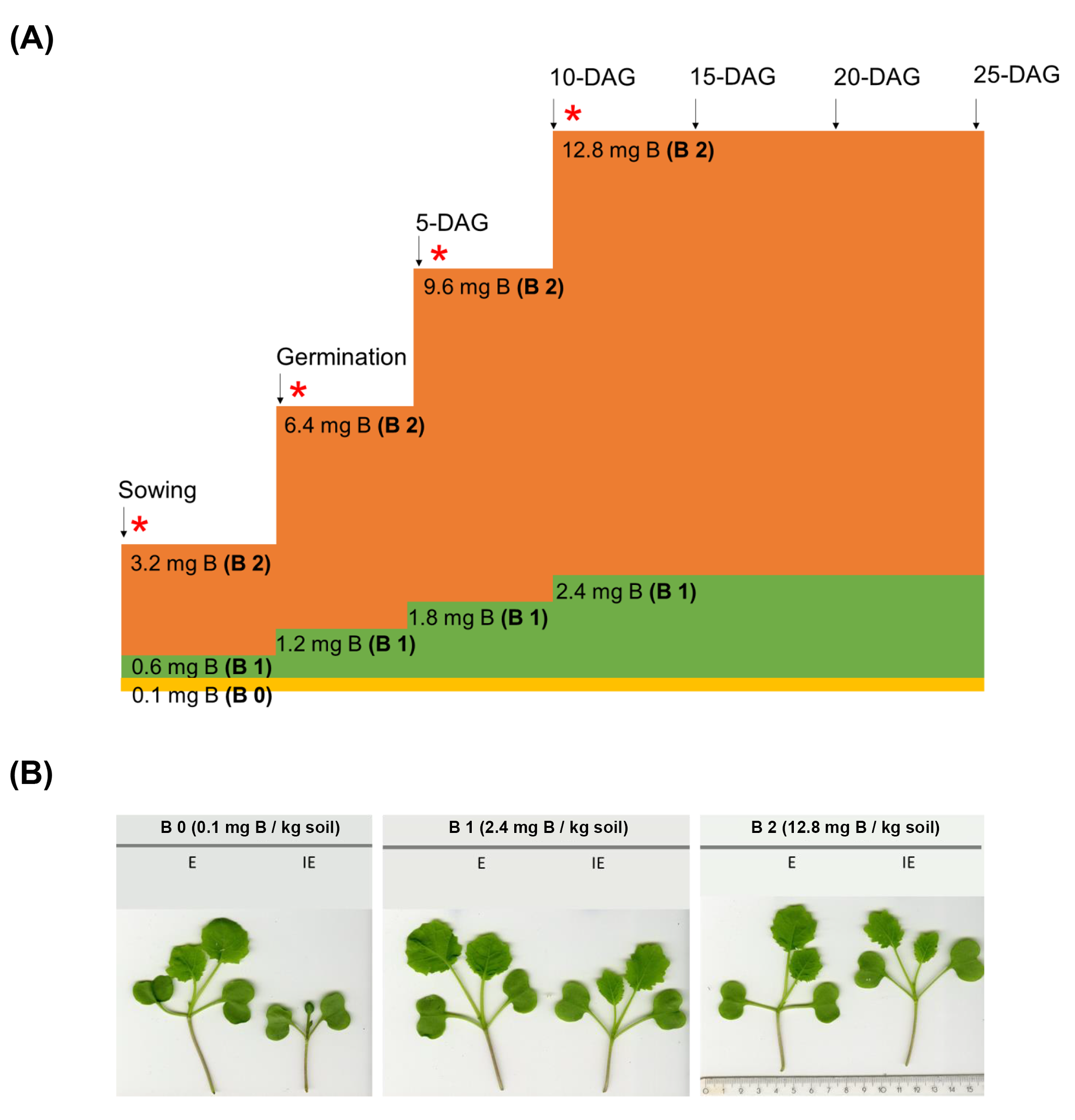

Supplement: Supplementary file 2 [file Data_Sheet_2.docx]
