# Supplementary material for: Identification of Rapeseed (Brassica napus) Cultivars With a High Tolerance to Boron-Deficient Conditions
Source: Front Plant Sci. 2018 Aug 7;9:1142. doi: 10.3389/fpls.2018.01142 (PMC6091279; doi:10.3389/fpls.2018.01142)
Supplement: Supplementary file 3 [file Data_Sheet_3.docx]

**Supplementary_Data_Sheet_S3: Thirteen *Brassica napus* genotypes, which have been analysed by image analyses by the automated LemnaTec phenotyping platform.**

| **Accession code** | **Life form** | **Boron Efficiency Index** |
| --- | --- | --- |
| *CR2267* | spring-type | 0.7059 |
| *CR2262* | spring-type | 0.0005 |
| *CR3153* | spring-type | 0.0022 |
| *CR2285* | spring-type | 0.8801 |
| *CR2280* | spring-type | 0.7039 |
| *CR3061* | winter-type | 0.3487 |
| *Darmor-PBY018* | winter-type | not determined |
| *CR0160* | winter-type | 0.0054 |
| *CR0294* | winter-type | 0.0278 |
| *CR3261* | winter-type | 0.0333 |
| *CR3126* | winter-type | 0.5675 |
| *CR3219* | winter-type | 0.6264 |
| *CR3019* | winter-type | 0.6264 |
