# Supplementary material for: Identification of Rapeseed (Brassica napus) Cultivars With a High Tolerance to Boron-Deficient Conditions
Source: Front Plant Sci. 2018 Aug 7;9:1142. doi: 10.3389/fpls.2018.01142 (PMC6091279; doi:10.3389/fpls.2018.01142)
Supplement: Supplementary file 4 [file Data_Sheet_4.docx]

**Supplementary_Data_Sheet_S4:** **Zerosoil-substrate nutrient concentrations**. Zerosoil-substrate nutrient concentrations have been analysed. Zerosoil-substrate has been separated form *Brassica napus* roots after the growth experiment in the automated phenotyping platform. Zerosoil-substrate from three soil columns has been homogenously mixed and 300 g have been used for analyses. Each value represents the average ±SD of three independent measurements, each consisting of a pool of three soil pots.

| **Nutrient/Soil** | **Element** | **Boron Fertilization Level** | | |
| --- | --- | --- | --- | --- |
|  |  | **-** | **+** | **++** |
| mg/kg | **B** | **0.143 ±0.02** | **1±0.09** | **33.62±1.62** |
| mg/100g | P | 32.23 ±6.49 | 28.03±1.54 | 31.9±1.59 |
| mg/100g | K | 36.67 ±13.59 | 17.3±3.65 | 19.13±2.65 |
| mg/100g | Mg | 61.17 ±3.67 | 54.5±2.18 | 58.9±1.39 |
| mg/kg | Cu | 3.53 ±0.06 | 3.57±0.55 | 4.2±0.26 |
| mg/100g | Fe | 31.93 ±1.04 | 31.33±4.59 | 37.13±2 |
| mg/kg | Mn | 15.33 ±0.58 | 15.67±2.31 | 17.67±2.31 |
| mg/kg | Zn | 6.83 ±0.49 | 7.27±0,55 | 8.27±0.32 |
| mg/100g | Ca | 555.33 ±18.59 | 533±3.61 | 549.33±7.24 |
| mg/100g | SO_4_ | 3.4 ±1.54 | 1.6±0.3 | 1.97±0.25 |
