# Supplementary material for: Identification of Rapeseed (Brassica napus) Cultivars With a High Tolerance to Boron-Deficient Conditions
Source: Front Plant Sci. 2018 Aug 7;9:1142. doi: 10.3389/fpls.2018.01142 (PMC6091279; doi:10.3389/fpls.2018.01142)
Supplement: Supplementary file 5 [file Data_Sheet_5.docx]

**Supplementary_Data_Sheet_S5:** Twelve relevant biomass-related, architectural and color-related traits extracted from top-view (TOP) images taken in the IPK phenotyping facility by visible-light (VIS) or fluorescence (FLUOR) cameras. (Selection) indicates whether the parameter was used for automated image pre-processing and segmentation in the Integrated Analysis Plattform (IAP, Klukas et al., 2014).

| **LemnaTec trait** | **imaging modality** | **imaging view** | **trait type** | **Selection** |
| --- | --- | --- | --- | --- |
| hsv h red2green (color-related trait based on visible-light top view) | VIS | TOP | color | YES |
| hsv h mean (color-related trait based on visible-light top view) | VIS | TOP | color | YES |
| intensity mean (color-related trait based on fluorescence top view) | FLUOR | TOP | intensity | YES |
| rgb green mean (color-related trait based on visible-light top view) | VIS | TOP | color | YES |
| rgb red mean (color-related trait based on visible-light top view) | VIS | TOP | color | YES |
| lab a mean (color-related trait based on visible-light top view) | VIS | TOP | color | YES |
| hsv h yellow2green (color-related trait based on visible-light top view) | VIS | TOP | color | YES |
| hull compactness 16 (geometry trait based on visible-light top view) | VIS | TOP | architectural | YES |
| compactness 16 (geometry trait based on visible-light top view) | VIS | TOP | architectural | YES |
| border length (geometry trait based on visible-light top view) [px] | VIS | TOP | architectural | YES |
| area (geometry trait based on visible-light top view) [px^2] | VIS | TOP | biomass-related | YES |
| hull fillgrade (geometry trait based on fluorescence top view) [%] | FLUOR | TOP | architectural | YES |
