# Supplementary material for: Identification of Rapeseed (Brassica napus) Cultivars With a High Tolerance to Boron-Deficient Conditions
Source: Front Plant Sci. 2018 Aug 7;9:1142. doi: 10.3389/fpls.2018.01142 (PMC6091279; doi:10.3389/fpls.2018.01142)

**Supplementary_Data_Sheet_S6: Screening analysis of winter-type rapeseed (*Brassica napus*) genotypes grown under adequate (B1) or deficient (B0) boron conditions on zerosoil-substrate.** Boxplots of the Boron Efficiency Index (BEIs) **(A)** and boron efficiency growth parameters (length of leaf 1 (L1), length of the hypocotyl and the dry weight) **(B)** from 356 winter-type rapeseed genotypes. Crosses inside boxes show average values, horizontal lines within boxes mark the median value. Each boxplot consists of the parameter average values from n ≥ 10 plants.


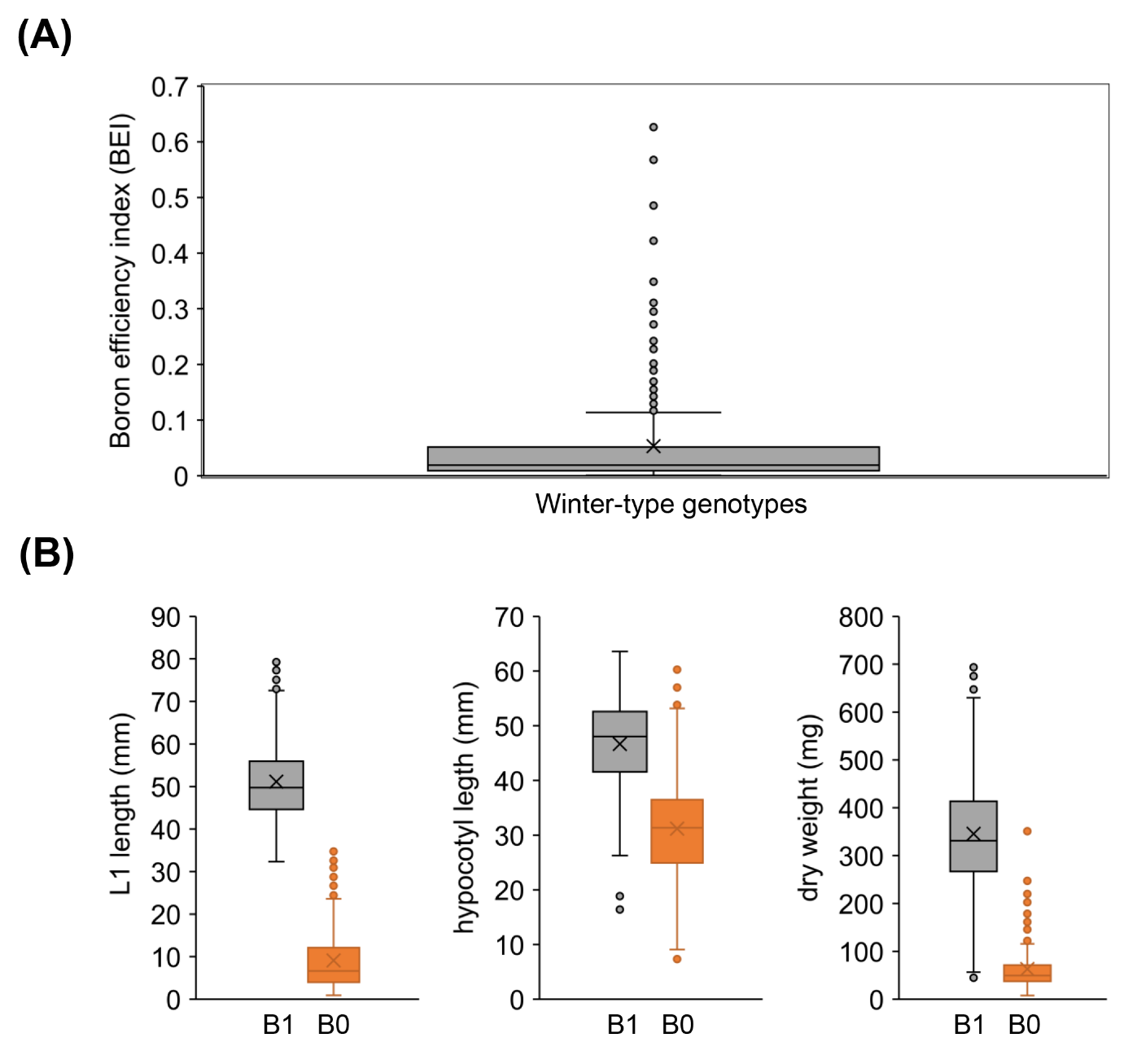

Supplement: Supplementary file 6 [file Data_Sheet_6.docx]
