# Supplementary material for: Identification of Rapeseed (Brassica napus) Cultivars With a High Tolerance to Boron-Deficient Conditions
Source: Front Plant Sci. 2018 Aug 7;9:1142. doi: 10.3389/fpls.2018.01142 (PMC6091279; doi:10.3389/fpls.2018.01142)

**Supplementary_Data_Sheet_S7:** **Validation of the image analysis pipeline.** Correlation analysis between *Brassica napus* shoot fresh weight after harvest and the imaging based parameter top view projected leaf area (in pixels, extracted from images of the last imaging round before harvest) reveals a significant (P<0.001) positive correlation with R=0.928.


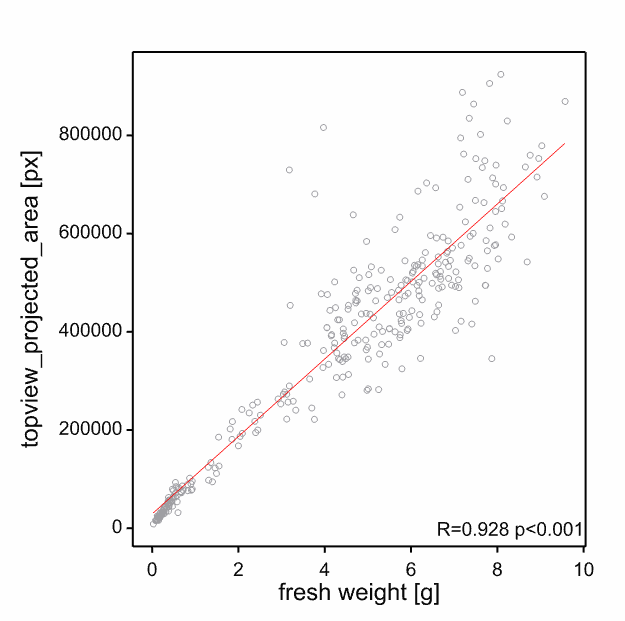

Supplement: Supplementary file 7 [file Data_Sheet_7.docx]
