# Supplementary material for: Identification of Rapeseed (Brassica napus) Cultivars With a High Tolerance to Boron-Deficient Conditions
Source: Front Plant Sci. 2018 Aug 7;9:1142. doi: 10.3389/fpls.2018.01142 (PMC6091279; doi:10.3389/fpls.2018.01142)

**Supplementary_Data_Sheet_S8: (A)** Boxplot of the ‘border-length’ parameter for selected *Brassica napus* genotypes at day 10 after sowing determined from RGB top-view images. The ‘border-length’ parameter of *B. napus* plants was determined by top-view visible-light imaging 10 days after sowing (10 DAS) and is given in pixels [px]. Border-length is a geometry-based trait which represents the pixel count between background and foreground (plant perimeter) and is therefore a suitable proxy for the plants’ biomass. Border-length was determined of indicated *B. napus* genotypes (n = 8) grown under B-deficient (-; ≈ 0.143 mg B (kg soil)^-1^), B-sufficient (+; ≈ 1 mg B (kg soil)^-1^) or B-surplus (++; ≈ 33.62 mg B (kg soil)^-1^) conditions. (*CR2267* = E, *CR2280* = E2, *CR2262* = IE, *CR3153* = IE2, *Darmor* = D). Letters indicate significance at the 0.05 level according to Anova with post-hoc Bonferroni test. **(B)** Correlation analysis between the two image-analysis-based parameters ‘top view projected area’ and ‘border-length’ for all *B. napus* genotypes in the phenotyping experiment and all imaging days after sowing (4-13 DAS). Both parameters have been extracted from RGB top-view visible-light images and are given in pixels [px]. A significant positive correlation was found with R=0.95 at the p<0.001 level.

**(A)**

**(B)**


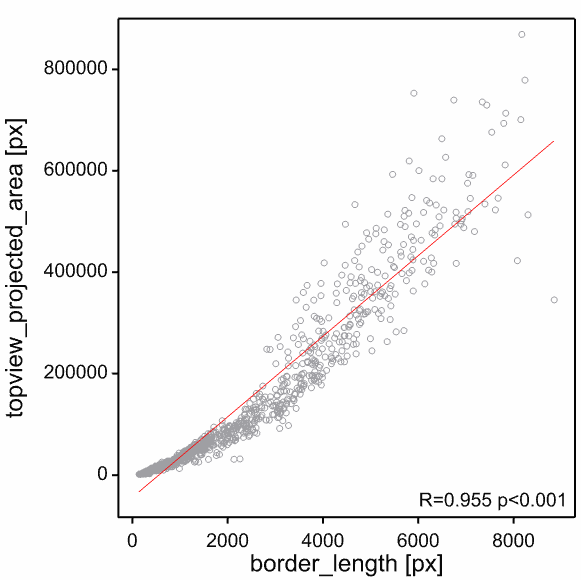

Supplement: Supplementary file 8 [file Data_Sheet_8.docx]
