# Supplementary material for: Identification of Rapeseed (Brassica napus) Cultivars With a High Tolerance to Boron-Deficient Conditions
Source: Front Plant Sci. 2018 Aug 7;9:1142. doi: 10.3389/fpls.2018.01142 (PMC6091279; doi:10.3389/fpls.2018.01142)

**Supplementary_Data_Sheet_S9: Fresh weights and water content of *Brassica napus* genotypes grown in the LemnaTec assay. (A)** Fresh weight (FW) per plant of indicated *B. napus* genotypes grown under B-deficient (−), B-sufficient (+) or B-surplus (++) conditions. FW values are means ± SD from n = 8 plants. **(B)** water content of *B. napus* plants grown under B-deficient (−), B-sufficient (+) or B-surplus (++) conditions. Water values are means ± SD from n = 3 plants. Significance levels were tested using t-test (*** = p < 0.001, ** = p < 0.005, * = p < 0.05. Please note: water content for IE2 **(B)** under (−) is represented by only one replicate.


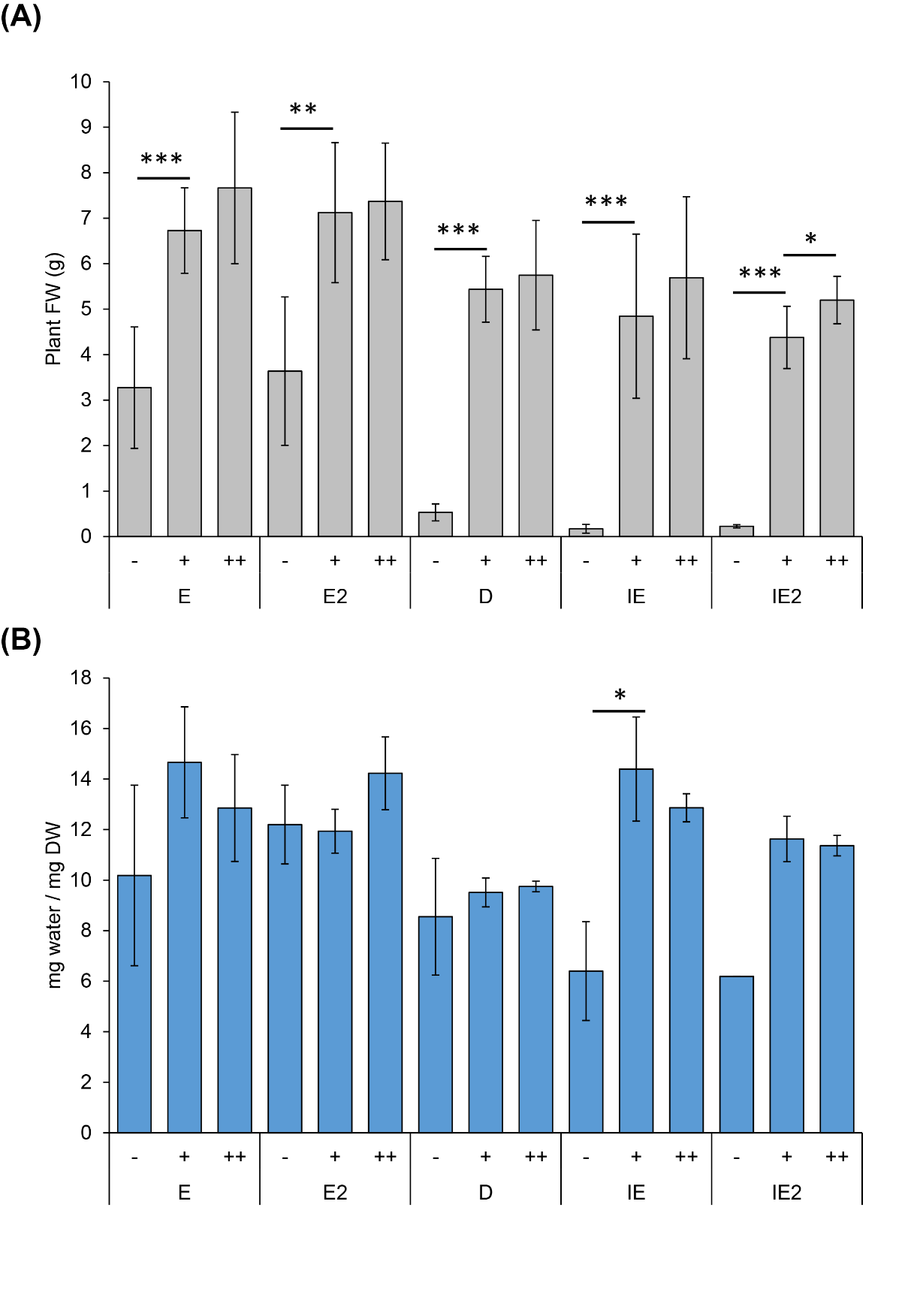

Supplement: Supplementary file 9 [file Data_Sheet_9.docx]
