# Supplementary material for: Identification of Rapeseed (Brassica napus) Cultivars With a High Tolerance to Boron-Deficient Conditions
Source: Front Plant Sci. 2018 Aug 7;9:1142. doi: 10.3389/fpls.2018.01142 (PMC6091279; doi:10.3389/fpls.2018.01142)
Supplement: Supplementary file 10 [file Data_Sheet_10.docx]

**Supplementary_Data_Sheet_S10:** **Element concentrations of different *Brassica napus* plant parts.** HR-ICP-MS-based element analysis of different aerial *B. napus* plant parts of two B deficiency tolerant (E, E2) and two B deficiency sensitive (IE, IE2) cultivars grown in an automated IPK phenotyping facility under B-deficient (−), B-sufficient (+) or B-surplus (++) conditions. Values represent concentrations of specific plant parts or organs (cot = cotyledon, L1 = first vegetative leaf, L3 = third vegetative leaf, RL = remaining leaves, stem = remaining stem tissue without leaves). Values show mean element concentrations in ng per mg dry weight (DW) from n = 3 biological replicates ± SE.

|  |  |  | **average concentration per part (ng / mg DW)** | | | | | | | | |
| --- | --- | --- | --- | --- | --- | --- | --- | --- | --- | --- | --- |
| **cultivar** | **boron condition** | **plant part** | **B** | **Ca** | **Cu** | **Fe** | **Mg** | **Mn** | **Na** | **S** | **Zn** |
|  |  |  |  |  |  |  |  |  |  |  |  |
| E | (-) | Cot | 8.02 | 64533.93 | 6.71 | 79.88 | 6876.30 | 93.92 | 10372.36 | 10220.77 | 39.56 |
|  |  | ± SE | 3.81 | 13297.51 | 3.71 | 16.66 | 513.28 | 17.14 | 2957.39 | 2172.78 | 1.79 |
| E | (-) | L1 | 5.62 | 45113.00 | 5.00 | 86.59 | 5529.23 | 110.18 | 8363.41 | 11839.84 | 42.10 |
|  |  | ± SE | 2.53 | 10616.09 | 1.49 | 21.81 | 769.44 | 21.16 | 3941.50 | 2246.37 | 8.95 |
| E | (-) | L3 | 4.03 | 31928.76 | 6.05 | 96.96 | 4721.65 | 104.49 | 8199.38 | 10011.43 | 61.95 |
|  |  | ± SE | 1.97 | 8830.12 | 1.74 | 21.06 | 729.34 | 31.87 | 3903.54 | 1677.54 | 13.66 |
| E | (-) | LR | 4.49 | 28031.05 | 6.38 | 88.70 | 4316.30 | 91.74 | 7769.12 | 10201.74 | 59.90 |
|  |  | ± SE | 1.17 | 6980.28 | 1.25 | 19.35 | 319.50 | 9.96 | 3663.94 | 929.72 | 10.33 |
| E | (-) | STEM | 6.19 | 13186.58 | 7.38 | 76.85 | 3376.27 | 43.72 | 6358.51 | 10546.30 | 73.97 |
|  |  | ± SE | 1.86 | 2754.02 | 1.46 | 9.41 | 265.68 | 2.28 | 3161.93 | 1205.74 | 9.16 |
| E | (+) | Cot | 34.77 | 28458.85 | 6.42 | 66.75 | 2481.17 | 126.99 | 1892.83 | 5452.62 | 29.22 |
|  |  | ± SE | 14.60 | 12921.91 | 6.82 | 45.85 | 649.81 | 62.06 | 565.13 | 1983.63 | 16.79 |
| E | (+) | L1 | 26.59 | 26014.38 | 4.76 | 76.83 | 2464.92 | 155.36 | 2734.28 | 6419.12 | 55.33 |
|  |  | ± SE | 7.09 | 9949.85 | 2.38 | 21.35 | 489.35 | 42.56 | 883.90 | 1604.05 | 17.46 |
| E | (+) | L3 | 55.16 | 40092.92 | 10.06 | 143.03 | 6097.42 | 251.16 | 8968.15 | 12835.02 | 118.92 |
|  |  | ± SE | 13.93 | 11084.06 | 2.36 | 46.86 | 1738.56 | 107.51 | 1427.55 | 2987.37 | 30.18 |
| E | (+) | LR | 78.75 | 55095.18 | 16.10 | 211.49 | 9130.23 | 335.78 | 13732.33 | 19216.56 | 193.02 |
|  |  | ± SE | 50.73 | 30331.20 | 10.11 | 124.83 | 6054.21 | 197.25 | 8474.03 | 12700.19 | 130.08 |
| E | (+) | STEM | 40.16 | 18035.02 | 11.03 | 124.25 | 5179.23 | 116.09 | 8430.20 | 11894.47 | 122.20 |
|  |  | ± SE | 16.46 | 7313.35 | 4.52 | 60.19 | 2571.50 | 61.05 | 3851.36 | 5281.07 | 48.25 |
| E | (++) | Cot | 890.71 | 51336.17 | 11.41 | 58.26 | 5055.60 | 121.62 | 3902.15 | 12011.61 | 47.19 |
|  |  | ± SE | 321.91 | 24223.23 | 2.85 | 22.97 | 2146.46 | 44.67 | 1826.08 | 5340.03 | 23.05 |
| E | (++) | L1 | 1127.44 | 44845.74 | 8.29 | 92.90 | 6262.79 | 187.51 | 6107.91 | 14306.71 | 92.90 |
|  |  | ± SE | 102.59 | 5586.67 | 1.68 | 4.01 | 213.28 | 8.42 | 1449.02 | 1607.71 | 5.58 |
| E | (++) | L3 | 1339.47 | 41555.16 | 14.68 | 185.14 | 9771.12 | 214.43 | 15157.68 | 17914.51 | 183.21 |
|  |  | ± SE | 855.66 | 24481.82 | 7.04 | 130.48 | 6690.52 | 155.43 | 6855.51 | 10204.28 | 110.07 |
| E | (++) | LR | 1053.81 | 37691.53 | 9.24 | 92.41 | 6053.88 | 166.92 | 8324.14 | 12925.26 | 102.64 |
|  |  | ± SE | 89.75 | 4111.61 | 1.22 | 0.95 | 258.67 | 8.28 | 1711.87 | 1052.14 | 2.35 |
| E | (++) | STEM | 201.75 | 10032.71 | 8.10 | 82.30 | 3425.41 | 55.03 | 6044.50 | 9088.38 | 95.46 |
|  |  | ± SE | 27.86 | 2574.02 | 2.11 | 12.06 | 679.89 | 3.87 | 2173.47 | 1557.32 | 16.02 |
|  |  |  |  |  |  |  |  |  |  |  |  |
| E2 | (-) | Cot | 9.18 | 58685.71 | 5.93 | 86.46 | 6215.36 | 120.96 | 5148.57 | 10821.43 | 32.64 |
|  |  | ± SE | 4.68 | 16214.29 | 2.93 | 24.54 | 1276.07 | 51.32 | 75.71 | 2892.86 | 12.07 |
| E2 | (-) | L1 | 6.88 | 40107.31 | 6.04 | 108.10 | 4810.99 | 157.33 | 3137.82 | 10376.20 | 58.90 |
|  |  | ± SE | 3.04 | 11223.31 | 0.88 | 33.52 | 787.99 | 53.23 | 365.58 | 2444.20 | 11.14 |
| E2 | (-) | L3 | 5.13 | 33973.21 | 7.14 | 90.07 | 4688.69 | 127.50 | 3222.62 | 10614.58 | 64.66 |
|  |  | ± SE | 1.92 | 12366.07 | 0.24 | 12.22 | 1022.62 | 44.88 | 616.67 | 3049.11 | 3.47 |
| E2 | (-) | LR | 4.06 | 25365.60 | 6.31 | 74.01 | 4090.97 | 98.13 | 4041.34 | 9718.00 | 59.10 |
|  |  | ± SE | 1.13 | 4151.31 | 2.17 | 17.37 | 628.83 | 32.77 | 596.34 | 1468.00 | 15.88 |
| E2 | (-) | STEM | 8.86 | 11201.12 | 5.44 | 56.48 | 2439.67 | 36.23 | 3393.94 | 7998.83 | 60.80 |
|  |  | ± SE | 1.47 | 645.82 | 1.98 | 12.24 | 494.51 | 6.85 | 504.53 | 1567.50 | 12.14 |
| E2 | (+) | cot | 71.73 | 47087.22 | 32.10 | 141.12 | 3242.20 | 172.15 | 2650.12 | 6379.22 | 102.30 |
|  |  | ± SE | 26.79 | 10904.94 | 19.52 | 63.25 | 861.00 | 28.16 | 280.73 | 546.27 | 54.16 |
| E2 | (+) | L1 | 49.44 | 36108.97 | 12.34 | 110.60 | 3430.40 | 210.90 | 5019.92 | 9691.00 | 89.60 |
|  |  | ± SE | 12.22 | 9679.77 | 5.93 | 21.71 | 701.24 | 12.94 | 1949.63 | 2781.91 | 10.04 |
| E2 | (+) | L3 | 46.01 | 32688.55 | 11.04 | 101.01 | 3643.52 | 180.10 | 8163.90 | 9174.62 | 97.47 |
|  |  | ± SE | 4.26 | 2608.70 | 1.16 | 9.20 | 186.35 | 30.22 | 1546.71 | 669.52 | 5.94 |
| E2 | (+) | LR | 41.63 | 29693.23 | 9.17 | 87.98 | 3338.82 | 164.09 | 5185.84 | 9304.25 | 82.51 |
|  |  | ± SE | 10.14 | 10497.91 | 1.85 | 13.74 | 548.55 | 29.58 | 726.38 | 2903.34 | 13.73 |
| E2 | (+) | STEM | 32.03 | 15353.44 | 9.28 | 70.19 | 3032.09 | 61.37 | 7813.36 | 9175.51 | 88.84 |
|  |  | ± SE | 3.38 | 2941.46 | 0.74 | 2.18 | 85.30 | 5.37 | 1632.99 | 688.65 | 3.80 |
| E2 | (++) | Cot | 883.18 | 42165.52 | 11.18 | 50.24 | 3935.09 | 134.97 | 2437.31 | 10394.85 | 36.83 |
|  |  | ± SE | 566.82 | 32580.48 | 8.53 | 30.10 | 2993.07 | 96.31 | 1897.83 | 7308.67 | 28.99 |
| E2 | (++) | L1 | 1034.36 | 45534.25 | 6.00 | 83.99 | 5764.30 | 204.66 | 4970.63 | 15501.24 | 78.87 |
|  |  | ± SE | 124.04 | 10714.30 | 2.42 | 12.08 | 1097.20 | 29.87 | 802.29 | 2248.86 | 11.77 |
| E2 | (++) | L3 | 1239.87 | 43642.89 | 8.66 | 135.47 | 8211.54 | 206.70 | 12524.56 | 15953.01 | 143.46 |
|  |  | ± SE | 595.56 | 18611.63 | 2.50 | 55.80 | 3818.71 | 87.62 | 5224.60 | 6605.90 | 70.43 |
| E2 | (++) | LR | 1592.98 | 58439.72 | 11.19 | 135.27 | 8696.65 | 313.12 | 10249.17 | 19937.30 | 150.10 |
|  |  | ± SE | 663.46 | 23857.29 | 3.73 | 53.67 | 3852.85 | 140.45 | 5823.82 | 7630.81 | 71.83 |
| E2 | (++) | STEM | 326.47 | 14866.75 | 6.70 | 79.76 | 3845.55 | 76.75 | 6320.84 | 8498.67 | 91.07 |
|  |  | ± SE | 102.80 | 3831.18 | 2.44 | 20.22 | 1054.94 | 24.66 | 2105.61 | 1935.83 | 27.92 |
|  |  |  |  |  |  |  |  |  |  |  |  |
| IE | (-) | Cot | 3.72 | 58389.26 | 8.81 | 114.56 | 8197.38 | 76.22 | 6604.92 | 22952.58 | 103.64 |
|  |  | ± SE | 2.83 | 29080.31 | 2.20 | 52.71 | 4137.66 | 37.34 | 3027.28 | 11653.55 | 62.68 |
| IE | (+) | Cot | 24.95 | 24921.55 | 2.97 | 71.47 | 2652.00 | 115.86 | 1774.01 | 6635.34 | 28.46 |
|  |  | ± SE | 6.47 | 11252.56 | 1.73 | 29.65 | 1304.72 | 59.01 | 1091.62 | 3151.84 | 10.67 |
| IE | (+) | L1 | 62.68 | 46795.57 | 8.62 | 137.84 | 6314.50 | 243.76 | 2988.31 | 19075.83 | 106.28 |
|  |  | ± SE | 32.61 | 22340.39 | 4.22 | 72.72 | 3253.28 | 108.07 | 1007.08 | 8623.38 | 58.74 |
| IE | (+) | L3 | 22.75 | 14871.14 | 5.10 | 58.04 | 2634.72 | 76.95 | 2420.22 | 8117.11 | 58.64 |
|  |  | ± SE | 2.45 | 2112.19 | 1.08 | 5.50 | 370.64 | 10.27 | 1017.70 | 1144.25 | 2.41 |
| IE | (+) | LR | 47.70 | 35438.23 | 9.15 | 105.40 | 5442.35 | 187.10 | 2919.91 | 17041.28 | 101.82 |
|  |  | ± SE | 16.96 | 14582.61 | 2.95 | 40.37 | 2264.09 | 73.97 | 506.75 | 5725.57 | 43.74 |
| IE | (+) | STEM | 22.06 | 11041.12 | 6.05 | 57.27 | 3267.05 | 54.62 | 1995.78 | 11455.78 | 85.21 |
|  |  | ± SE | 4.59 | 2085.24 | 0.44 | 6.88 | 691.53 | 8.78 | 376.51 | 2215.33 | 21.01 |
| IE | (++) | Cot | 605.22 | 36184.05 | 3.10 | 47.43 | 3385.84 | 95.12 | 2815.07 | 11759.31 | 22.33 |
|  |  | ± SE | 348.96 | 30649.03 | 2.60 | 36.01 | 2391.71 | 61.86 | 2119.60 | 8487.53 | 8.83 |
| IE | (++) | L1 | 662.13 | 32879.58 | 7.44 | 81.14 | 4533.61 | 158.24 | 4126.36 | 18012.66 | 92.99 |
|  |  | ± SE | 185.10 | 10437.20 | 2.18 | 22.20 | 1128.31 | 32.40 | 1720.67 | 4212.77 | 21.85 |
| IE | (++) | L3 | 780.87 | 42486.98 | 22.11 | 174.74 | 7858.03 | 254.10 | 9055.52 | 30142.48 | 242.90 |
|  |  | ± SE | 137.01 | 10317.73 | 6.64 | 52.52 | 2145.76 | 80.72 | 3083.32 | 9536.79 | 92.88 |
| IE | (++) | LR | 2365.16 | 91980.99 | 41.88 | 293.34 | 14063.66 | 538.22 | 12243.72 | 61251.21 | 386.05 |
|  |  | ± SE | 1019.04 | 25970.35 | 20.83 | 73.45 | 5390.92 | 250.93 | 6475.19 | 22780.89 | 193.07 |
| IE | (++) | STEM | 176.47 | 11418.90 | 8.76 | 68.87 | 3097.27 | 65.91 | 3714.06 | 12359.82 | 100.03 |
|  |  | ± SE | 96.00 | 3939.90 | 3.39 | 23.99 | 829.77 | 24.01 | 1688.67 | 4349.15 | 31.23 |
|  |  |  |  |  |  |  |  |  |  |  |  |
| IE2 | (-) | Cot | 2.20 | 28902.70 | 4.23 | 53.46 | 4684.71 | 28.10 | 3402.59 | 6188.18 | 28.20 |
|  |  | ± SE | 0.44 | 4455.02 | 1.19 | 9.92 | 838.69 | 9.19 | 132.14 | 1416.86 | 11.23 |
| IE2 | (+) | Cot | 46.99 | 37916.65 | 10.30 | 71.48 | 3539.74 | 125.28 | 2923.51 | 10337.24 | 28.48 |
|  |  | ± SE | 24.16 | 19310.51 | 0.82 | 32.30 | 1871.58 | 66.97 | 1532.04 | 5282.08 | 10.27 |
| IE2 | (+) | L1 | 52.16 | 39488.67 | 11.79 | 116.11 | 4302.00 | 189.15 | 2982.13 | 15990.53 | 102.86 |
|  |  | ± SE | 6.14 | 2518.49 | 0.51 | 16.27 | 368.26 | 25.60 | 996.47 | 939.42 | 14.86 |
| IE2 | (+) | L3 | 34.10 | 21283.47 | 12.81 | 86.24 | 3659.90 | 95.93 | 2726.24 | 12228.17 | 104.43 |
|  |  | ± SE | 6.81 | 6353.91 | 3.18 | 17.51 | 841.02 | 12.63 | 1194.44 | 2857.02 | 17.57 |
| IE2 | (+) | LR | 78.09 | 50709.79 | 20.61 | 180.46 | 6957.67 | 265.85 | 3939.80 | 24690.34 | 182.64 |
|  |  | ± SE | 36.48 | 13462.65 | 9.88 | 92.00 | 2851.96 | 154.65 | 619.75 | 9938.08 | 108.24 |
| IE2 | (+) | STEM | 36.23 | 24112.79 | 12.40 | 81.86 | 4465.87 | 75.99 | 3513.72 | 14086.36 | 124.48 |
|  |  | ± SE | 1.51 | 1262.40 | 1.89 | 8.68 | 405.04 | 14.96 | 1061.31 | 1112.92 | 23.54 |
| IE2 | (++) | Cot | 1784.34 | 64976.25 | 4.05 | 94.05 | 8860.65 | 193.62 | 3891.02 | 28709.51 | 58.53 |
|  |  | ± SE | 893.53 | 35819.96 | 2.16 | 50.04 | 5095.28 | 80.24 | 2030.48 | 14321.93 | 24.44 |
| IE2 | (++) | L1 | 1018.55 | 35455.26 | 8.88 | 92.08 | 5862.46 | 146.59 | 3100.83 | 19129.55 | 89.35 |
|  |  | ± SE | 492.04 | 11962.39 | 3.50 | 34.64 | 2257.03 | 59.68 | 1841.42 | 7182.48 | 37.84 |
| IE2 | (++) | L3 | 528.60 | 27443.14 | 12.71 | 85.09 | 5463.56 | 97.53 | 3337.14 | 15684.75 | 115.94 |
|  |  | ± SE | 336.46 | 15735.12 | 7.54 | 44.00 | 2777.26 | 48.68 | 1571.01 | 7908.91 | 57.19 |
| IE2 | (++) | LR | 877.17 | 31565.42 | 10.21 | 90.71 | 5659.36 | 126.31 | 3227.79 | 17683.17 | 104.39 |
|  |  | ± SE | 295.54 | 10383.56 | 2.60 | 22.87 | 1717.05 | 24.50 | 1169.73 | 4714.04 | 30.35 |
| IE2 | (++) | STEM | 70.33 | 7836.66 | 4.30 | 31.87 | 2032.46 | 24.30 | 1545.72 | 6931.72 | 51.16 |
|  |  | ± SE | 35.30 | 1977.58 | 2.17 | 10.81 | 352.84 | 6.64 | 196.82 | 1392.62 | 14.33 |
