# Supplementary material for: Identification of Rapeseed (Brassica napus) Cultivars With a High Tolerance to Boron-Deficient Conditions
Source: Front Plant Sci. 2018 Aug 7;9:1142. doi: 10.3389/fpls.2018.01142 (PMC6091279; doi:10.3389/fpls.2018.01142)

**Supplementary_Data_Sheet_S11: Nitrogen efficiency test of IE and E on zerosoil-substrate.** N-efficiency of *CR2262* (= IE) and *CR2267* (= E) during the first 15 days of growth after germination (DAG). Plants were grown under sufficient nitrogen conditions (N 1) or under nitrogen N deficiency (N 0, arrows). Error bars show ± SD from 6 plants. Bars = 1 cm.


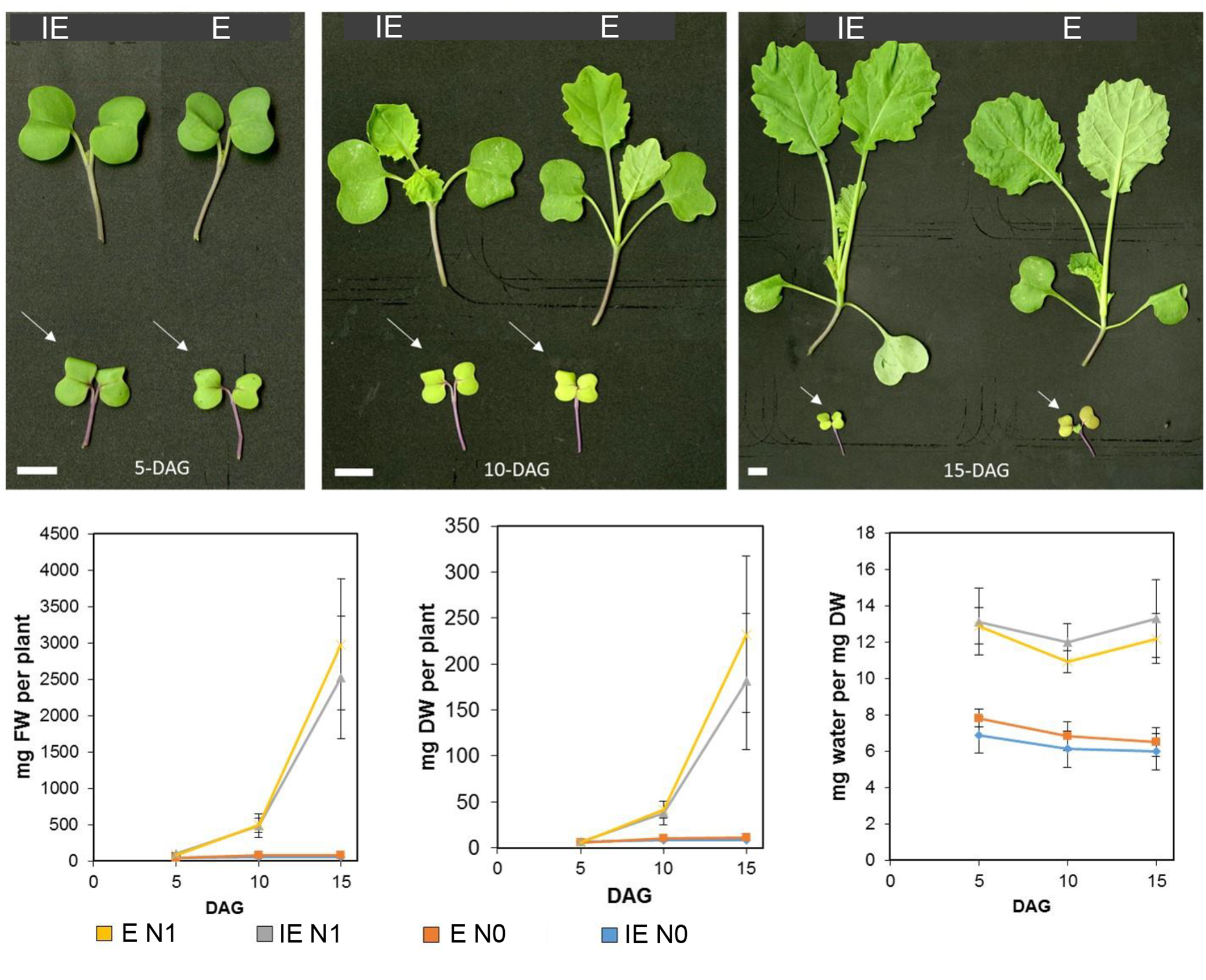

Supplement: Supplementary file 11 [file Data_Sheet_11.docx]
